# Enantioselective palladium/copper-catalyzed C–C σ-bond activation synergized with Sonogashira-type C(sp^3^)–C(sp) cross-coupling alkynylation†
†Electronic supplementary information (ESI) available. CCDC 1910015. For ESI and crystallographic data in CIF or other electronic format see DOI: 10.1039/c9sc02431j


**DOI:** 10.1039/c9sc02431j

**Published:** 2019-06-21

**Authors:** Feng-Na Sun, Wan-Chun Yang, Xiao-Bing Chen, Yu-Li Sun, Jian Cao, Zheng Xu, Li-Wen Xu

**Affiliations:** a Key Laboratory of Organosilicon Chemistry and Material Technology of Ministry of Education , Key Laboratory of Organosilicon Material Technology of Zhejiang Province , Hangzhou Normal University , P. R. China . Email: caojian@hznu.edu.cn ; Email: liwenxu@hznu.edu.cn; b State Key Laboratory for Oxo Synthesis and Selective Oxidation , Suzhou Research Institute , Lanzhou Institute of Chemical Physics , Chinese Academy of Sciences , P. R. China

## Abstract

The Sonogashira-type cross-coupling reaction is one of the most significant alkynylation transformations in organic chemistry. However, highly enantioselective alkynylation *via* the Sonogashira-type cross-coupling reaction is rather limited, mainly due to the difficulties in matching the stereoselective induction of chiral ligands with the combinational behavior of Pd/Cu-based bimetallic catalysts. We herein report novel enantioselective palladium/copper-catalyzed alkyl alkynylation of cyclobutanones with terminal alkynes *via* tandem C–C bond activation/Sonogashira-type cross coupling reaction, in which a novel chiral TADDOL-derived phosphoramidite ligand bearing fluorine and silicon-based bulky groups simplified as TFSi-Phos is found to be an efficient ligand for both C(sp^2^)–C(sp^3^) bond cleavage and new C(sp^3^)–C(sp) bond formation. A wide range of chiral alkynylated indanones bearing an all-carbon quaternary stereocenter are obtained efficiently with up to 97.5 : 2.5 er.

## Abstract 2

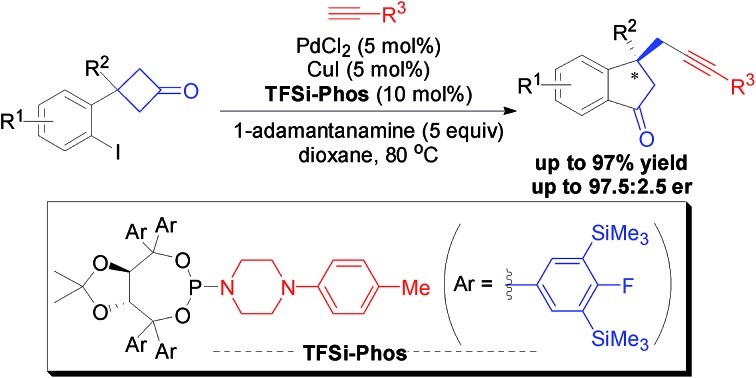
A highly enantioselective Pd/Cu-catalyzed tandem C–C bond activation/Sonogashira reaction was developed with the aid of new TFSi-Phos, which provided chiral alkynyl indanones in good yields and enantioselectivities.

## Introduction

**Scheme 1:**
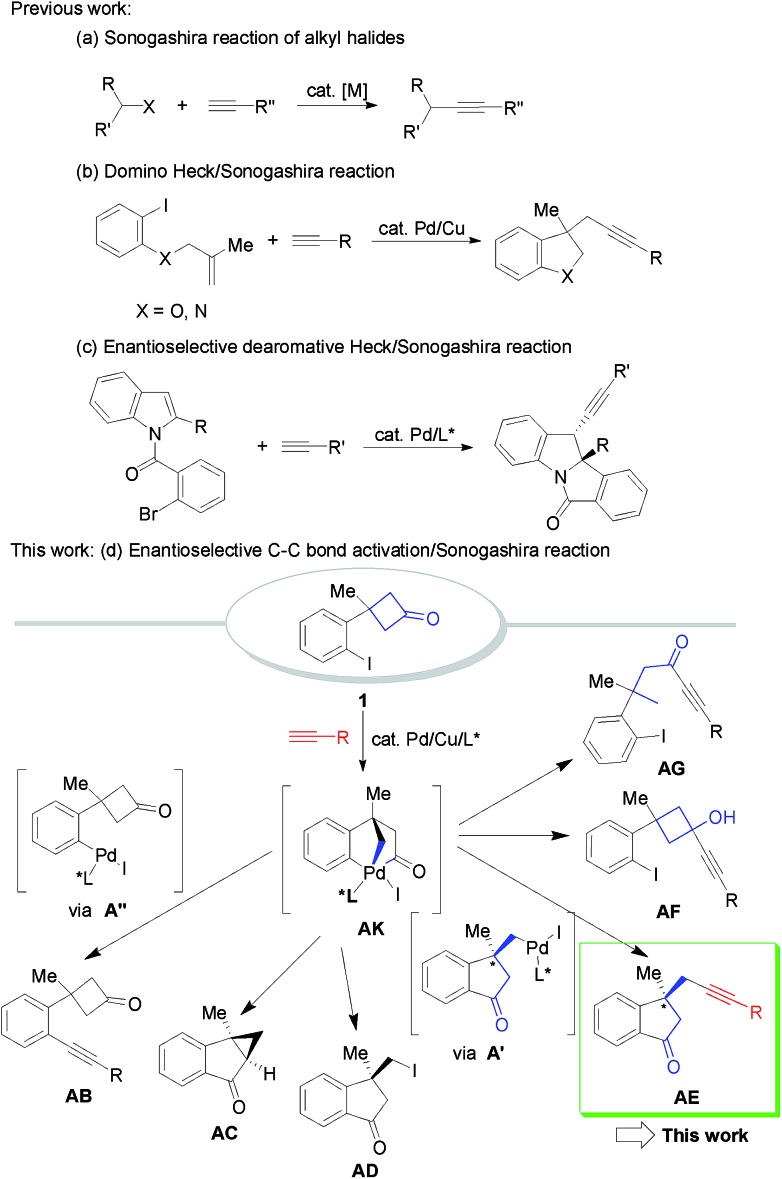
Possible transformations for the alkynylation of cyclobutanone-containing aryl iodide.

The C–C triple bond is one of the most significant functionalities with a rich reactivity profile in the arena of organic chemistry and other versatile fields, including medicinal chemistry, materials science, physical photonics, supramolecular chemistry, and so on.^1^ As a fundamental synthon, alkynes are involved in a variety of important and useful synthetic transformations for the construction of functional molecules.^2^ In this regard, one of the most direct approaches for the incorporation of alkynyl moieties into organic substrates is the direct metalation of terminal alkynes and their application in the alkynylation of alkyl/aryl halides, carbonyl compounds, and other electrophiles, as especially found in the classical Sonogashira-type alkynylation reactions.^3^ Sonogashira-type cross-coupling represents a powerful tool for the synthesis of differently substituted internal alkynes.^4^ In addition to traditional coupling between aryl/vinyl halides and terminal alkynes, Sonogashira-type cross-coupling of alkyl halides has also been established using Pd/Cu or Ni/Cu catalytic systems in the past few decades (Scheme 1a).^5^–^7^ An alternative approach to alkynylation by the formation of C(sp^3^)–C(sp) bonds involves sequential intramolecular carbopalladation of alkenes and trapping of the transient σ-alkylpalladium species by terminal alkynes, in which the diverse Pd-catalyzed 1,2-carboalkynylation of alkenes to produce alkyl-substituted alkynes containing heterocycles such as indolines and dihydrobenzofurans has been reported using this strategy (Scheme 1b).^8^–^11^ Despite there being no reports on the enantioselective Sonogashira-type C(sp^3^)–C(sp) alkynylation reaction, enantioselective domino Heck/σ-alkylpalladium species capture by nucleophiles such as cyanide, azole, hydride and isocyanide/MeOH has been studied by the groups of Zhu,^12^ Lautens,^13^ Jia,^14^ Diaz,^15^ and Zhang.^16^ Interestingly, the corresponding enantioselective alkynylation by Sonogashira-type cross-coupling/nucleophilic capture is rather limited. Until now only one enantioselective dearomative Heck/Sonogashira reaction of indoles has been achieved by Jia and co-workers (Scheme 1c).^17^ Similar to the catalytic chemistry of the cross-coupling/anion capture sequence, transition-metal catalyzed C–C σ-bond activation and new C–C bond-formation reactions have emerged as a vibrant field in the last decade.^18^ In this respect, C–C σ-bond activation of cyclobutanones received considerable attention owing to their inherent high ring strain and rigid conformation. The groups of Murakami,^19^ Cramer,^20^ and Dong^21^ developed Rh- and Ni-catalyzed enantioselective C–C bond activation of prochiral cyclobutanones. Pd-catalyzed racemic C–C bond activation reaction patterns of cyclobutanones were performed by Murakami and co-workers with achiral catalytic systems.^22^ Very recently, we reported highly enantioselective tandem C–C bond activation/C(sp^3^)–C(sp^2^)/C(sp^3^)–I bond forming reactions of cyclobutanones.^23^ However, to the best of our knowledge, there are no reports on the enantioselective palladium-controlled carbon–carbon bond cleavage of cyclobutanones for the diverse synthesis of chiral alkynylated indanones by trapping of the transient σ-alkylpalladium species with terminal alkynes. In fact, facilitating an enantioselective tandem C–C bond activation/Sonogashira-type C(sp^3^)–C(sp) cross-coupling alkynylation presents several fundamental challenges. (1) The Sonogashira-type C(sp^3^)–C(sp) cross-coupling reaction uses palladium and copper as bimetallic catalysts, and the stereoselective induction of the chiral ligand would be affected by the competitive coordination. There are no reports on the catalytic asymmetric synthesis synergized with the Sonogashira-type C(sp^3^)–C(sp) cross-coupling reaction. (2) Inherently, no new carbon-stereogenic centers are formed in Sonogashira-type C(sp^3^)–C(sp) cross-coupling alkynylation. This reaction requires the formation of chiral intermediates beyond the formed alkyne-containing product. The enantioselective C–C bond activation/Sonogashira-type C(sp^3^)–C(sp) cross-coupling transformation must outcompete the corresponding background reaction. Thus it is really difficult to control the stereochemical transmission information in the presence of bimetallic Pd/Cu and terminal alkynes. Therefore, the development of a highly enantioselective tandem C–C bond activation/Sonogashira-type C(sp^3^)–C(sp) cross-coupling alkynylation is not a trivial task.

Herein, we present a distinct palladium-catalyzed enantioselective tandem carbon–carbon bond activation/Sonogashira-type C(sp^3^)–C(sp) cross-coupling alkynylation reaction of cyclobutanones and terminal alkynes (Scheme 1d) which can be enabled by bimetallic catalysis with the aid of a novel P-ligand.

## Results and discussion

To capture the highly reactive σ-alkylpalladium species formed by palladium-induced C–C bond activation, we expect that the key intermediate σ-alkylpalladium species **AK** generated during the C–C bond activation process could be trapped by terminal alkynes to form different C(sp^3^)–C(sp) bonds (Scheme 1, more than six pathways). Especially for the desired **AK**–**A′**–**AE** pathway, the whole process would provide alkyl-substituted alkynes containing indanones bearing an all-carbon quaternary stereocenter,^24^ which is an important class of structural motifs frequently found in natural products and pharmaceuticals.^25^ However, several issues need to be addressed to achieve the proposal: (1) side reactions such as direct Sonogashira cross-coupling, intramolecular cyclopropanation, and C–I bond reductive elimination leading to undesired side products (**AB**–**AG**) must be inhibited. (2) It is challenging to develop enantioselective sequential reactions involving nucleophilic trapping of σ-alkylpalladium species because any neutral or ionic nucleophilic species present in the reaction system could potentially coordinate to Pd, thereby affecting the asymmetric environment created by the chiral ligand. Thus, identifying a catalytic system which can chemoselectively promote C(sp^2^)–C(sp^3^) bond activation and C(sp^3^)–C(sp) bond formation and at the same time obtain high enantioselectivity is a formidable challenge. As a continuation of our research on the synthesis and reactions of four-membered rings,^26^ we report a novel Pd/Cu-catalyzed enantioselective alkyl alkynylation *via* tandem C(sp^2^)–C(sp^3^) bond activation/Sonogashira reaction of cyclobutanones with terminal alkynes.

**Scheme 2:**
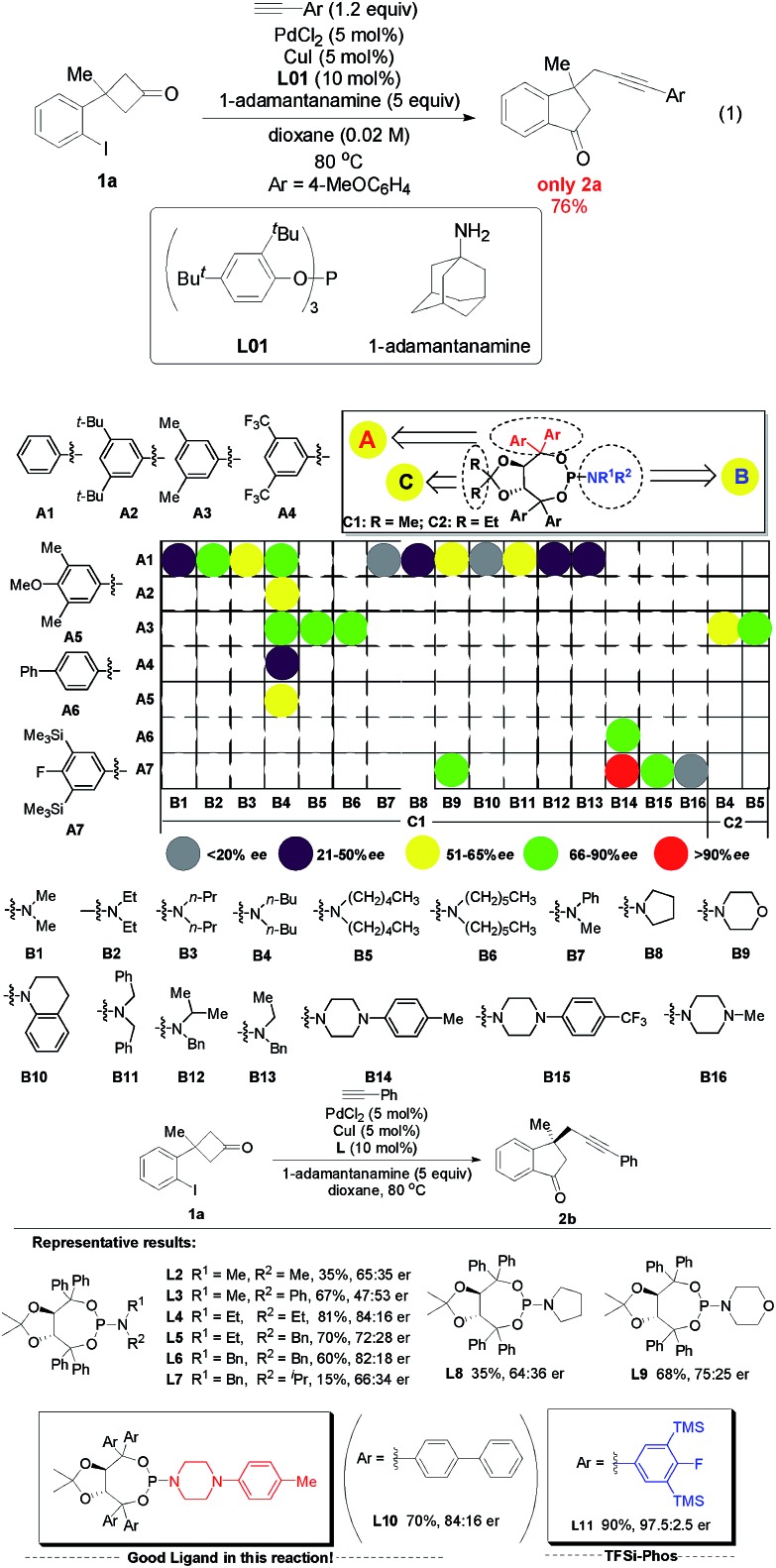
Screening of chiral ligands: identification of a novel F/Si/N-based TADDOL-derived phosphoramidite ligand **L11** (TFSi-Phos) precisely regulated by steric repulsion and electronic effects.

At the outset, 3-(2-iodophenyl)-3-methylcyclobutanone **1a** and 4-methoxyphenylacetylene were used as the model substrates to examine the feasibility of our hypothesis. After a systematic survey of the reaction parameters, the following optimum conditions were identified: PdCl_2_ (0.05 equiv.), CuI (0.05 equiv.), ligand **L01** (0.1 equiv.), 1-adamantanamine (5 equiv.), and 1,4-dioxane, 80 °C, affording the desired racemic 1-indanone **2a** in 76% yield (eqn (1) of Scheme 2. For detailed screening of the reaction conditions, see Table S1 in the ESI†). Several features can be concluded: (1) in the absence of PdCl_2_, CuI or ligands, no reaction occurred or poor yields with low chemoselectivity (Scheme 1) were obtained, indicating the vital role of PdCl_2_, CuI, and ligands in the catalytic cycle. (2) The structure of ligands had a significant impact on this transformation. Bidentate ligands were ineffective while bulky monodentate phosphine ligands gave better yields. (3) Common tertiary and secondary amines gave low yields and bulky primary amines such as 1-adamantanamine were suitable for this reaction. (4) Low concentration inhibited direct Sonogashira cross-coupling transformation and increased the yield of the desired product.

Subsequently, a range of chiral monodentate phosphine ligands were examined to realize the enantioselective C–C bond activation/Sonogashira reaction (Scheme 2). According to our previous experience in chiral P-ligands^27^ and enantioselective Pd-catalyzed C–C bond activation of cyclobutanones, we started with TADDOL-derived phosphoramidites (Scheme 2, >24 TADDOL-derived P-ligands).^28^ For example, a simple phosphoramidite **L2** gave low yield and enantioselectivity.^29^ And subsequent modification of the amine moiety only resulted in moderate yields and enantioselectivity (**L4**, 81%, 84 : 16 er). Compared to various known phosphoramidites ligands, we found that **L10** reported by Gu and co-workers showed promising results.^30^ These experimental data supported that the design and preparation of TADDOL-derived P-ligands bearing different substituents is a mammoth undertaking, in which the precision control of steric repulsion and electronic effects is not trivial. On the basis of screened experimental results, further modification focused on the variation of the aryl groups led to the determination of the newly designed ligand **L11** (simplified as TFSi-Phos) with bulky aryl groups bearing TMS and a fluorine atom. It was proven to be the best choice, leading to the desired product in 90% yield with 97.5 : 2.5 er.

**Scheme 3:**
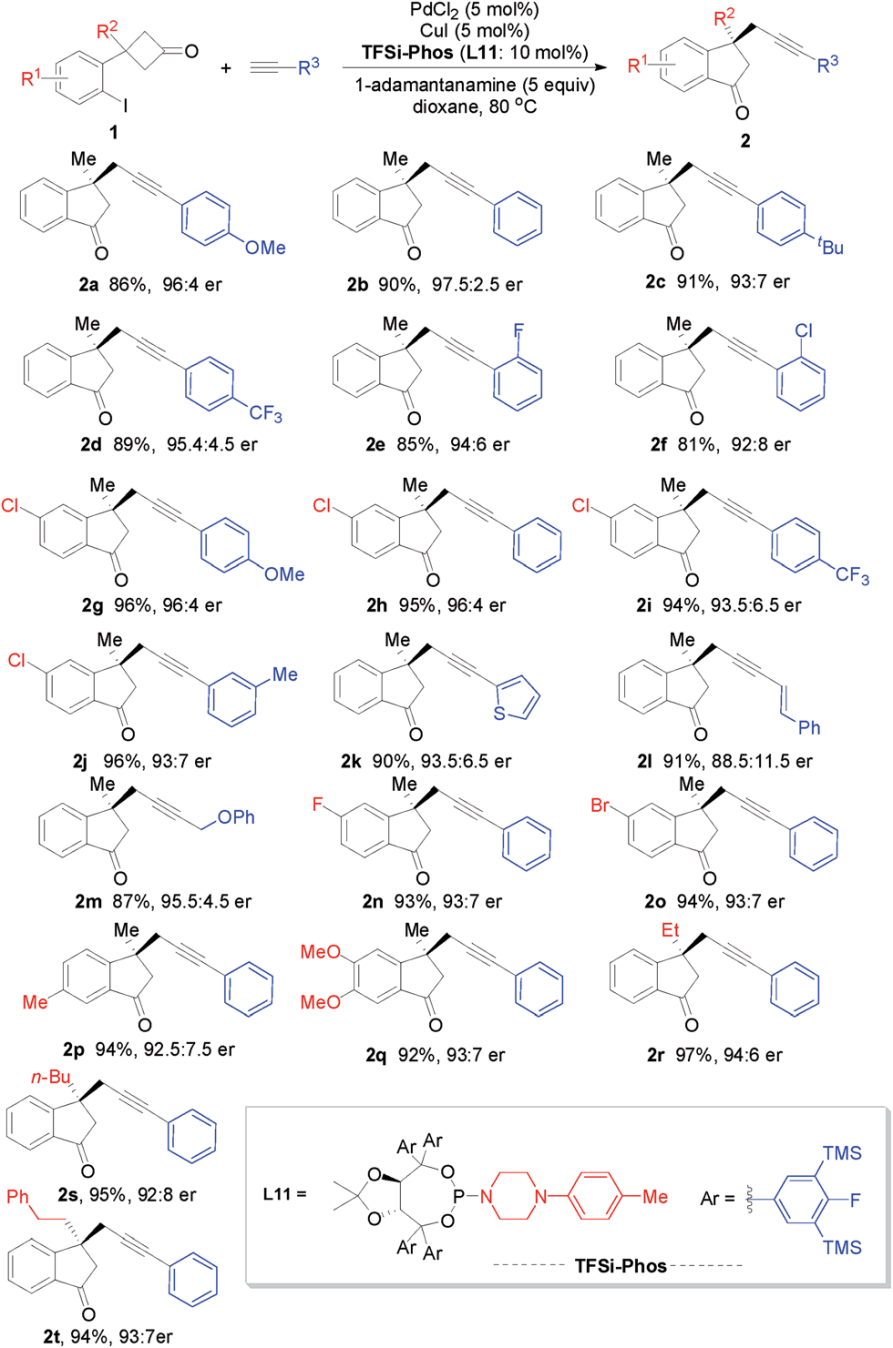
Substrate scope for Pd/Cu-catalyzed C–C bond activation. The reactions were run on a 0.2 mmol scale in 10.0 mL solvent for 12 h.

With the optimized reaction conditions in hand, we next investigated the substrate scope and limitation of this enantioselective Pd/Cu-catalyzed alkyl alkynylation *via* tandem C–C bond activation/Sonogashira reaction. As depicted in Scheme 3, various terminal alkynes proved to be amenable to this transformation. Arylacetylene bearing a series of substituents on the phenyl ring, including MeO, ^*t*^Bu, Me, CF_3_, F, and Cl, reacted efficiently to afford the corresponding products in good yields (81–96%) with 92 : 8–97.5 : 2.5 er (**2a–2j**). Heteroaromatic groups such as 2-thiophenyl were also well tolerated (**2k**). In addition, an enyne moiety could be easily incorporated into the product **2l** albeit with lower enantioselectivity. Aliphatic alkynes reacted smoothly to afford the desired product **2m** in 87% yield with 95 : 5 er. On the other hand, the scope with respect to cyclobutanones was also examined. Substituents on the phenyl moiety of cyclobutanones, such as Me, MeO, F, Cl, and Br, were all amenable to this transformation, leaving ample room for further functionalization (**2n–2q**). Furthermore, cyclobutanones bearing various alkyl groups at the R^2^ position were compatible (**2r–2t**).

**Scheme 4:**
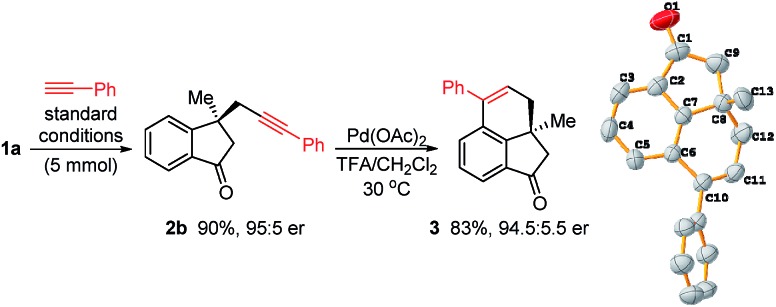
Gram-scale reaction and synthetic applications.

To demonstrate the practicability of this reaction, a gram-scale experiment (5 mmol) was conducted and **2b** was obtained in 90% yield with 95 : 5 er (Scheme 4). Carbon–carbon triple bonds are versatile functional groups and various transformations can be considered for downstream derivatization. For example, Pd-catalyzed intramolecular cyclization of **2b** in TFA/DCM led to compound **3** without racemization. The absolute configuration of **3** was unambiguously determined by single-crystal X-ray diffraction analysis.^31^ The structures of other products were tentatively assigned based on the configuration of **3**.

**Scheme 5:**
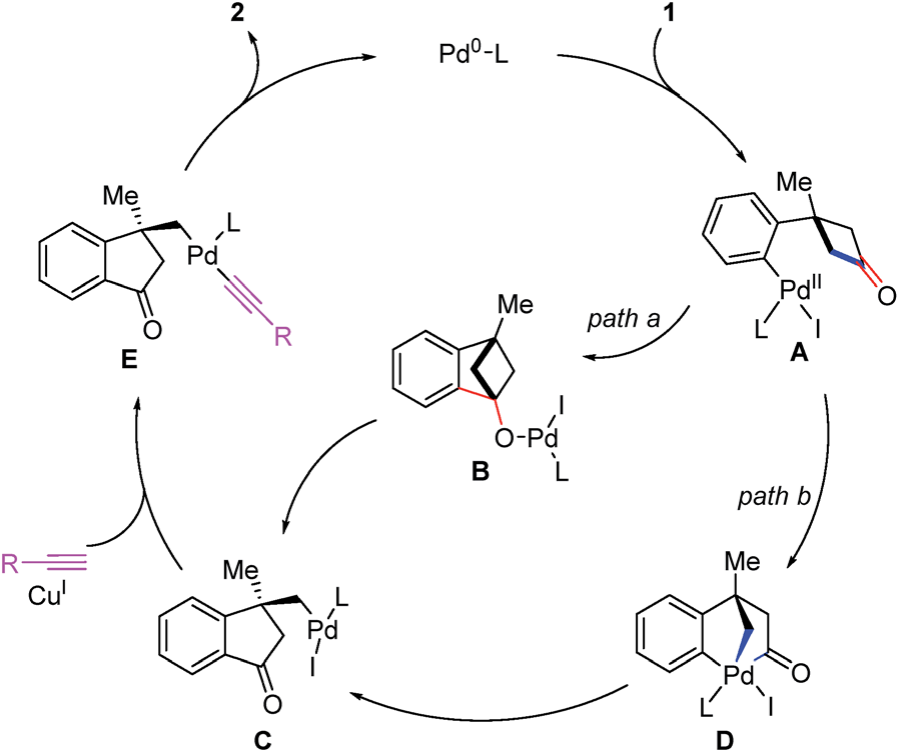
Proposed catalytic cycles.

According to our previous density-functional theory (DFT) calculation studies on the σ-bond cross-exchange reaction,23*b* two plausible pathways were proposed for this enantioselective alkyl alkynylation through tandem C–C bond activation/Sonogashira reaction (Scheme 5). Cyclobutanones **1** undergo oxidative addition with a Pd^0^ complex to form arylpalladium species **A**. Then two possible pathways for the ring-opening process of cyclobutanones can be expected. (1) Nucleophilic addition of arylpalladium species toward carbonyl group generates an alkoxypalladium intermediate **B**. Subsequent enantioselective β-carbon elimination leads to σ-alkylpalladium species **C** (path a). (2) **A** would undergo a second oxidative addition with one of the C(sp^2^)–C(sp^3^) bonds of cyclobutanone to form Pd^IV^ intermediate **D**. Then a facile C(sp^2^)–C(sp^2^) reductive elimination gives the complex **C** (path b). Subsequently, σ-alkylpalladium species **C** is captured by terminal alkynes with the help of the CuI catalyst to form the intermediate **E**, which gives 1-indanone **2** through reductive elimination.

## Conclusions

In summary, we disclosed palladium-catalyzed enantioselective alkyl alkynylation *via* sequential C–C bond activation/Sonogashira reaction of cyclobutanones. This is a novel enantioselective palladium/copper-catalyzed alkynylation of cyclobutanones with terminal alkynes. In this reaction, a wide range of chiral alkynylated indanones bearing an internal alkyne moiety and an all-carbon quaternary stereocenter are provided efficiently with up to 97.5 : 2.5 er, in which our chiral TADDOL-derived novel phosphoramidite ligand with fluorine and silicon-based bulky groups simplified as TFSi-Phos is found to be an efficient ligand for both C–C bond cleavage and C(sp^3^)–C(sp) bond formation. Further investigation involving the widespread application of the novel TFSi-Phos ligand that could possibly work as a structurally new TADDOL-derived bidentate P,N-ligand, and the detailed mechanistic elucidation of the corresponding TFSi-Phos-involved bimetallic catalysis will be carried out and reported in the near future.

## Conflicts of interest

There are no conflicts to declare.

## Supplementary Material

Supplementary information

Crystal structure data
