# Supplementary material for: Enantioselective palladium/copper-catalyzed C–C σ-bond activation synergized with Sonogashira-type C(sp3)–C(sp) cross-coupling alkynylation
Source: Chem Sci. 2019 Jun 21;10(32):7579–83. doi: 10.1039/c9sc02431j (PMC6761865; doi:10.1039/c9sc02431j)
Supplement: Supplementary file 1 [file SC-010-C9SC02431J-s001.pdf]

Electronic Supplementary Material (ESI) for Chemical Science.
This journal is © The Royal Society of Chemistry 2019

Supporting Information

Enantioselective Palladium/Copper-Catalyzed C-C σ-Bond Activation Synergized with Sonogashira-Type C(sp^3)-C(sp) Cross-Coupling Alkynylation

Feng-Na Sun[1], Wan-Chun Yang[1], Xiao-Bing Chen[1], Yu-Li Sun[1], Jian Cao*[,1], Zheng Xu[1], and Li-Wen Xu*[,1,2]

[1] Key Laboratory of Organosilicon Chemistry and Material Technology of Ministry of Education, and and Key Laboratory of Organosilicon Material Technology of Zhejiang Province, Hangzhou Normal University (HZNU), No 1378, Wenyi West Road, Science Park of HZNU, Hangzhou 311121, P. R. China.

[2] State Key Laboratory for Oxo Synthesis and Selective Oxidation, Suzhou Research Institute and Lanzhou Institute of Chemical Physics, Chinese Academy of Sciences, P. R. China

Correspondence and requests for materials should be addressed to J.C. (email: caojian@hznu.edu.cn) and L.W.X (email: liwenxu@hznu.edu.cn)

A. General information S-2
Table S1: Selected optimization of racemic ring-opening/Sonogashira reaction S-3
B. Preparation of chiral ligands S-6
C. Synthesis of 1-indanones 2 S-7
D. Synthesis of 3 S-35
E. References S-37
F. NMR spectra S-38

General information

^{1}H NMR (400 MHz) and ^{13}C NMR (100 MHz) spectra were recorded on a Bruker Avance (400 MHz) spectrometer, using $CDCl_3$ as the solvent and TMS as internal standard; chemical shifts were quoted in parts per million and *J* values were given in hertz. High resolution mass spectrometry (HRMS) was performed on a Waters Micromass GCT. HPLC was carried out on an Agilent 1260 infinity or Waters 2695 instrument using a chiralpak AS-H, AD-H, OD-H, OJ-H, or OX-H column. All solvents were dried according to standard procedures.

Table S1: Selected optimization of racemic ring-opening/Sonogashira reaction.[a]

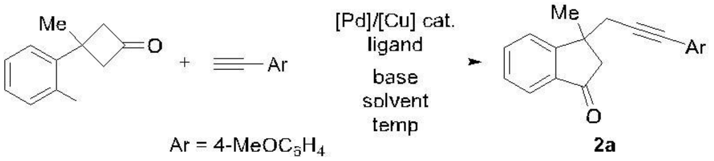

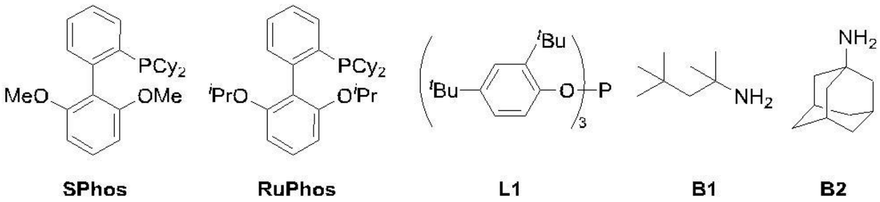

entry	[Pd]	ligand	base	solvent	temp (^{o}C)	yield of **2a** (%)
1	$PdCl_2(PPh_3)_2$	/	Et_3N (10 equiv)	dixoane	80	0
2	$PdCl_2$	**SPhos**	Et_3N (10 equiv)	dixoane	80	28
3	$PdCl_2$	**RuPhos**	Et_3N (10 equiv)	dixoane	80	<5
4	$PdCl_2$	**L1**	Et_3N (10 equiv)	dixoane	80	25
5	$Pd(OAc)_2$	**L1**	Et_3N (10 equiv)	dixoane	80	<5
6	$PdBr_2$	**L1**	Et_3N (10 equiv)	dixoane	80	<5
7	$[Pd(allyl)Cl]_2$	**L1**	Et_3N (10 equiv)	dixoane	80	<5
8	$PdCl_2$	**L1**	Et_3N (10 equiv)	CH_3CN	80	<5
9	$PdCl_2$	**L1**	Et_3N (10 equiv)	toluene	80	11
10	$PdCl_2$	**L1**	Et_3N (10 equiv)	DCE	80	<5
11	$PdCl_2$	**L1**	Et_3N (10 equiv)	DMF	80	<5
12	$PdCl_2$	**L1**	$^{i}Pr_2NH$ (10 equiv)	dixoane	80	30
13	$PdCl_2$	**L1**	Cy_2NH (10 equiv)	dixoane	80	26
14	$PdCl_2$	**L1**	$^{t}BuNH_2$ (10 equiv)	dixoane	80	41
15	$PdCl_2$	**L1**	$^{t}BuNH_2$ (10 equiv)	dixoane	rt	<5
16	$PdCl_2$	**L1**	$^{t}BuNH_2$ (10 equiv)	dixoane	50	17
17	$PdCl_2$	**L1**	$^{t}BuNH_2$ (10 equiv)	dixoane	100	32
18	$PdCl_2$	**L1**	$^{t}BuNH_2$ (10 equiv)	dixoane	120	37
19	$PdCl_2$	**L1**	$^{t}BuNH_2$ (2 equiv)	dixoane	80	29
20	$PdCl_2$	**L1**	$^{t}BuNH_2$ (5 equiv)	dixoane	80	44
21	$PdCl_2$	**L1**	$^{t}BuNH_2$ (20 equiv)	dixoane	80	18
22	$PdCl_2$	**L1**	**B1** (10 equiv)	dixoane	80	57
23	$PdCl_2$	**L1**	**B2** (10 equiv)	dixoane	80	53
24[b]	$PdCl_2$	**L1**	**B2** (5 equiv)	dixoane	80	65
**25[c]**	**$PdCl_2$**	**L1**	**B2 (5 equiv)**	**dixoane**	**80**	**76**
26[d]	$PdCl_2$	**L1**	**B2** (5 equiv)	dixoane	80	60

[a]Unless otherwise noted, the reactions were carried out using **1a** (0.2 mmol), alkyne (1.2 equiv), [Pd] (5.0 mol%), CuI (5.0 mol%), ligand (10.0 mol%), and base in solvent (2 mL) in a sealed tube for 12 h. [b]5 mL of dioxane were used. [c]10 mL of dioxane were used. [d]20 mL of dioxane were used.

Preparation of chiral ligands

The chiral ligand **L2** was purchased and used without purification. **L3-L10** were prepared by reported methods[1-3]. **L11** was prepared from TADDOL **(*R*,*R*)-S1** by modified procedure.

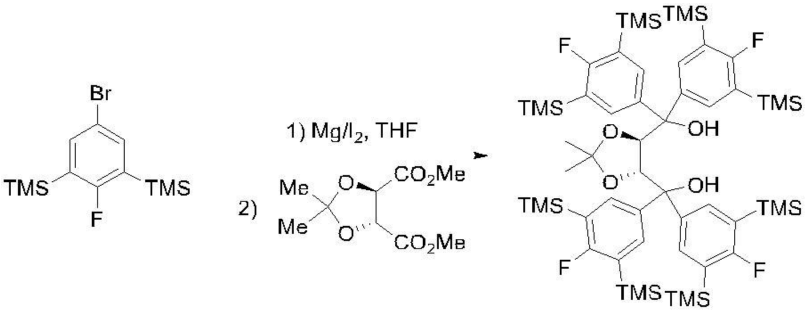

(*R*,*R*)-S1

A 250 ml single-necked, round-bottomed flask equipped with an egg-shaped magnetic stir bar and reflux condensation device was flame-dried under vacuum. Mg (1.1 g, 44.0 mmol) was added and the reaction flask was put under an atmosphere of N_2. A grain of I_2 and THF (80 mL) were added. (5-Bromo-2-fluoro-1,3-phenylene)bis-(trimethylsilane) (14.1 g, 44.0 mmol) was added slowly, then the reaction mixture was stirred at rt for 3 h. The dimethyl (4*R*,5*R*)-2,2-dimethyl-1,3-dioxolane-4,5-dicarboxylate (2.2 g, 10.0 mmol, in 30 mL THF) was added slowly, then the reaction was carried out for 12 hours at room temperature. The reaction mixture was quenched with saturated aqueous NH_4Cl. The phases were separated and the aqueous layer was extracted with Et_2O (3×100 mL). The combined organic extracts were washed with brine (150 mL), dried over anhydrous Na_2SO_2, filtered, and concentrated in vacuum. Flash chromatography (100:1 hexanes:ethyl acetate) afforded the **(*R*,*R*)-S1** (8.14 g, 73%) as a foamy solid. mp 85-86 ^{o}C. ^{1}H NMR (400 MHz, $CDCl_3$) δ 7.60 (d, *J* = 5.6 Hz, 4H), 7.37 (d, *J* = 5.6 Hz, 4H), 4.39 (s, 2H), 1.00 (s, 6H), 0.29 (s, 36H), 0.21 (s, 36H). ^{13}C NMR (100 MHz, CDCl3) δ 171.6 (d, *J* = 235.4 Hz), 171.4 (d, *J* = 236.3 Hz), 140.3 (d, *J* = 2.7 Hz), 136.7 (d, *J* = 11.9 Hz), 136.6 (d, *J* = 2.4 Hz), 136.0 (d, *J* = 12.2 Hz), 124.6 (d, *J* = 35.7 Hz), 123.8 (d, *J* = 35.5 Hz), 108.8, 81.9, 77.9, 27.0, -0.7, -0.9. IR：υ 2957， 1579， 1398， 1250， 1104， 839， 772， 694，622 cm^{-1}. HRMS (ESI-TOF) m/z: [M + Na]$^+$ Calcd for $C_{55}H_{90}F_4NaO_4Si_8$, 1137.4822; found 1137.4785.

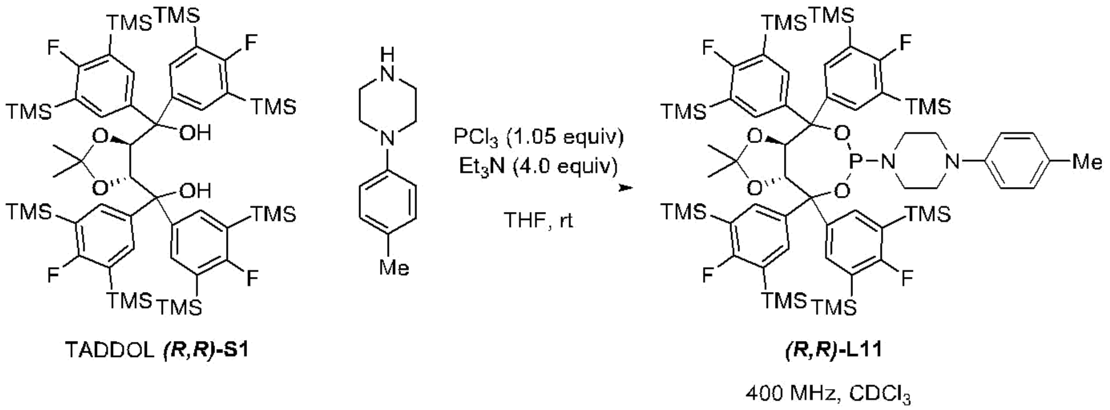

1-((3a*R*,8a*R*)-4,4,8,8-tetrakis(4-fluoro-3,5-bis(trimethylsilyl)phenyl)-2,2-dimethyltetrahydro-[1,3]dioxolo[4,5-*e*][1,3,2]dioxaphosphepin-6-yl)-4-(*p*-tolyl)piperazine (L11)

A 250 ml single-necked, round-bottomed flask equipped with an egg-shaped magnetic stir bar is flame-dried under vacuum. After cooling to 25 ℃, (*R,R*)-Taddol **S1** (3.5 g, 3.0 mmol) is added, the reaction flask is put under an atmosphere of N_2, and THF (100 mL) is added via syringe. To this clear, colorless solution, triethylamine (1.6 mL, 12 mmol) is added via syringe resulting in a clear solution with a slight yellow color. The reaction mixture is cooled to 0 ℃ with an ice bath and PCl_3 (0.3 mL, 3.15 mmol) is added dropwise over 2 min via syringe resulting in a white suspension. The ice bath is removed and the reaction is allowed to warm to 25 ℃ and stirred for 1 h. The reaction mixture is cooled to 0 ℃ with an ice bath and 1-(4-methylphenyl)-piperazine (1.55 g, 4.0 mmol) is added via syringe. The ice bath is removed and the reaction mixture is stirred at rt for 12 h, then filtered through a pad of celite. The celite was further washed with dry THF and the volatile solvents were removed. The residue was dried on a vacuum line. Flash chromatography (hexanes/ethyl acetate/Et_3N = 100:1:1) afforded the **L11** (3.37 g, 85%). White foamy solid. mp 82-84 ^oC. ^1H NMR (400 MHz, $CDCl_3$): δ 7.84 (d, J = 5.2 Hz, 2H), 7.81 (d, J = 5.2 Hz, 2H), 7.54 (d, J = 4.8 Hz, 2H), 7.49 (d, J = 5.2 Hz, 2H), 7.15 (d, J = 8.0 Hz, 2H), 6.93 (d, J = 8.0 Hz, 2H), 5.08 (d, J = 8.4 Hz, 1H), 4.52 (d, J = 8.4 Hz, 1H), 3.57 (s, 4H), 3.22 (s, 4H), 2.33 (s, 3H), 1.41 (s, 3H), 0.35-0.27 (m, 75H); ^{13}C NMR (100 MHz, $CDCl_3$): δ 171.6 (d, J = 235.8 Hz), 171.5 (d, J = 236.3 Hz), 171.23 (d, J = 235.4 Hz), 171.17 (d, J = 236.4 Hz), 149.8, 141.3 (d, J = 2.3 Hz), 140.9, 137.4 (d, J = 12.7 Hz), 137.22 (d, J = 10.6 Hz), 137.20 (d, J = 3.4 Hz), 137.0 (d, J = 3.5 Hz), 135.4 (d, J = 11.8

Hz), 129.9, 124.6 (d, *J* = 35.8 Hz), 124.3 (d, *J* = 35.0 Hz), 123.9 (d, *J* = 35.6 Hz), 123.5 (d, *J* = 36.0 Hz), 117.2, 110.6, 83.6, 83.4 (d, *J* = 17.1 Hz), 81.8 (d, *J* = 7.7 Hz), 81.2, 51.7 (d, *J* = 4.5 Hz), 44.2 (d, *J* = 18.4 Hz), 27.9, 24.5, 20.6, -0.4, -0.7, -0.8. IR: υ 2956, 2901, 1580, 1512, 1400, 1250, 1104, 1051, 840, 772, 694, 623 cm^{-1}. HRMS (ESI-TOF) m/z: $[M + H]^+$ Calcd for $C_{66}H_{104}F_4N_2O_4PSi_8$, 1319.5818; found 1319.5824.

Typical procedure for the synthesis of 1-indanones 2

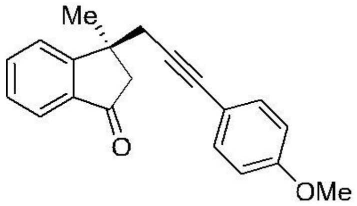

(*S*)-3-(3-(4-methoxyphenyl)prop-2-yn-1-yl)-3-methyl-2,3-dihydro-1*H*-inden-1-one (2a)

A vial was charged with cyclobutanone **1a** (57.2 mg, 0.2 mmol), $PdCl_2$ (1.8 mg, 5 mol%), CuI (1.9 mg, 5 mol%), **(*R*, *R*)-L11** (26.4 mg, 10 mol%), and 1-adamantanamine (151.2 mg, 5 equiv) and evacuated under high vacuum and backfilled with N_2. Dioxane (10 mL) and 4-methoxyphenylacetylene (31.7 mg, 0.24 mmol) were next added. The mixture was stirred for 0.5 h at room temperature and subsequently immersed in a preheated oil bath at 80 ℃. Upon reaction completion (12 h, TLC, eluent: hexane-EtOAc, 15:1), the mixture was filtered over a plug of silica gel (washed with 50 mL EtOAc), and the filtrate was concentrated. The mobile phase for flash chromatography: hexane/ethyl acetate = 15:1. Yellow oil. (50.0 mg, 86%). $[\alpha]^{25}_{D}$ = +70.1 (c = 0.45, $CHCl_3$). ^{1}H NMR (400 MHz, $CDCl_3$): δ 7.74 (d, *J* = 7.6 Hz, 1H), 7.67-7.57 (m, 2H), 7.41 (t, *J* = 7.2 Hz, 1H), 7.20 (d, *J* = 8.8 Hz, 2H), 6.78 (d, *J* = 8.8 Hz, 2H), 3.78 (s, 3H), 2.93 (d, *J* = 18.8 Hz, 1H), 2.77 (d, *J* = 16.8 Hz, 1H), 2.71 (d, *J* = 16.8 Hz, 1H), 2.57 (d, *J* = 18.8 Hz, 1H), 1.58 (s, 3H). ^{13}C NMR (100 MHz, $CDCl_3$): δ 205.1, 161.1, 159.5, 136.4, 135.0, 133.0, 128.1, 124.0, 123.4, 115.6, 114.0, 85.1, 82.9, 55.4, 50.9, 42.2, 33.7, 27.3. IR: υ 1702, 1607, 1511, 1463, 1438, 1327, 1289, 1244, 1178, 1087, 840, 753 cm^{-1}. HRMS (ESI-TOF) m/z: $[M + Na]^+$ Calcd for $C_{20}H_{18}NaO_2$, 313.1199; found 313.1209. Enantiomeric excess was determined by HPLC with a Chiralpak IC column (hexane: 2-propanol = 95:5, 1.0 mL/min, 254 nm, 96:4 *er*); major enantiomer t_r =23.1 min, minor enantiomer t_r =28.7 min.

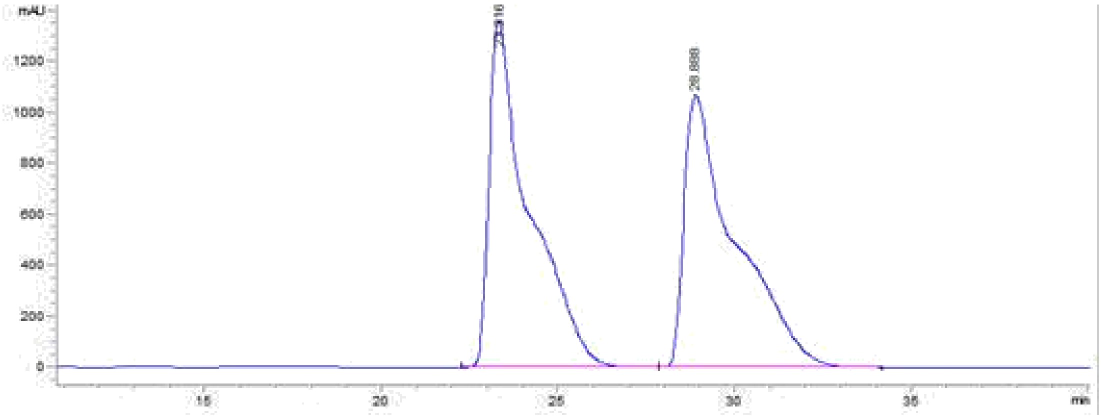

	Time/min	Area	Height	Area%
1	23.316	107102.8	1357	49.996
2	28.888	107119.6	1065.6	50.004

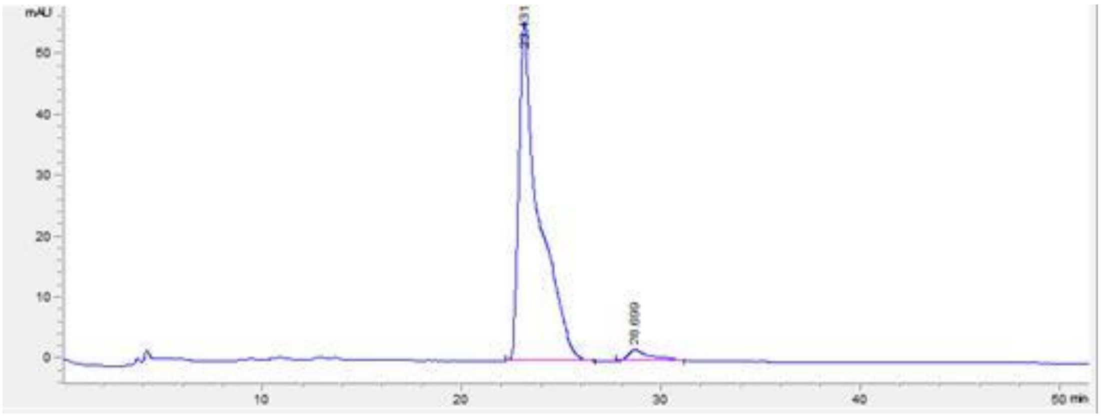

	Time/min	Area	Height	Area%
1	23.131	3890.6	55.4	96.164
2	28.699	155.2	1.9	3.836

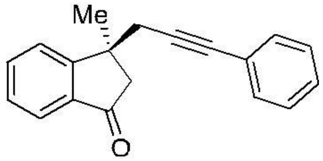

(*S*)-3-methyl-3-(3-phenylprop-2-yn-1-yl)-2,3-dihydro-1*H*-inden-1-one (2b)

The mobile phase for flash chromatography: hexane/ethyl acetate = 15:1. Yellow oil. (48.9 mg, 94%). $[\alpha]^{25}_{D}$ = +2.7 (c = 0.52, $CHCl_3$). ^1H NMR (400 MHz, $CDCl_3$): δ 7.67 (d, *J* = 7.6 Hz, 1H), 7.59-7.50 (m, 2H), 7.34 (t, *J* = 7.2 Hz, 1H), 7.20-7.15 (m, 5H),

2.86 (d, J = 18.8 Hz, 1H), 2.72 (d, J = 16.8 Hz, 1H), 2.66 (d, J = 16.8 Hz, 1H), 2.51 (d, J = 18.8 Hz, 1H), 1.52 (s, 3H); ^{13}C NMR (100 MHz, $CDCl_3$): δ 205.0, 160.9, 136.3, 135.0, 131.6, 128.3, 128.1, 128.0, 123.9, 123.4, 123.3, 86.6, 83.0, 50.8, 42.1, 33.6, 27.3. IR: υ 1709, 1602, 1489, 1463, 1374, 1260, 1070, 901, 843, 753, 690 cm^{-1}. HRMS (ESI-TOF) m/z: $[M + Na]^+$ Calcd for $C_{19}H_{16}NaO$, 283.1093; found 283.1103. Enantiomeric excess was determined by HPLC with a Chiralpak OD column (hexane: 2-propanol = 95:5, 1.0 mL/min, 254 nm, 97.5:2.5 *er*); major enantiomer t_r = 9.2 min, minor enantiomer t_r = 10.3 min.

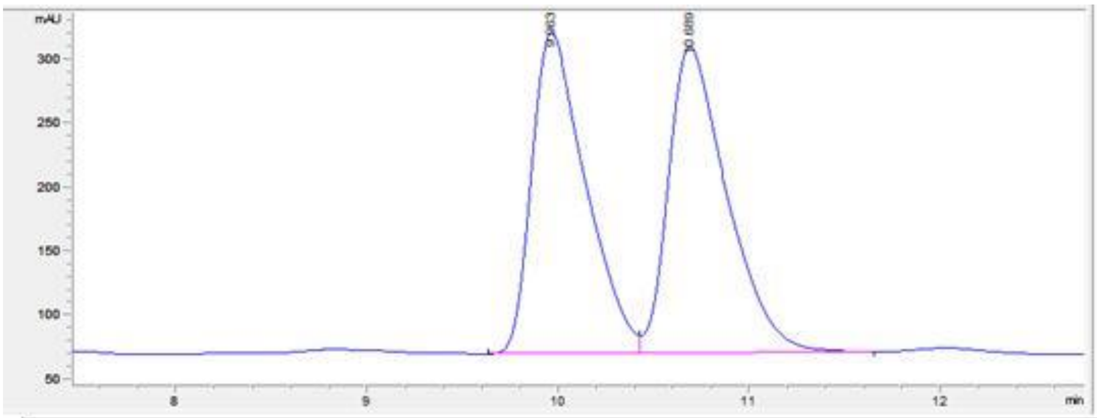

	Time/min	Area	Height	Area%
1	9.963	4935	251.4	49.195
2	10.689	5096	240.1	50.805

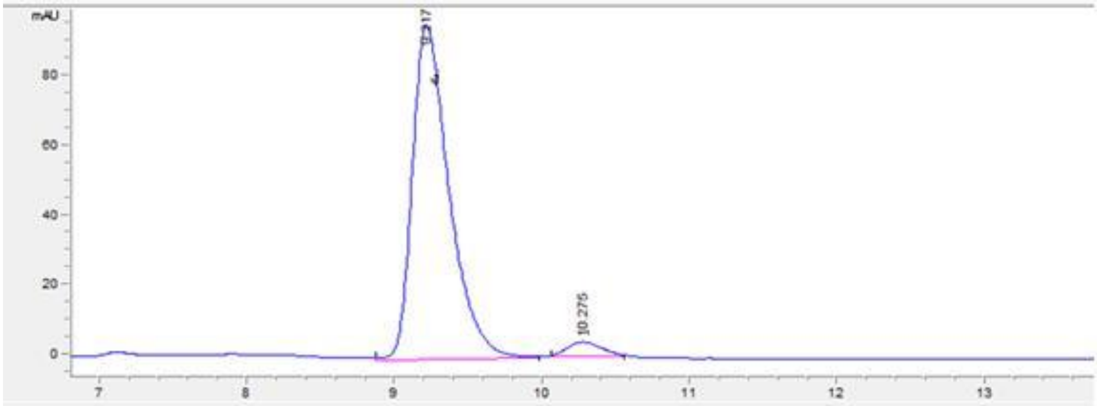

	Time/min	Area	Height	Area%
1	9.217	1701.4	96.8	97.400
2	10.275	46.1	3.2	2.600

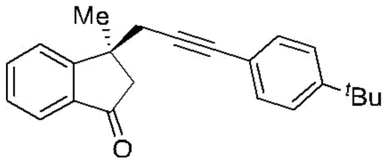

(*S*)-3-(3-(4-(tert-butyl)phenyl)prop-2-yn-1-yl)-3-methyl-2,3-dihydro-1*H*-inden-1-one (2c)

The mobile phase for flash chromatography: hexane/ethyl acetate = 15:1. Yellow oil. (57.5 mg, 91%). $[\alpha]^{25}_{D}$ = -54.0 (c = 0.61, $CHCl_3$). ^{1}H NMR (400 MHz, $CDCl_3$): δ 7.67 (d, J = 7.6 Hz, 1H), 7.59-7.52 (m, 2H), 7.33 (t, J = 7.2 Hz, 1H), 7.20 (d, J = 8.0 Hz, 2H), 7.13 (d, J = 8.0 Hz, 2H), 2.85 (d, J = 18.8 Hz, 1H), 2.71 (d, J = 16.8 Hz, 1H), 2.65 (d, J = 16.8 Hz, 1H), 2.50 (d, J = 18.8 Hz, 1H), 1.51 (s, 3H), 1.21 (s, 9H); ^{13}C NMR (100 MHz, $CDCl_3$): δ 205.2, 161.1, 151.2, 136.4, 135.0, 131.3, 128.1, 125.3, 124.0, 123.4, 120.3, 85.9, 83.1, 50.8, 42.1, 34.8, 33.6, 31.3, 27.3. IR: υ 2957, 2870, 1715, 1604, 1510, 1464, 1377, 1286, 1170, 1073, 961, 834, 753, 679 cm^{-1}. HRMS (ESI-TOF) m/z: $[M + Na]^+$ Calcd for $C_{23}H_{24}NaO$, 339.1719; found 339.1732. Enantiomeric excess was determined by HPLC with a Chiralpak OD column (hexane: 2-propanol = 95:5, 1.0 mL/min, 254 nm, 93:7 *er*); major enantiomer t_r =6.3 min, minor enantiomer t_r =7.0 min.

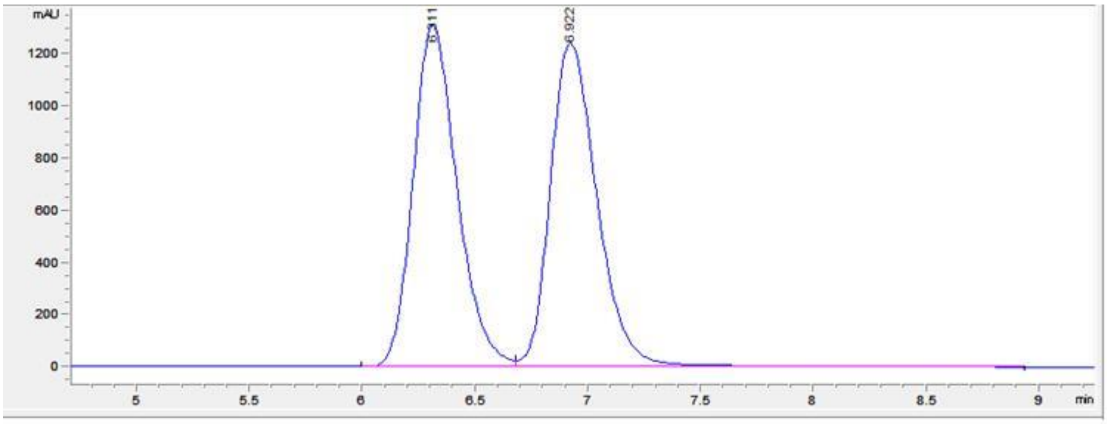

	Time/min	Area	Height	Area%
1	6.311	17781.6	1313.4	49.717
2	6.922	17984.2	1242.7	50.283

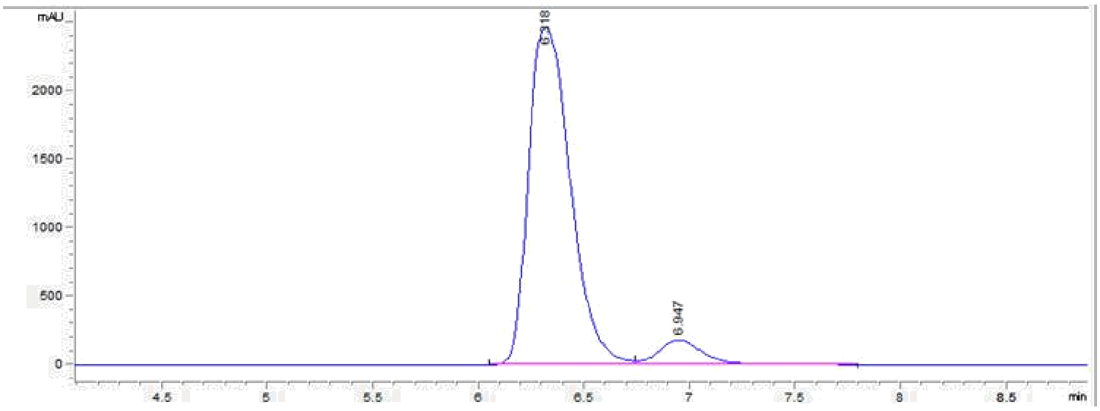

	Time/min	Area	Height	Area%
1	6.318	34091	2471	92.785
2	6.947	2650.8	179.9	7.215

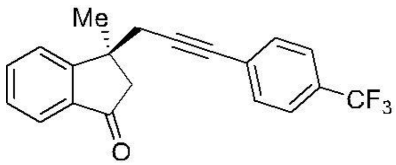

(*S*)-3-methyl-3-(3-(4-(trifluoromethyl)phenyl)prop-2-yn-1-yl)-2,3-dihydro-1*H*-inden-1-one (2d)

The mobile phase for flash chromatography: hexane/ethyl acetate = 15:1. Yellow oil. (58.8 mg, 89%). $[\alpha]^{25}_{D}$ = +21.5 (c = 0.79, $CHCl_3$). ^{1}H NMR (400 MHz, $CDCl_3$): δ 7.75 (d, *J* = 7.6 Hz, 1H), 7.65 (t, *J* = 7.2 Hz, 1H), 7.59 (d, *J* = 8.0 Hz, 1H), 7.50 (d, *J* = 8.0 Hz, 2H), 7.42 (t, *J* = 7.2 Hz, 1H), 7.33 (d, *J* = 8.0 Hz, 2H), 2.92 (d, *J* = 18.8 Hz, 1H), 2.82 (d, *J* = 16.8 Hz, 1H), 2.76 (d, *J* = 16.8 Hz, 1H), 2.60 (d, *J* = 18.8 Hz, 1H), 1.60 (s, 3H); ^{13}C NMR (100 MHz, $CDCl_3$): δ 204.9, 160.6, 136.4, 135.1, 131.9, 129.8 (q, *J* = 32.0 Hz), 128.3, 127.1, 125.3 (q, *J* = 4.0 Hz), 124.0 (q, *J* = 271.0 Hz), 123.8, 123.5, 89.4, 82.0, 50.8, 42.1, 33.7, 27.3. IR: υ 1716, 1615, 1604, 1405, 1464, 1324, 1290, 1166, 1125, 1067, 1031, 1017, 843, 763, 754 cm^{-1}. HRMS (ESI-TOF) m/z: $[M + Na]^+$ Calcd for $C_{20}H_{15}F_3NaO$, 351.0967; found 351.0983. Enantiomeric excess was determined by HPLC with a Chiralpak OD column (hexane: 2-propanol = 99.5:0.5, 1.0 mL/min, 254 nm, 95.5:4.5 *er*); major enantiomer t_r =36.8 min, minor enantiomer t_r =41.8 min.

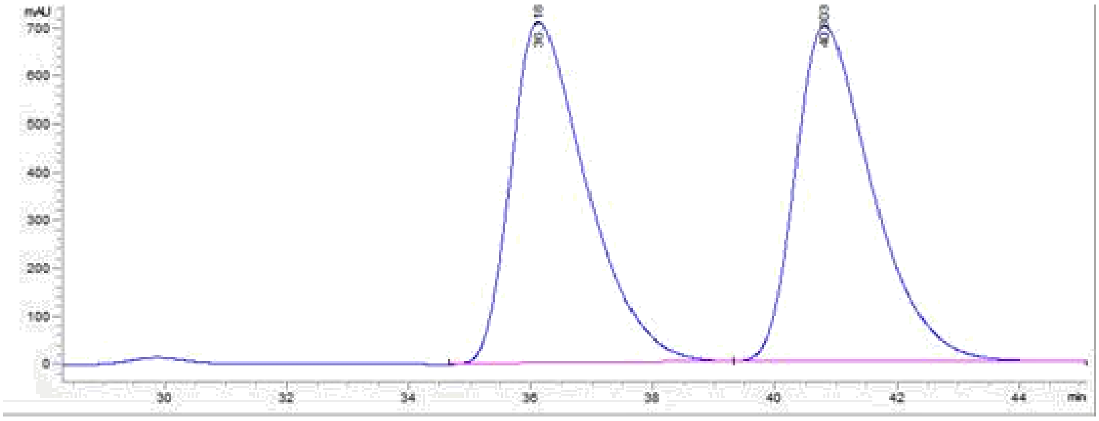

	Time/min	Area	Height	Area%
1	36.118	61385.5	711.4	50.015
2	40.803	61349.7	698.6	49.985

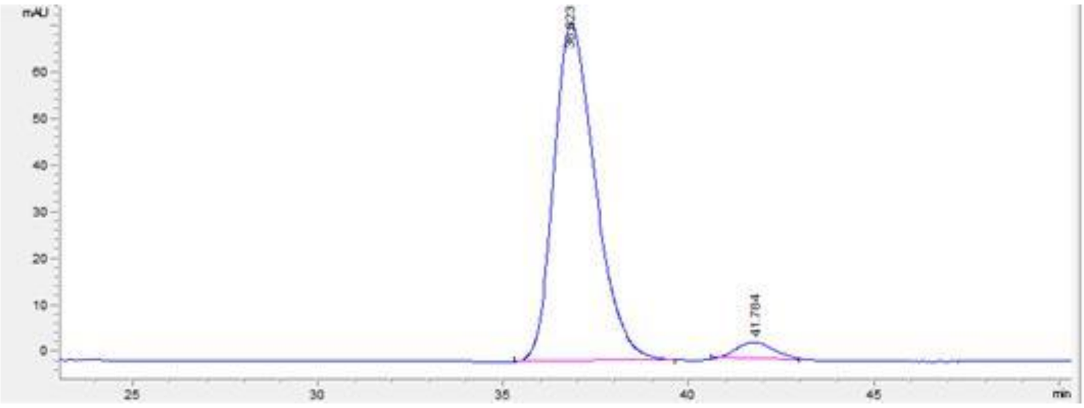

	Time/min	Area	Height	Area%
1	36.823	5610.1	72.6	95.711
2	41.784	251.4	3.5	4.289

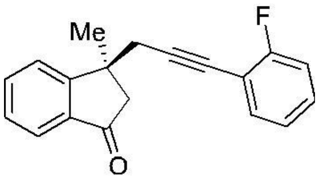

(*S*)-3-(3-(2-fluorophenyl)prop-2-yn-1-yl)-3-methyl-2,3-dihydro-1*H*-inden-1-one (2e)

The mobile phase for flash chromatography: hexane/ethyl acetate = 15:1. Yellow oil. (47.4 mg, 85%). $[\alpha]^{25}_{D}$ = -131.8 (c = 0.77, $CHCl_3$). ^{1}H NMR (400 MHz, $CDCl_3$): δ 7.67 (d, *J* = 7.6 Hz, 1H), 7.59-7.52 (m, 2H), 7.38-7.30 (m, 1H), 7.22-7.13 (m, 2H),

6.98-6.90 (m, 2H), 2.85 (d, *J* = 18.8 Hz, 1H), 2.75 (d, *J* = 16.8 Hz, 1H), 2.70 (d, *J* = 16.8 Hz, 1H), 2.52 (d, *J* = 18.8 Hz, 1H), 1.52 (s, 3H). ^{13}C NMR (100 MHz, $CDCl_3$): δ 205.1, 163.0 (d, *J* = 249.0 Hz), 160.3, 136.3, 135.1, 133.5, 129.7 (d, *J* = 8.0 Hz), 128.2, 124.1, 124.0 (d, *J* = 4.0 Hz), 123.5, 115.0 (d, *J* = 20.0 Hz), 111.8 (d, *J* = 15.0 Hz), 92.2 (d, *J* = 3.0 Hz), 76.4, 50.8, 42.0, 33.7, 27.4. IR: υ 1715, 1645, 1603, 1491, 1463, 1377, 1289, 1257, 1102, 1031, 858, 755, 699 cm^{-1}. HRMS (ESI-TOF) m/z: [M + Na]$^{+}$ Calcd for $C_{19}H_{15}FNaO$, 301.0999; found 301.1009. Enantiomeric excess was determined by HPLC with a Chiralpak OD column (hexane: 2-propanol = 99:1, 1.0 mL/min, 254 nm, 94:6 *er*); major enantiomer t_r =15.0 min, minor enantiomer t_r =16.5 min.

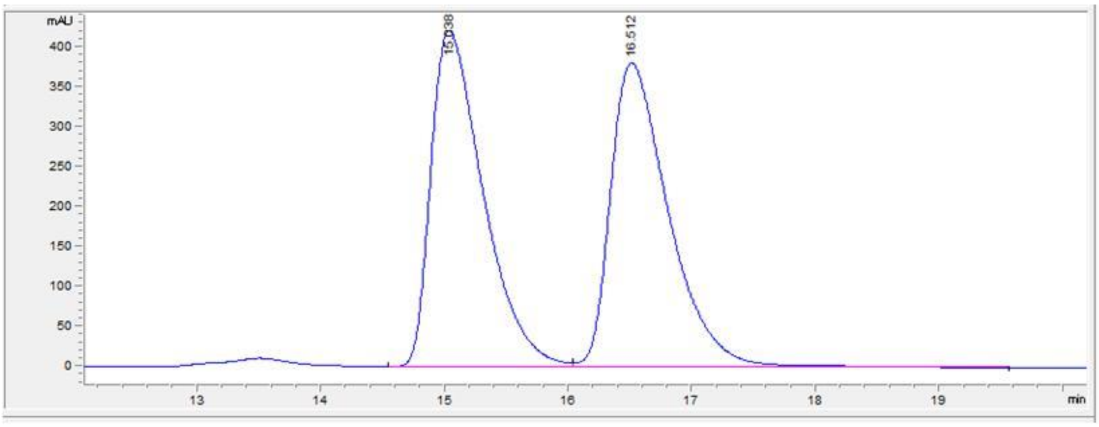

	Time/min	Area	Height	Area%
1	15.038	12396.5	420.8	49.813
2	16.512	12489.7	381.2	50.187

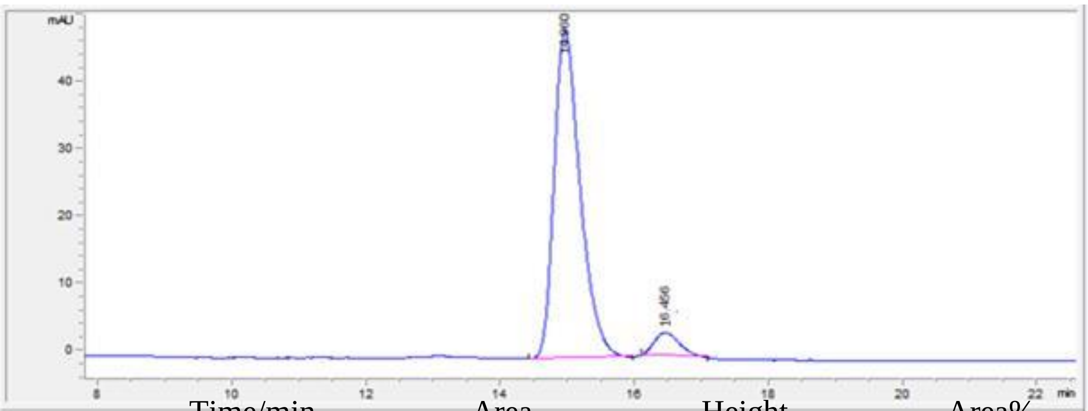

	Time/min	Area	Height	Area%

1	14.96	1320.1	48.8	94.051
2	16.456	83.5	3.3	5.949

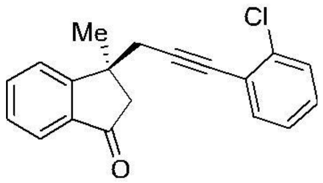

(*S*)-3-(3-(2-chlorophenyl)prop-2-yn-1-yl)-3-methyl-2,3-dihydro-1*H*-inden-1-one (2f)

The mobile phase for flash chromatography: hexane/ethyl acetate = 15:1. Yellow oil. (47.7 mg, 81%). $[\alpha]^{25}_{D}$ = -39.6 (c = 0.72, $CHCl_3$). ^1H NMR (400 MHz, $CDCl_3$): δ 7.74 (d, *J* = 7.6 Hz, 1H), 7.67-7.60 (m, 2H), 7.44-7.38 (m, 1H), 7.35-7.28 (m, 2H), 7.20-7.12 (m, 2H), 2.97 (d, *J* = 18.8 Hz, 1H), 2.86 (d, *J* = 16.8 Hz, 1H), 2.80 (d, *J* = 16.8 Hz, 1H), 2.61 (d, *J* = 18.8 Hz, 1H), 1.60 (s, 3H); ^{13}C NMR (100 MHz, $CDCl_3$): δ 205.1, 161.0, 136.3, 136.0, 135.1, 133.4, 129.3, 129.0, 128.2, 126.5, 124.0, 123.6, 123.2, 92.3, 79.8, 50.8, 42.1, 33.6, 27.6. IR: υ 2957, 2923, 1716, 1646, 1603, 1463, 1377, 1260, 1093, 1019, 800, 753 cm^{-1}. HRMS (ESI-TOF) m/z: $[M + Na]^+$ Calcd for $C_{19}H_{15}ClNaO$, 317.0704; found 317.0717. Enantiomeric excess was determined by HPLC with a Chiralpak OD column (hexane: 2-propanol = 98:2, 1.0 mL/min, 254 nm, 92:8 *er*); major enantiomer t_r =16.2 min, minor enantiomer t_r =19.6 min.

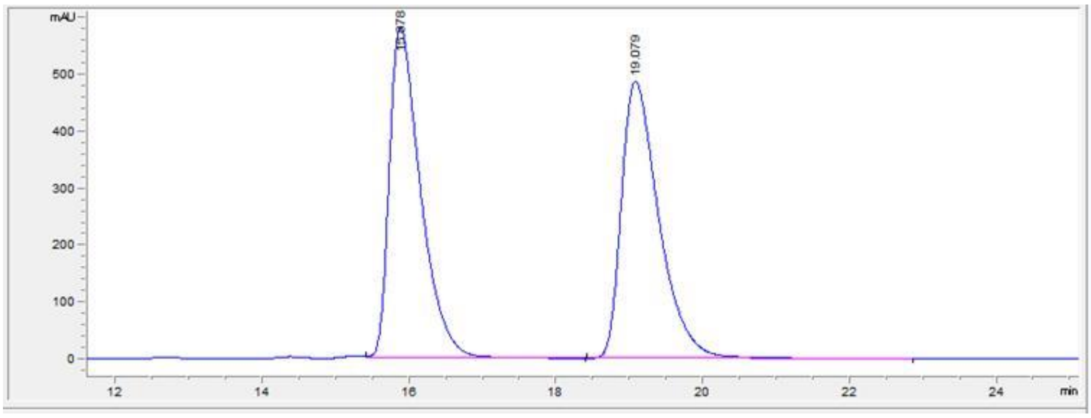

	Time/min	Area	Height	Area%
1	15.878	56344.8	598.2	49.976
2	19.079	56632.6	519.8	50.024

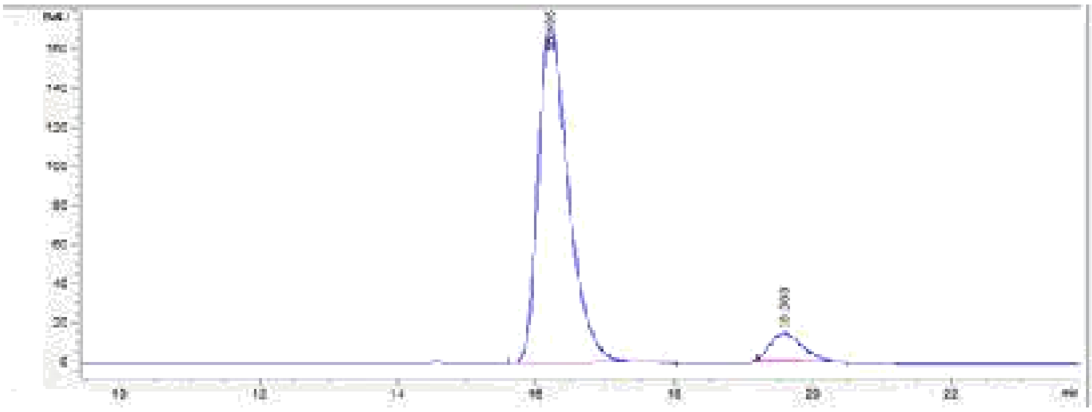

	Time/min	Area	Height	Area%
1	16.195	5252.5	171.9	91.603
2	19.583	481.5	14.3	8.397

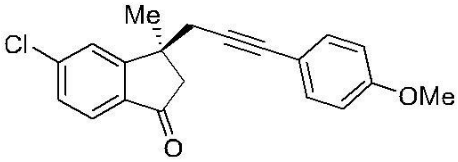

(*S*)-5-chloro-3-(3-(4-methoxyphenyl)prop-2-yn-1-yl)-3-methyl-2,3-dihydro-1*H*-inden-1-one (2g)

The mobile phase for flash chromatography: hexane/ethyl acetate = 10:1. Yellow oil. (62.3 mg, 96%). $[\alpha]^{25}_{D}$ = -40.9 (c = 0.58, $CHCl_3$). ^{1}H NMR (400 MHz, $CDCl_3$): δ 7.66 (d, *J* = 8.0 Hz, 1H), 7.59 (d, *J* = 4.0 Hz, 1H), 7.38 (dd, *J* = 8.0, 4.0 Hz, 1H), 7.21 (d, *J* = 8.0 Hz, 2H), 6.79 (d, *J* = 8.0 Hz, 2H), 3.78 (s, 3H), 2.92 (d, *J* = 18.8 Hz, 1H), 2.75 (d, *J* = 16.8 Hz, 1H), 2.70 (d, *J* = 16.8 Hz, 1H), 2.58 (d, *J* = 18.8 Hz, 1H), 1.57 (s, 3H); ^{13}C NMR (100 MHz, $CDCl_3$): δ 203.6, 162.4, 159.5, 141.4, 134.9, 133.0, 128.9, 124.63, 124.57, 115.3, 114.0, 84.6, 83.3, 55.4, 50.8, 42.2, 33.6, 27.1. IR: υ 1712, 1610, 1696, 1486, 1458, 1376, 1262, 1181, 1080, 1020, 898, 823, 750 cm^{-1}. HRMS (ESI-TOF) m/z: $[M + Na]^+$ Calcd for $C_{20}H_{17}ClNaO_2$, 347.0809; found 347.0819. Enantiomeric excess was determined by HPLC with a Chiralpak OD column (hexane: 2-propanol = 99:1, 1.0 mL/min, 254 nm, 96:4 *er*); major enantiomer t_r =30.4 min, minor enantiomer t_r =35.1 min.

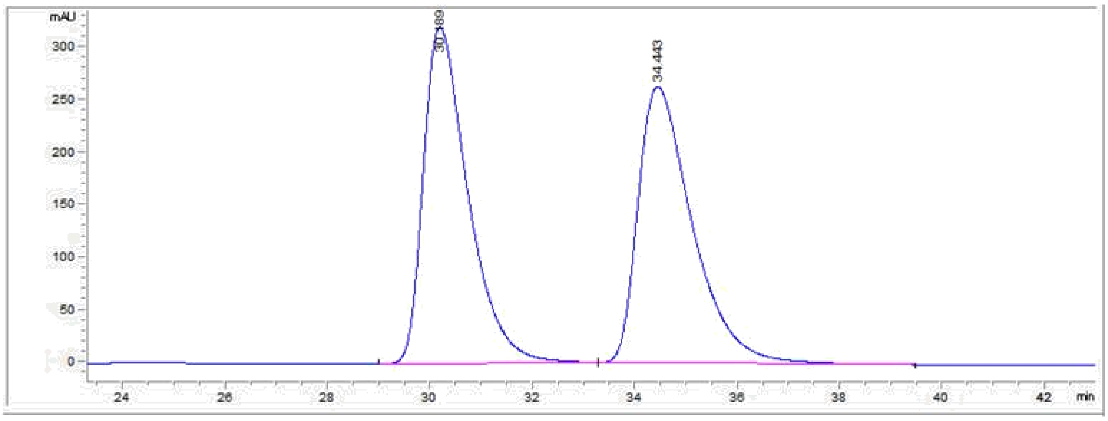

	Time/min	Area	Height	Area%
1	30.189	19398.9	320.5	50.040
2	34.443	19368.1	262.8	49.960

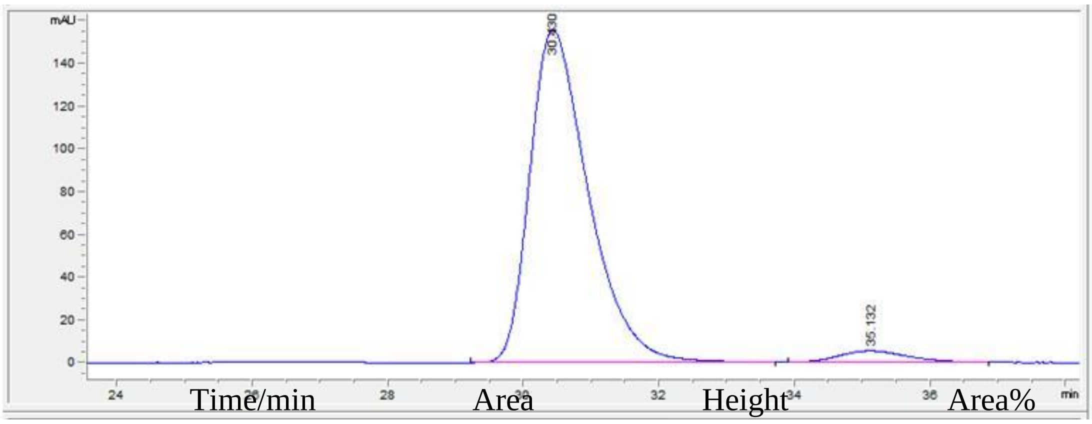

	Time/min	Area	Height	Area%
1	30.43	9336.8	155.4	96.206
2	35.132	368.2	5.3	3.794

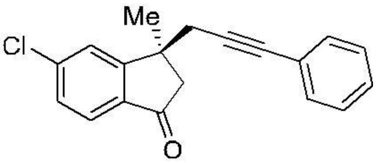

(*S*)-5-chloro-3-methyl-3-(3-phenylprop-2-yn-1-yl)-2,3-dihydro-1*H*-inden-1-one (2h)

The mobile phase for flash chromatography: hexane/ethyl acetate = 15:1. Yellow oil. (56.2 mg, 95%). $[\alpha]^{25}_{D}$ = +3.1 (c = 0.73, $CHCl_3$). ^{1}H NMR (400 MHz, $CDCl_3$): δ 7.59 (d, *J* = 7.6 Hz, 1H), 7.52 (d, *J* = 1.6 Hz, 1H), 7.31 (dd, *J* = 7.6, 1.6 Hz, 1H), 7.21-7.16

(m, 5H), 2.85 (d, *J* = 18.8 Hz, 1H), 2.70 (d, *J* = 16.8 Hz, 1H), 2.65 (d, *J* = 16.8 Hz, 1H), 2.52 (d, *J* = 18.8 Hz, 1H), 1.50 (s, 3H); ^{13}C NMR (100 MHz, $CDCl_3$): δ 203.5, 162.3, 141.4, 134.9, 131.6, 128.9, 128.4, 128.1, 124.7, 124.5, 123.1, 86.1, 83.5, 50.8, 42.1, 33.6, 27.1. IR: υ 1716, 1597, 1489, 1458, 1377, 1310, 1259, 1068, 1028, 880, 759, 692 cm^{-1}. HRMS (ESI-TOF) m/z: $[M + Na]^+$ Calcd for $C_{19}H_{15}ClNaO$, 317.0704; found 317.0713. Enantiomeric excess was determined by HPLC with a Chiralpak OD column (hexane: 2-propanol = 99:1, 1.0 mL/min, 254 nm, 96:4 *er*); major enantiomer t_r =16.2 min, minor enantiomer t_r =18.6 min.

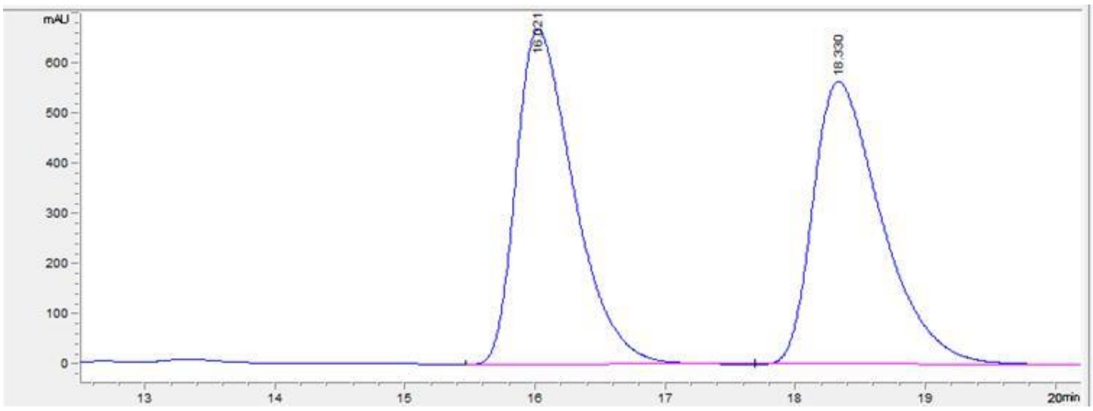

	Time/min	Area	Height	Area%
1	16.021	20745.8	669.5	49.988
2	18.33	20755.7	565.3	50.012

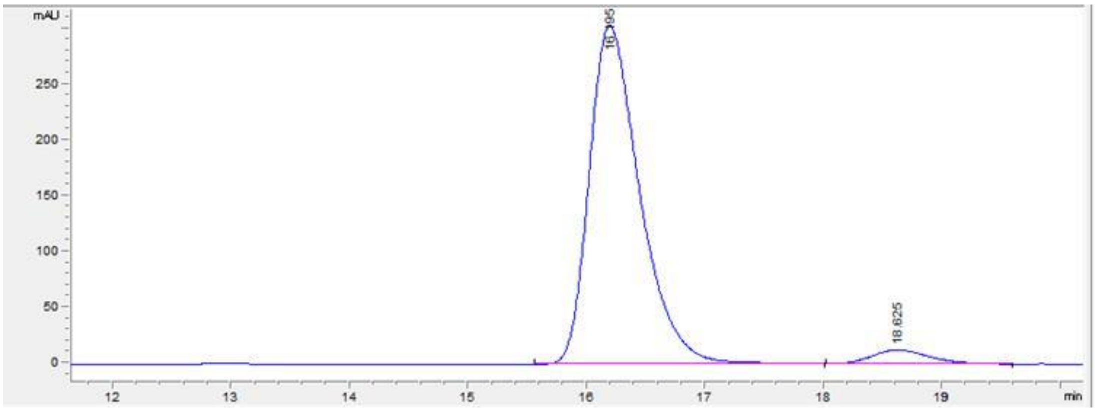

	Time/min	Area	Height	Area%
1	16.195	8996.6	303.6	95.621
2	18.625	412	12.4	4.379

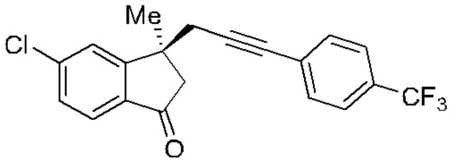

(*S*)-5-chloro-3-methyl-3-(3-(4-(trifluoromethyl)phenyl)prop-2-yn-1-yl)-2,3-dihydro-1*H*-inden-1-one (2i)

The mobile phase for flash chromatography: hexane/ethyl acetate = 10:1. Yellow oil. (68.0 mg, 94%). $[\alpha]^{25}_{D}$ = +88.8 (c = 0.7, $CHCl_3$). ^{1}H NMR (400 MHz, $CDCl_3$): δ 7.67 (d, *J* = 8.0 Hz, 1H), 7.58 (d, *J* = 1.6 Hz, 1H), 7.51 (d, *J* = 8.0 Hz, 2H), 7.39 (dd, *J* = 8.0, 1.6 Hz, 1H), 7.35 (d, *J* = 8.0 Hz, 2H), 2.91 (d, *J* = 18.8 Hz, 1H), 2.80 (d, *J* = 16.8 Hz, 1H), 2.75 (d, *J* = 16.8 Hz, 1H), 2.60 (d, *J* = 18.8 Hz, 1H), 1.59 (s, 3H). ^{13}C NMR (100 MHz, $CDCl_3$): δ 203.3, 162.0, 141.6, 135.0, 131.9, 129.9 (q, *J* = 32.5 Hz), 129.1, 126.9, 125.3 (q, *J* = 3.7 Hz), 124.7, 124.4, 124.0 (q, *J* = 270.5 Hz), 88.9, 82.3, 50.8, 42.1, 33.6, 27.1. IR: υ 1719, 1597, 1460, 1377, 1323, 1260, 1167, 1128, 1067, 1108, 842, 803 cm^{-1}. HRMS (ESI-TOF) m/z: $[M + Na]^+$ Calcd for $C_{20}H_{14}ClF_3NaO$, 385.0577; found 385.0587. Enantiomeric excess was determined by HPLC with a Chiralpak OD column (hexane: 2-propanol = 99:1, 1.0 mL/min, 254 nm, 93.5:6.5 *er*); major enantiomer t_r =14.0 min, minor enantiomer t_r =15.2 min.

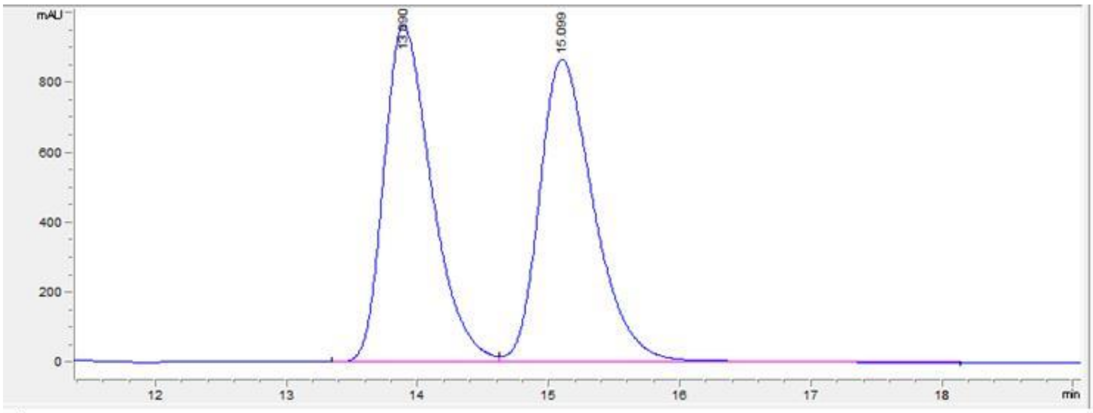

	Time/min	Area	Height	Area%
1	13.89	24383.9	963.1	49.329
2	15.099	25047.4	865.7	50.671

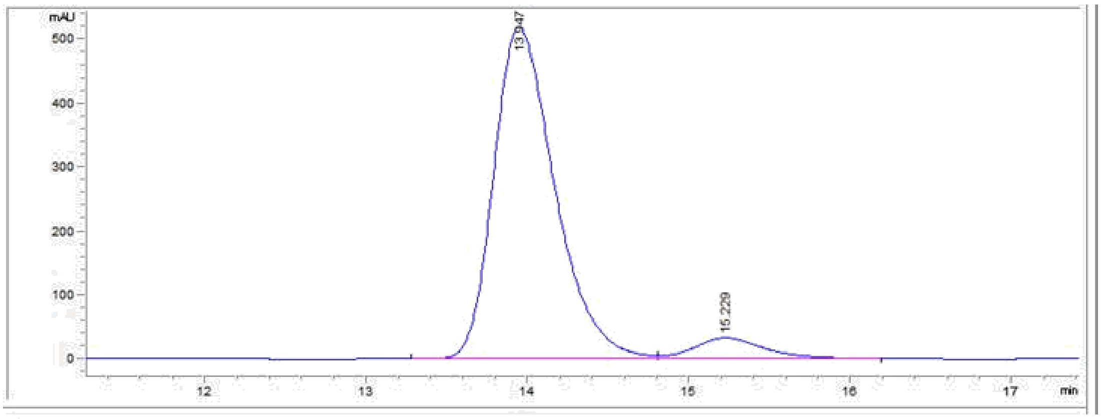

	Time/min	Area	Height	Area%
1	13.947	13575.7	518.3	93.370
2	15.229	963.9	31.8	6.630

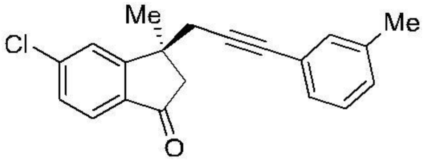

(*S*)-5-chloro-3-methyl-3-(3-(m-tolyl)prop-2-yn-1-yl)-2,3-dihydro-1*H*-inden-1-one (2j)

The mobile phase for flash chromatography: hexane/ethyl acetate = 15:1. Yellow oil. (59.6 mg, 96%). $[\alpha]^{25}_{D}$ = +6.6 (c = 0.93, $CHCl_3$). ^{1}H NMR (400 MHz, $CDCl_3$): δ 7.67 (d, J = 8.0 Hz, 1H), 7.61 (d, J = 1.6 Hz, 1H), 7.39 (dd, J = 8.0, 1.6 Hz, 1H), 7.17-7.05 (m, 4H), 2.92 (d, J = 18.8 Hz, 1H), 2.77 (d, J = 16.8 Hz, 1H), 2.72 (d, J = 16.8 Hz, 1H), 2.59 (d, J = 18.8 Hz, 1H), 2.30 (s, 3H), 1.57 (s, 3H); ^{13}C NMR (100 MHz, $CDCl_3$): δ 203.5, 162.4, 141.4, 138.0, 134.8, 132.2, 129.0, 128.9, 128.7, 128.3, 124.7, 124.6, 122.9, 85.7, 83.6, 50.8, 42.1, 33.5, 27.1, 21.3. IR: υ 1708, 1597, 1483, 1457, 1378, 1313, 1251, 1217, 1101, 1067, 896, 831, 788, 692 cm^{-1}. HRMS (ESI-TOF) m/z: $[M + Na]^+$ Calcd for $C_{20}H_{17}ClNaO$, 331.0860; found 331.0870. Enantiomeric excess was determined by HPLC with a Chiralpak OD column (hexane: 2-propanol = 99:1, 1.0 mL/min, 254 nm, 93:7 *er*); major enantiomer t_r =15.7 min, minor enantiomer t_r =18.1 min.

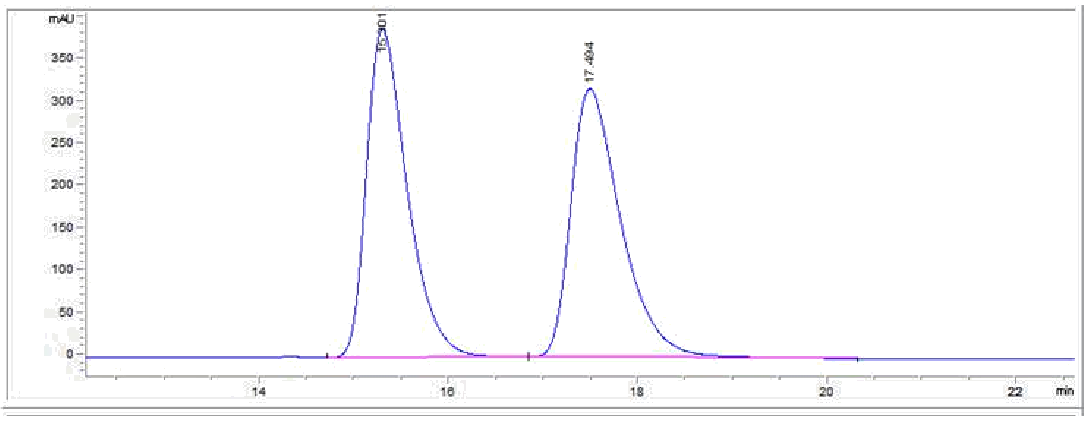

	Time/min	Area	Height	Area%
1	15.301	11605.3	388.8	49.946
2	17.494	11630.3	317.6	50.054

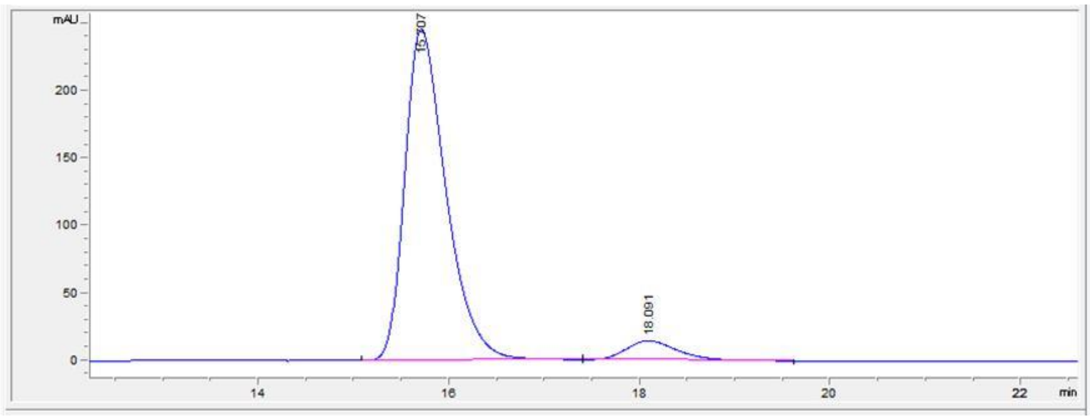

	Time/min	Area	Height	Area%
1	15.707	7419.3	245.6	93.288
2	18.091	533.8	14.3	6.712

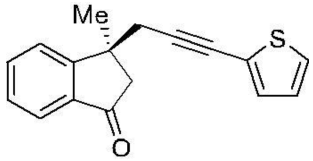

(*S*)-3-methyl-3-(3-(thiophen-2-yl)prop-2-yn-1-yl)-2,3-dihydro-1*H*-inden-1-one (2k)

The mobile phase for flash chromatography: hexane/ethyl acetate = 15:1. Yellow oil. (48.1 mg, 90%). $[\alpha]^{25}_{D}$ = -174.6 (c = 0.51, $CHCl_3$). ^{1}H NMR (400 MHz, $CDCl_3$): δ 7.74 (d, *J* = 7.6 Hz, 1H), 7.69-7.55 (m, 2H), 7.42 (t, *J* = 7.2 Hz, 1H), 7.17 (d, *J* = 5.2

Hz, 1H), 7.05 (d, *J* = 3.2 Hz, 1H), 6.91 (dd, *J* = 5.2, 3.2 Hz, 1H), 2.90 (d, *J* = 18.8 Hz, 1H), 2.81 (d, *J* = 16.8 Hz, 1H), 2.75 (d, *J* = 16.8 Hz, 1H), 2.59 (d, *J* = 18.8 Hz, 1H), 1.58 (s, 3H); ^{13}C NMR (100 MHz, $CDCl_3$): δ 205.0, 160.9, 136.3, 135.1, 131.6, 128.2, 126.9, 126.6, 124.0, 123.6, 123.4, 90.8, 76.3, 50.8, 42.1, 33.9, 27.3. IR: υ 2923, 1716, 1644, 1494, 1463, 1377, 1260, 1187, 1081, 967 cm^{-1}. HRMS (ESI-TOF) m/z: $[M + Na]^+$ Calcd for $C_{17}H_{14}NaOS$, 289.0658; found 289.0669. Enantiomeric excess was determined by HPLC with a Chiralpak OD column (hexane: 2-propanol = 99:1, 1.0 mL/min, 254 nm, 93.5:6.5 *er*); major enantiomer t_r =18.2 min, minor enantiomer t_r =21.3 min.

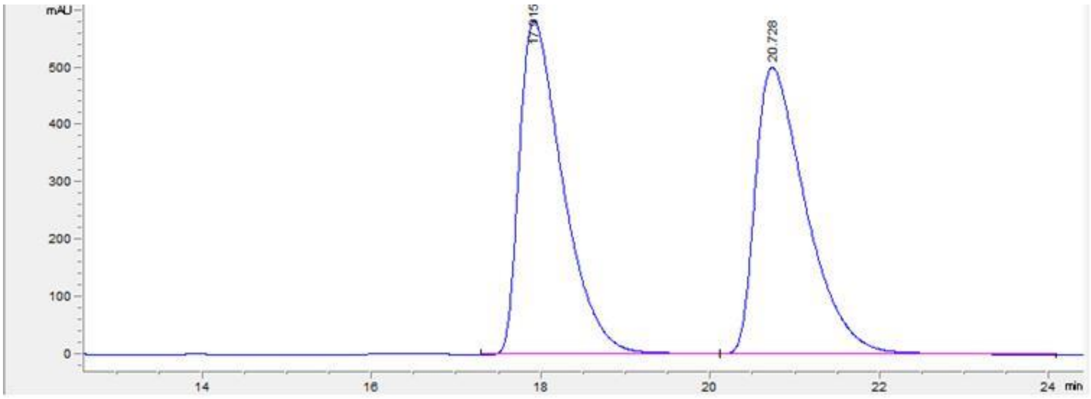

	Time/min	Area	Height	Area%
1	17.915	20970.9	584.2	49.978
2	20.728	20989.3	501.9	50.022

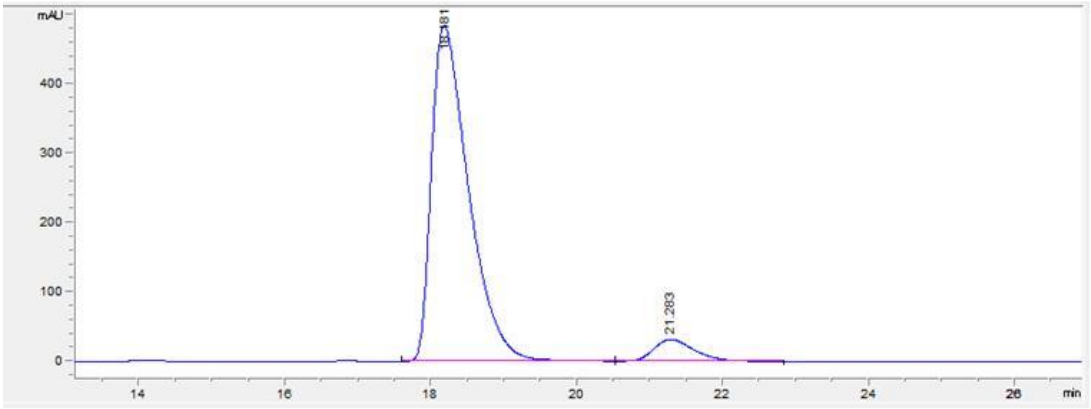

	Time/min	Area	Height	Area%
1	18.181	17124.5	485	93.374
2	21.283	1215.3	31.8	6.626

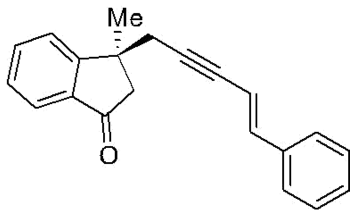

(S,E)-3-methyl-3-(5-phenylpent-4-en-2-yn-1-yl)-2,3-dihydro-1*H*-inden-1-one (2l)

The mobile phase for flash chromatography: hexane/ethyl acetate = 15:1. Yellow oil. (52.1 mg, 91%). $[\alpha]^{25}_{D}$ = +4.2 (c = 0.51, $CHCl_3$). ^{1}H NMR (400 MHz, $CDCl_3$): δ 7.75 (d, J = 7.6 Hz, 1H), 7.65 (t, J = 7.6 Hz, 1H), 7.59 (d, J = 7.6 Hz, 1H), 7.42 (t, J = 7.2 Hz, 1H), 7.35-7.25 (m, 5H), 6.78 (d, J = 16.4 Hz, 1H), 6.05 (d, J = 16.4 Hz, 1H), 2.90 (d, J = 18.8 Hz, 1H), 2.76 (d, J = 16.8 Hz, 1H), 2.70 (d, J = 16.8 Hz, 1H), 2.57 (d, J = 18.8 Hz, 1H), 1.57 (s, 3H). ^{13}C NMR (100 MHz, $CDCl_3$): δ 205.1, 161.0, 141.0, 136.4, 136.3, 135.1, 128.8, 128.6, 128.1, 126.2, 123.9, 123.5, 108.2, 89.0, 82.2, 50.8, 42.1, 33.8, 27.4. IR: υ 1714, 1599, 1494, 1463, 1376, 1290, 1214, 1173, 1030, 1011, 991, 884, 754, 691 cm^{-1}. HRMS (ESI-TOF) m/z: $[M + Na]^{+}$ Calcd for $C_{21}H_{18}NaO$, 309.1250; found 309.1252. Enantiomeric excess was determined by HPLC with a Chiralpak OD column (hexane: 2-propanol = 95:5, 1.0 mL/min, 254 nm, 88.5:11.5 *er*); major enantiomer t_r = 35.9 min, minor enantiomer t_r = 27.4 min.

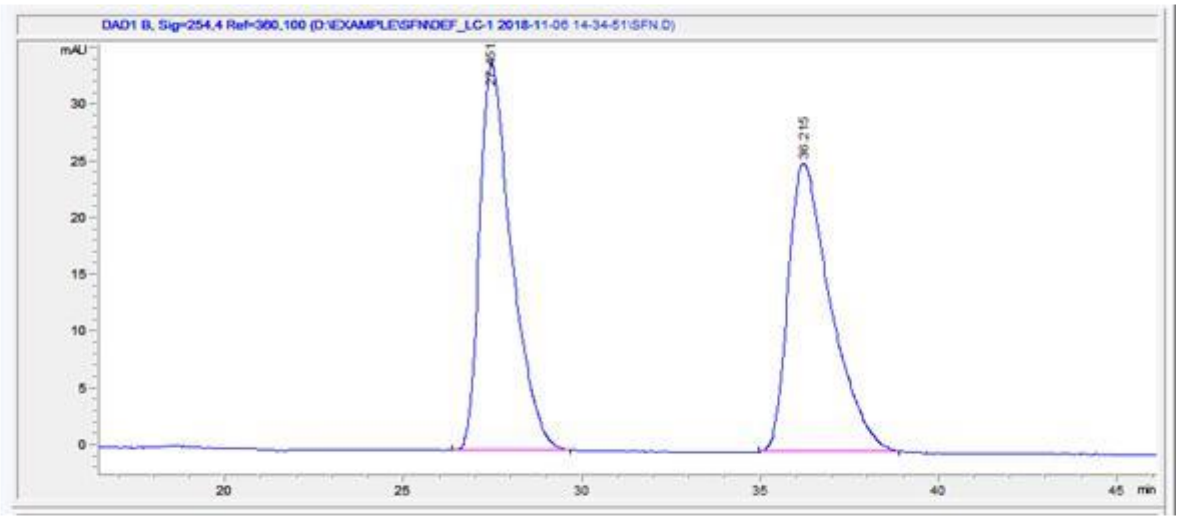

	Time/min	Area	Height	Area%
1	27.451	2124.6	34.2	50.185
2	36.215	2109	25.4	49.815

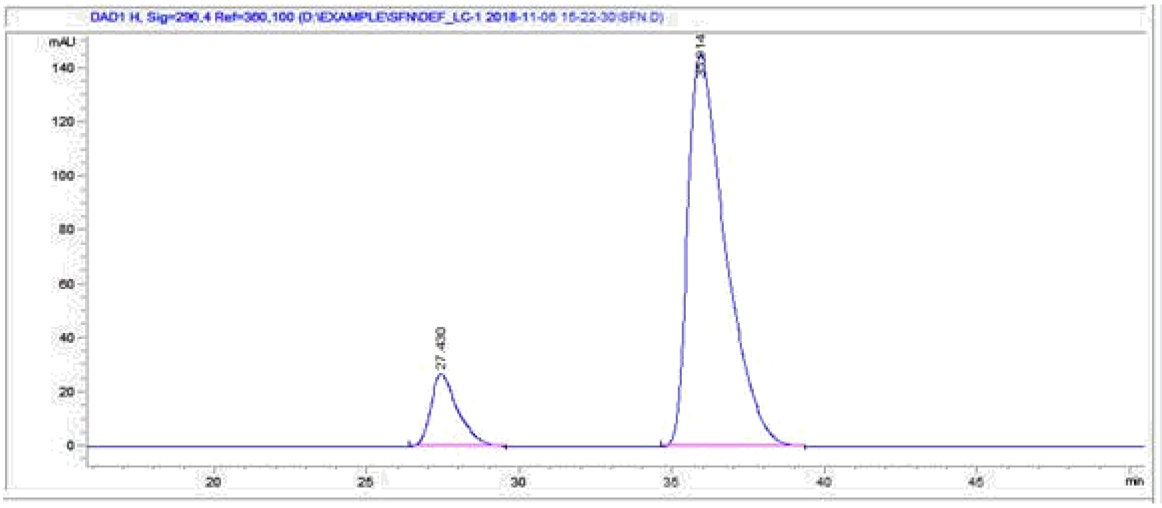

	Time/min	Area	Height	Area%
1	27.43	1642.3	26.5	11.652
2	35.91	12451.9	145.1	88.348

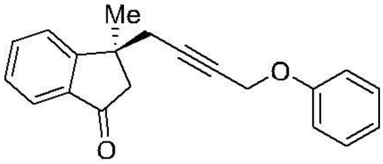

(*S*)-3-methyl-3-(4-phenoxybut-2-yn-1-yl)-2,3-dihydro-1*H*-inden-1-one (2m)

The mobile phase for flash chromatography: hexane/ethyl acetate = 15:1. Yellow oil. (50.6 mg, 87%). $[\alpha]^{25}_{D}$ = +55.2 (c = 0.54, $CHCl_3$). ^{1}H NMR (400 MHz, $CDCl_3$): δ 7.62 (d, *J* = 7.6 Hz, 1H), 7.49 (t, *J* = 7.6 Hz, 1H), 7.40 (d, *J* = 7.6 Hz, 1H), 7.30 (t, *J* = 7.6 Hz, 1H), 7.19 (t, *J* = 7.6 Hz, 2H), 6.89 (t, *J* = 7.6 Hz, 1H), 6.79 (d, *J* = 7.6 Hz, 2H), 4.54 (d, *J* = 16.8 Hz, 1H), 4.47 (d, *J* = 16.8 Hz, 1H), 2.72 (d, *J* = 18.8 Hz, 1H), 2.53 (d, *J* = 16.8 Hz, 1H), 2.48 (d, *J* = 16.8 Hz, 1H), 2.42 (d, *J* = 18.8 Hz, 1H), 1.39 (s, 3H). ^{13}C NMR (100 MHz, $CDCl_3$): δ 204.9, 160.7, 157.6, 136.1, 135.0, 129.5, 128.1, 123.9, 123.5, 121.4, 114.9, 84.6, 77.6, 56.0, 50.6, 41.7, 32.7, 27.4. IR: υ 1713, 1609, 1482, 1454, 1434, 1312, 1282, 1210, 1108, 1019, 870, 823, 734 cm^{-1}. HRMS (ESI-TOF) m/z: $[M + Na]^+$ Calcd for $C_{20}H_{18}NaO_2$, 313.1199; found 313.1205. Enantiomeric excess was determined by HPLC with a Chiralpak OD column (hexane: 2-propanol = 95:5, 1.0 mL/min, 254 nm, 95.5:4.5 *er*); major enantiomer t_r = 65.5 min, minor enantiomer t_r = 60.7 min.

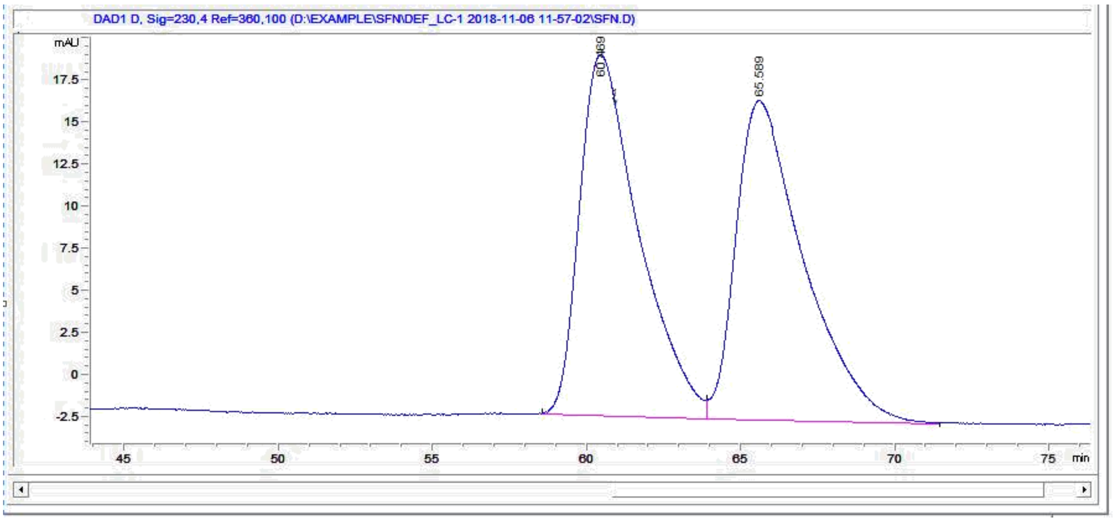

	Time/min	Area	Height	Area%
1	60.469	2974.3	21.5	49.411
2	65.589	2942.8	19	50.589

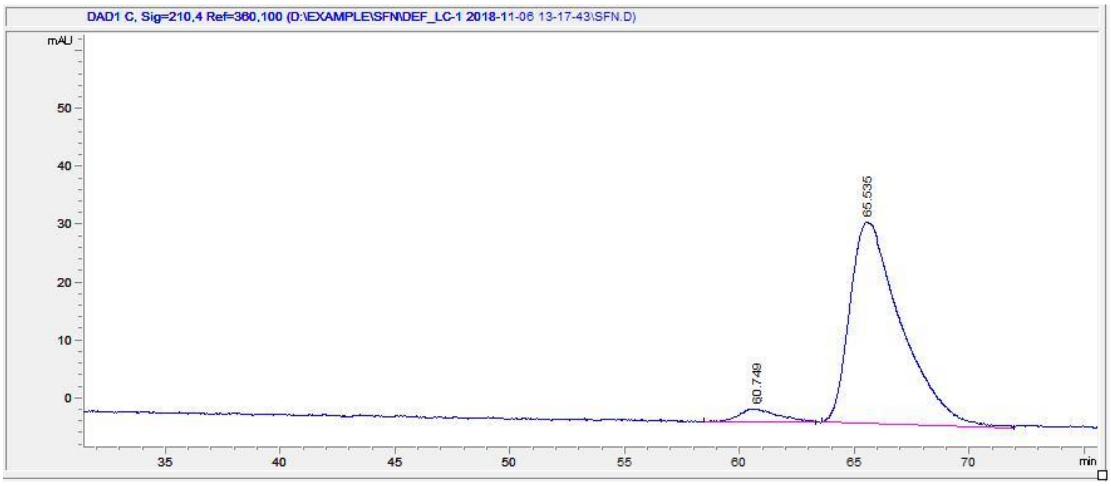

	Time/min	Area	Height	Area%
1	60.749	261.4	2.2	4.565
2	65.535	5466	35	95.436

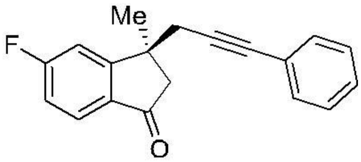

(*S*)-5-fluoro-3-methyl-3-(3-phenylprop-2-yn-1-yl)-2,3-dihydro-1*H*-inden-1-one (2n)

The mobile phase for flash chromatography: hexane/ethyl acetate = 15:1. Yellow oil. (51.8 mg, 93%). $[\alpha]^{25}_{D}$ = +6.1 (c = 0.47, $CHCl_3$). ^1H NMR (400 MHz, $CDCl_3$): δ 7.75 (dd, *J* = 8.4, 5.2 Hz, 1H), 7.30-7.22 (m, 6H), 7.10 (td, *J* = 8.4, 0.2 Hz, 1H), 2.94 (d, *J* = 18.8 Hz, 1H), 2.78 (d, *J* = 16.8 Hz, 1H), 2.73 (d, *J* = 16.8 Hz, 1H), 2.61 (d, *J* = 18.8 Hz, 1H), 1.58 (s, 3H). ^{13}C NMR (100 MHz, $CDCl_3$): δ 203.1, 167.4 (d, *J* = 256.4 Hz), 163.9 (d, *J* = 9.0 Hz), 132.8 (d, *J* = 2.0 Hz), 131.6, 128.4, 128.1, 125.9 (d, *J* = 10.0 Hz), 123.2, 116.4 (d, *J* = 24.0 Hz), 110.9 (d, *J* = 22.0 Hz), 86.1, 83.4, 50.9, 42.1, 33.5, 27.2. IR: υ 1712, 1612, 1590, 1488, 1478, 1332, 1298, 1267, 1244, 1067, 935, 825, 755, 691 cm^{-1}. HRMS (ESI-TOF) m/z: $[M + Na]^+$ Calcd for $C_{19}H_{15}FNaO$, 301.0999; found 301.1010. Enantiomeric excess was determined by HPLC with a Chiralpak OD column (hexane: 2-propanol = 99:1, 1.0 mL/min, 254 nm, 93:7 *er*); major enantiomer t_r =17.5 min, minor enantiomer t_r =19.8 min.

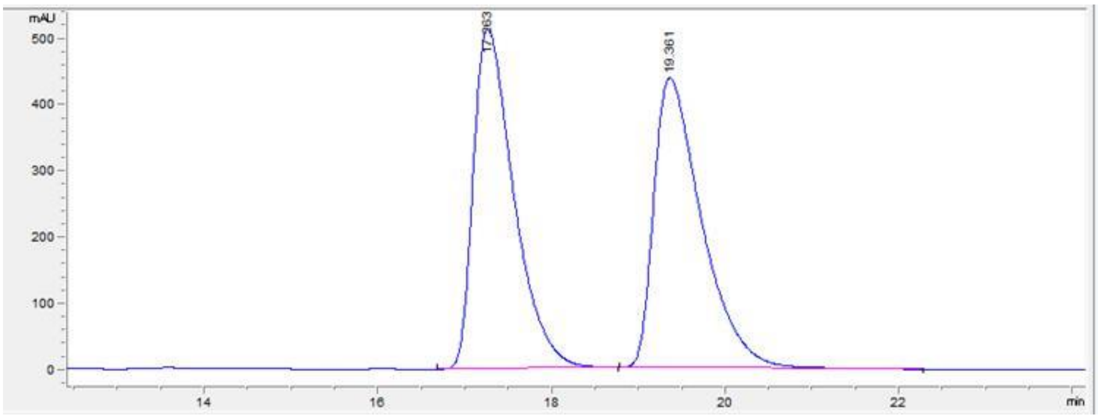

	Time/min	Area	Height	Area%
1	17.263	16626.8	513.7	49.813
2	19.361	16751.4	437.1	50.187

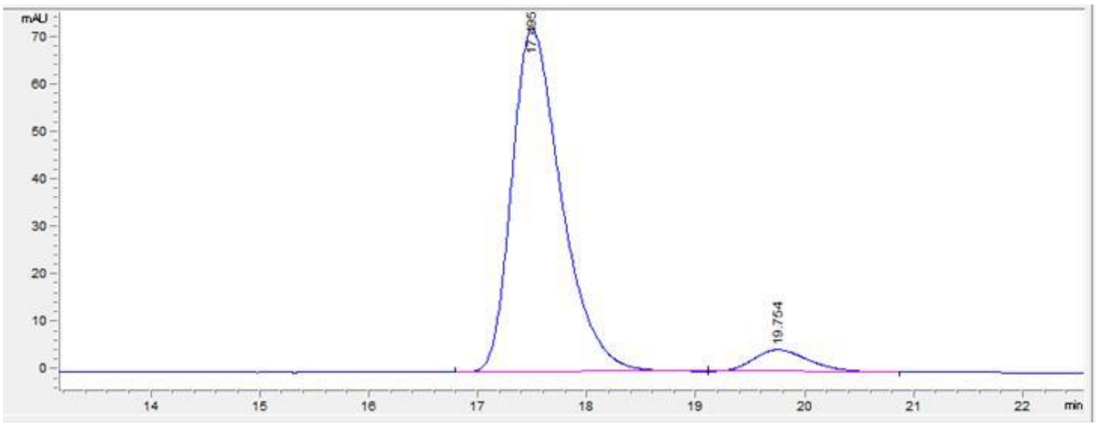

	Time/min	Area	Height	Area%
1	17.495	2289.5	72.3	93.257
2	19.754	165.5	4.6	6.743

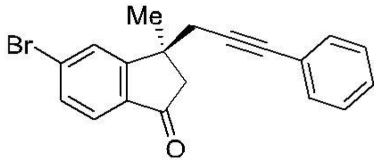

(*S*)-5-bromo-3-methyl-3-(3-phenylprop-2-yn-1-yl)-2,3-dihydro-1*H*-inden-1-one (2o)

The mobile phase for flash chromatography: hexane/ethyl acetate = 15:1. Yellow oil. (60.0 mg, 94%). $[\alpha]^{25}_{D}$ = -241.7 (c = 0.37, $CHCl_3$). ^{1}H NMR (400 MHz, $CDCl_3$): δ 7.78 (s, 1H), 7.60 (d, *J* = 8.0 Hz, 1H), 7.54 (d, *J* = 8.0 Hz, 1H), 7.29-7.20 (m, 5H), 2.91 (d, *J* = 18.8 Hz, 1H), 2.77 (d, *J* = 16.8 Hz, 1H), 2.72 (d, *J* = 16.8 Hz, 1H), 2.57 (d, *J* = 18.8 Hz, 1H), 1.57 (s, 3H); ^{13}C NMR (100 MHz, $CDCl_3$): δ 203.6, 162.4, 135.2, 131.8, 131.6, 130.2, 128.4, 128.1, 127.6, 124.8, 123.1, 86.1, 83.5, 50.7, 42.0, 33.6, 27.1. IR: υ 2923, 2851, 1716, 1592, 1457, 1377, 1080 cm^{-1}. HRMS (ESI-TOF) m/z: $[M + Na]^+$ Calcd for $C_{19}H_{15}BrNaO$, 361.0198; found 361.0207. Enantiomeric excess was determined by HPLC with a Chiralpak OD column (hexane: 2-propanol = 95:5, 1.0 mL/min, 254 nm, 93:7 *er*); major enantiomer t_r =10.2 min, minor enantiomer t_r =11.2 min.

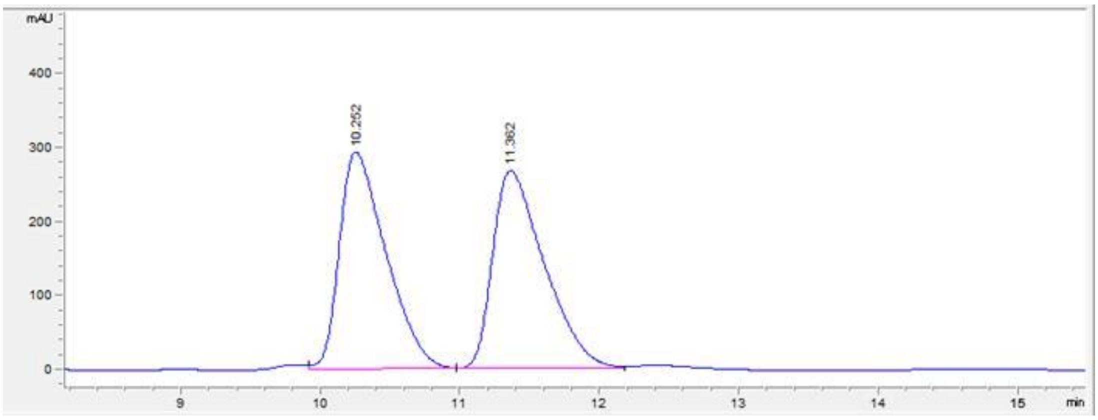

	Time/min	Area	Height	Area%
1	10.252	6802.7	293.7	49.600
2	11.362	6912.3	268	50.400

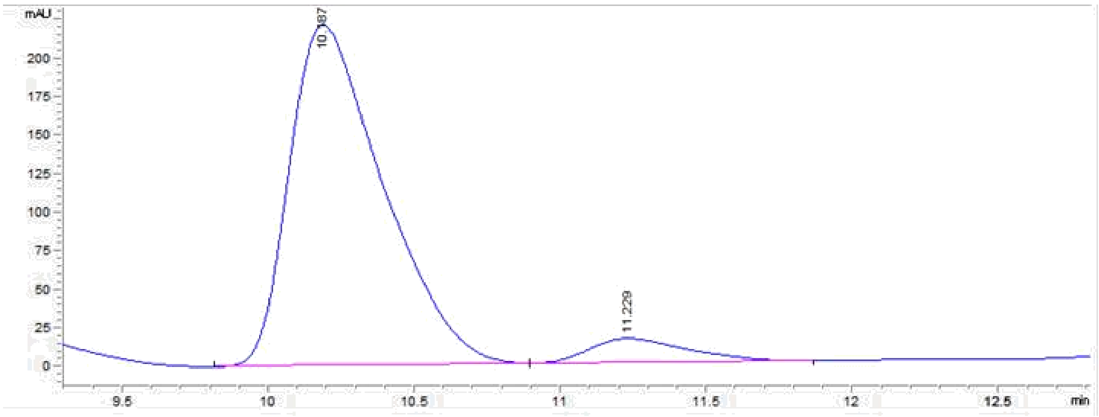

	Time/min	Area	Height	Area%
1	10.187	4924.9	221.3	93.096
2	11.229	365.2	15.7	6.904

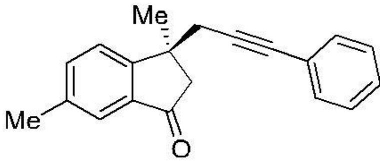

(*S*)-3,6-dimethyl-3-(3-phenylprop-2-yn-1-yl)-2,3-dihydro-1*H*-inden-1-one (2p)

The mobile phase for flash chromatography: hexane/ethyl acetate = 15:1. Yellow oil. (53.0 mg, 94%). $[\alpha]^{25}_{D}$ = +16.3 (c = 0.94, $CHCl_3$). ^{1}H NMR (400 MHz, $CDCl_3$): δ 7.46 (s, 1H), 7.41 (d, *J* = 8.0 Hz, 1H), 7.37 (d, *J* = 8.0 Hz, 1H), 7.23-7.15 (m, 5H), 2.84 (d, *J* = 18.8 Hz, 1H), 2.69 (d, *J* = 16.8 Hz, 1H), 2.63 (d, *J* = 16.8 Hz, 1H), 2.50 (d, *J* = 18.8 Hz, 1H). 2.33 (s, 3H), 1.49 (s, 3H); ^{13}C NMR (100 MHz, $CDCl_3$): δ 205.1, 158.5, 138.1, 136.5, 136.2, 131.6, 128.3, 128.0, 123.7, 123.48, 123.41, 86.9, 83.0, 51.1, 41.8, 33.7, 27.4, 21.2. IR: υ 1712, 1614, 1489, 1442, 1376, 1282, 1161, 1070, 825, 756, 691 cm^{-1}. HRMS (ESI-TOF) m/z: $[M + Na]^+$ Calcd for $C_{20}H_{18}NaO$, 297.1250; found 297.1260. Enantiomeric excess was determined by HPLC with a Chiralpak OD column (hexane: 2-propanol = 95:5, 1.0 mL/min, 254 nm, 92.5:7.5 *er*); major enantiomer t_r =7.7 min, minor enantiomer t_r =9.4 min.

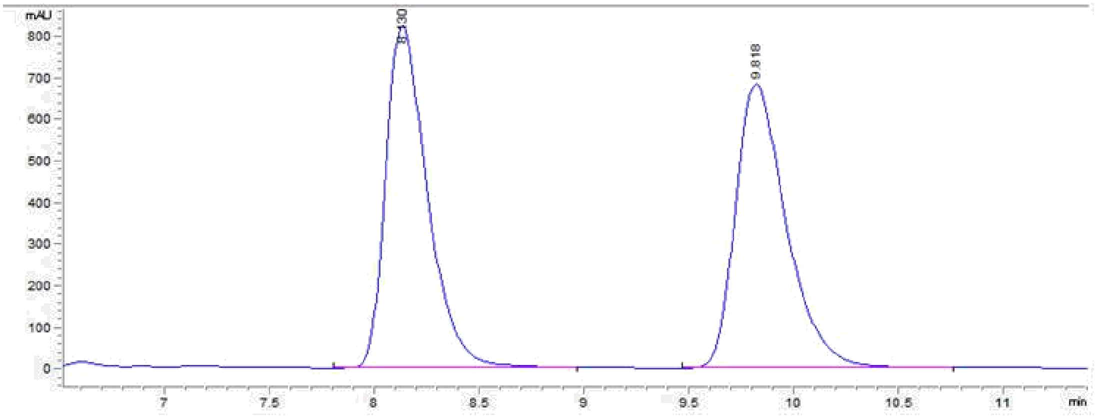

	Time/min	Area	Height	Area%
1	8.13	11361.4	822.2	49.745
2	9.818	11477.6	682.9	50.255

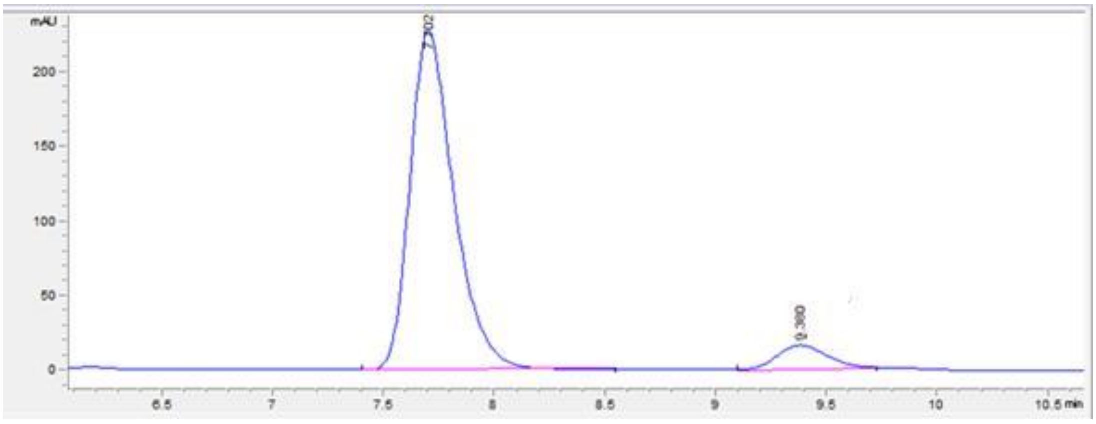

	Time/min	Area	Height	Area%
1	7.702	3139	226.9	92.341
2	9.38	260.4	1.63	7.659

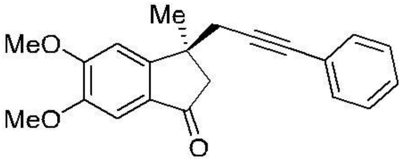

(*S*)-5,6-dimethoxy-3-methyl-3-(3-phenylprop-2-yn-1-yl)-2,3-dihydro-1*H*-inden-1-one (2q)

The mobile phase for flash chromatography: hexane/ethyl acetate = 10:1. Yellow oil. (59.1 mg, 92%). $[\alpha]^{25}_{D}$ = -52.0 (c = 1.13, $CHCl_3$). ^1H NMR (400 MHz, $CDCl_3$): δ 7.32-7.23 (m, 5H), 7.15 (s, 1H), 7.02 (s, 1H), 3.94 (s, 3H), 3.90 (s, 3H), 2.86 (d, J =

18.8 Hz, 1H), 2.74 (s, 2H), 2.56 (d, J = 18.8 Hz, 1H), 1.57 (s, 3H). ^{13}C NMR (100 MHz, $CDCl_3$): δ 203.4, 156.2, 155.6, 150.0, 131.6, 129.2, 128.4, 128.0, 123.4, 105.1, 104.0, 86.9, 83.1, 56.3, 56.2, 51.0, 41.6, 33.6, 27.2. IR: υ 1693, 1593, 1500, 1465, 1379, 1298, 1214, 1161, 1128, 1034, 952, 816, 757, 692 cm^{-1}. HRMS (ESI-TOF) m/z: $[M + Na]^+$ Calcd for $C_{21}H_{20}NaO_3$, 343.1305; found 343.1315. Enantiomeric excess was determined by HPLC with a Chiralpak OD column (hexane: 2-propanol = 99:1, 1.0 mL/min, 254 nm, 93:7 *er*); major enantiomer t_r =51.5 min, minor enantiomer t_r =62.4 min.

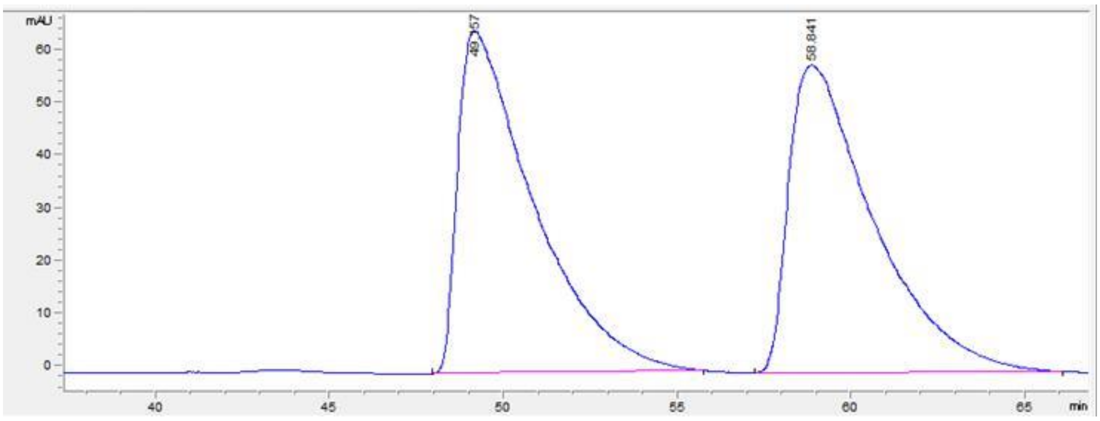

	Time/min	Area	Height	Area%
1	49.157	9834.7	64.9	49.895
2	58.841	9876	58.3	50.105

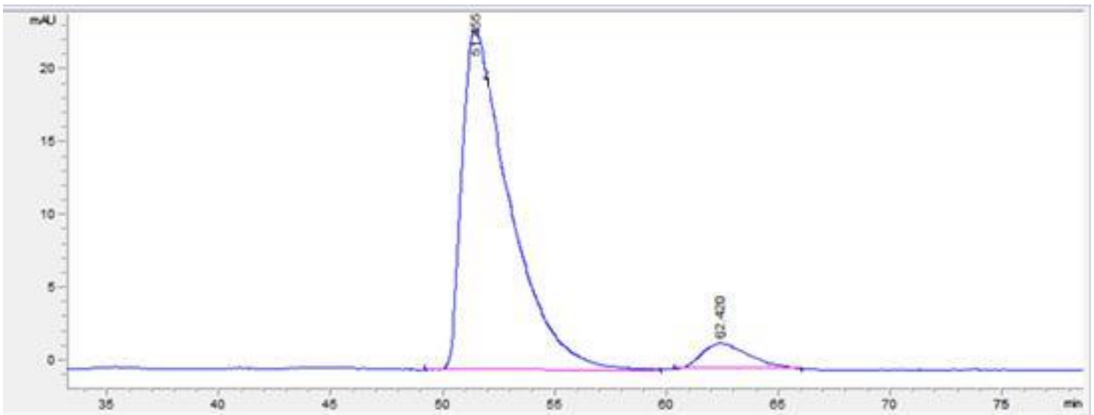

	Time/min	Area	Height	Area%
1	51.455	3514.2	23.4	93.100
2	62.42	260.5	1.8	6.900

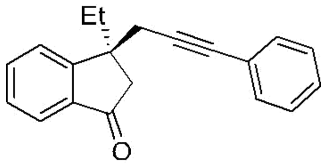

(*S*)-3-ethyl-3-(3-phenylprop-2-yn-1-yl)-2,3-dihydro-1*H*-inden-1-one (2r)

The mobile phase for flash chromatography: hexane/ethyl acetate = 15:1. Yellow oil. (53.2 mg, 97%). $[\alpha]^{25}_{D}$ = -11.7 (c = 0.68, $CHCl_3$). ^{1}H NMR (400 MHz, $CDCl_3$): δ 7.77 (d, *J* = 7.6 Hz, 1H), 7.65 (t, *J* = 7.6 Hz, 1H), 7.60 (d, *J* = 7.6 Hz, 1H), 7.42 (t, *J* = 7.6 Hz, 1H), 7.28-7.22 (m, 5H), 2.84 (d, *J* = 16.8 Hz, 1H), 2.80 (d, *J* = 16.8 Hz, 1H), 2.79 (d, *J* = 18.8 Hz, 1H), 2.68 (d, *J* = 18.8 Hz, 1H), 2.03 (dt, *J* = 14.4, 7.2 Hz, 1H), 1.93 (dt, *J* = 14.4, 7.2 Hz, 1H), 0.81 (t, *J* = 7.2 Hz, 3H). ^{13}C NMR (100 MHz, $CDCl_3$): δ 205.3, 159.6, 137.3, 134.9, 131.6, 128.3, 128.1, 128.0, 124.3, 123.4, 86.6, 83.1, 47.4, 46.1, 32.1, 32.0, 9.2. IR: υ 1715, 1602, 1490, 1461, 1442, 1404, 1285, 1238, 1089, 1070, 756, 691 cm^{-1}. HRMS (ESI-TOF) m/z: $[M + Na]^+$ Calcd for $C_{20}H_{18}NaO$, 297.1250; found 297.1259. Enantiomeric excess was determined by HPLC with a Chiralpak OD column (hexane: 2-propanol = 95:5, 1.0 mL/min, 254 nm, 94:6 *er*); major enantiomer t_r =7.9 min, minor enantiomer t_r =8.7 min.

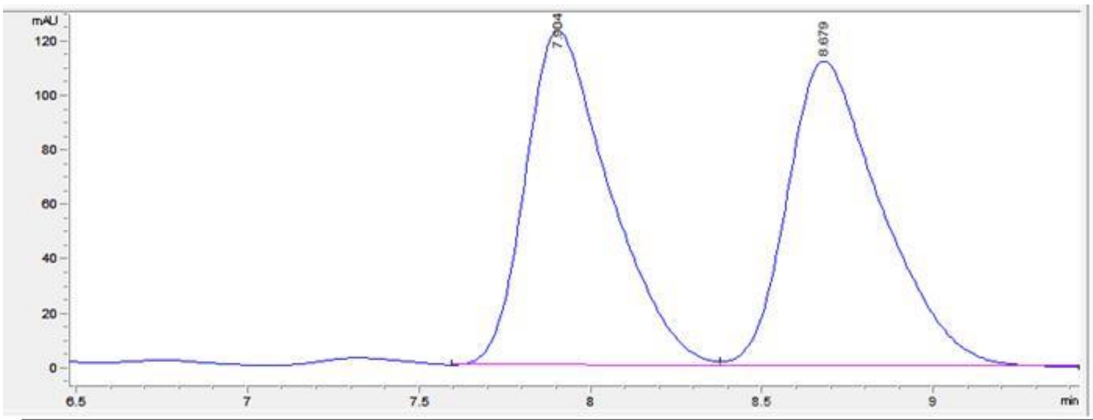

	Time/min	Area	Height	Area%
1	7.904	2105.2	122.8	49.599
2	8.679	2139.2	111.9	50.401

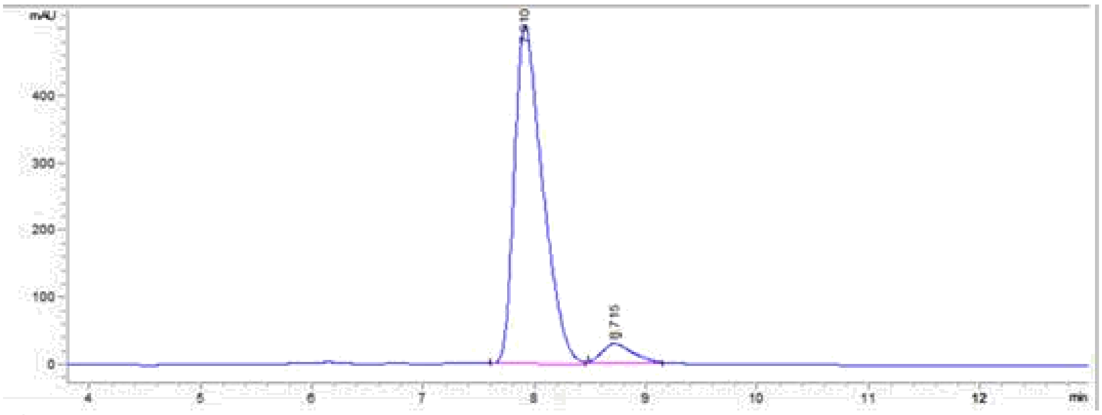

	Time/min	Area	Height	Area%
1	7.91	8926.9	504.7	94.220
2	8.715	547.6	28.9	5.780

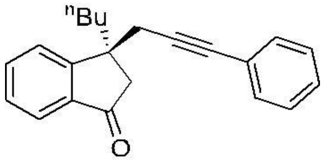

(*S*)-3-butyl-3-(3-phenylprop-2-yn-1-yl)-2,3-dihydro-1*H*-inden-1-one (2s)

The mobile phase for flash chromatography: hexane/ethyl acetate = 15:1. Yellow oil. (61.0 mg, 95%). $[\alpha]^{25}_{D}$ = -1.4 (c = 1.0, $CHCl_3$). ^1H NMR (400 MHz, $CDCl_3$): δ 7.66 (d, *J* = 7.6 Hz, 1H), 7.55 (t, *J* = 7.6 Hz, 1H), 7.51 (d, *J* = 7.6 Hz, 1H), 7.32 (t, *J* = 7.6 Hz, 1H), 7.18-7.12 (m, 5H), 2.74 (d, *J* = 17.2 Hz, 1H), 2.70 (d, *J* = 18.8 Hz, 1H), 2.69 (d, *J* = 17.2 Hz, 1H), 2.60 (d, *J* = 18.8 Hz, 1H), 1.95-1.85 (m, 1H), 1.80-1.71 (m, 1H), 1.26-1.14 (m, 3H), 0.93-0.84 (m, 1H), 0.77 (t, *J* = 7.2 Hz, 3H); ^{13}C NMR (100 MHz, $CDCl_3$): δ 205.3, 160.0, 137.1, 134.9, 131.6, 128.3, 128.1, 128.0, 124.3, 123.42, 123.38, 86.6, 83.1, 48.0, 45.7, 39.3, 32.3, 27.1, 23.2, 14.0. IR: υ 1711, 1609, 1511, 1462, 1385, 1286, 1248, 1178, 1096, 1033, 803, 758 cm^{-1}. HRMS (ESI-TOF) m/z: $[M + Na]^+$ Calcd for $C_{22}H_{22}NaO$, 325.1563; found 325.1572. Enantiomeric excess was determined by HPLC with a Chiralpak OD column (hexane: 2-propanol = 95:5, 1.0 mL/min, 254 nm, 92:8 *er*); major enantiomer t_r =6.6 min, minor enantiomer t_r =7.8 min.

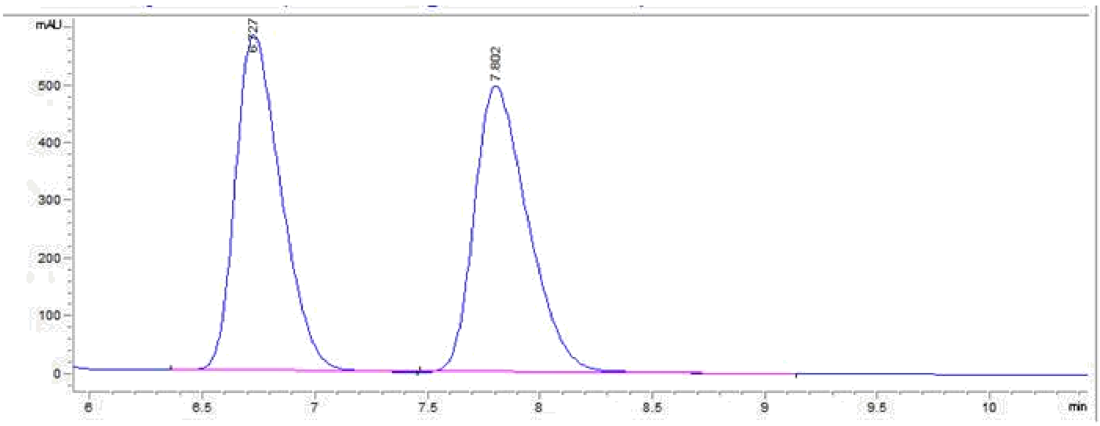

	Time/min	Area	Height	Area%
1	6.727	8225.5	582.4	49.946
2	7.802	8243.2	496.5	50.054

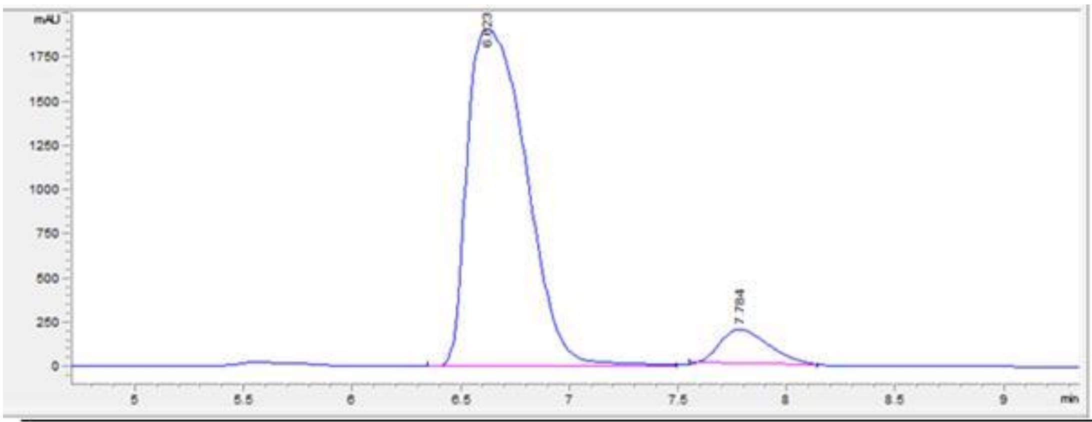

	Time/min	Area	Height	Area%
1	6.623	35988.5	1908.2	92.198
2	7.784	3045.4	195.4	7.802

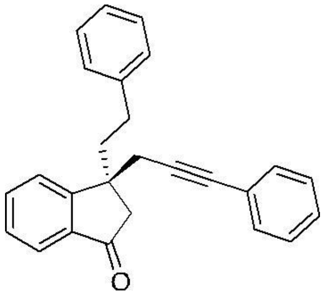

(*S*)-3-phenethyl-3-(3-phenylprop-2-yn-1-yl)-2,3-dihydro-1*H*-inden-1-one (2t)

The mobile phase for flash chromatography: hexane/ethyl acetate = 15:1. Yellow oil. (62.1 mg, 94%). $[\alpha]^{25}_{D}$ = +13.2 (c = 0.63, $CHCl_3$). ^1H NMR (400 MHz, $CDCl_3$): δ 7.73

(d, J = 7.6 Hz, 1H), 7.62-7.57 (m, 2H), 7.42-7.36 (m, 1H), 7.24-7.17 (m, 7H), 7.11 (t, J = 7.2 Hz, 1H), 7.06 (d, J = 7.2 Hz, 2H), 2.85-2.77 (m, 3H), 2.73 (d, J = 18.8 Hz, 1H), 2.59-2.50 (m, 1H), 2.30 (d, J = 12.4 Hz, 1H), 2.25 (d, J = 12.4 Hz, 1H), 2.18-2.05 (m, 1H). ^{13}C NMR (100 MHz, $CDCl_3$): δ 204.9, 159.5, 141.6, 137.1, 135.1, 131.6, 128.6, 128.36, 128.32, 128.1, 126.2, 124.3, 123.6, 123.3, 86.3, 83.3, 47.9, 45.7, 41.5, 32.4, 31.5. IR: υ 1713, 1601, 1490, 1462, 1285, 1245, 1069, 756, 692 cm^{-1}. HRMS (ESI-TOF) m/z: $[M + Na]^+$ Calcd for $C_{26}H_{22}NaO$, 373.1563; found 373.1573. Enantiomeric excess was determined by HPLC with a Chiralpak OD column (hexane: 2-propanol = 95:5, 1.0 mL/min, 254 nm, 93:7 *er*); major enantiomer t_r =26.2 min, minor enantiomer t_r =24.7 min.

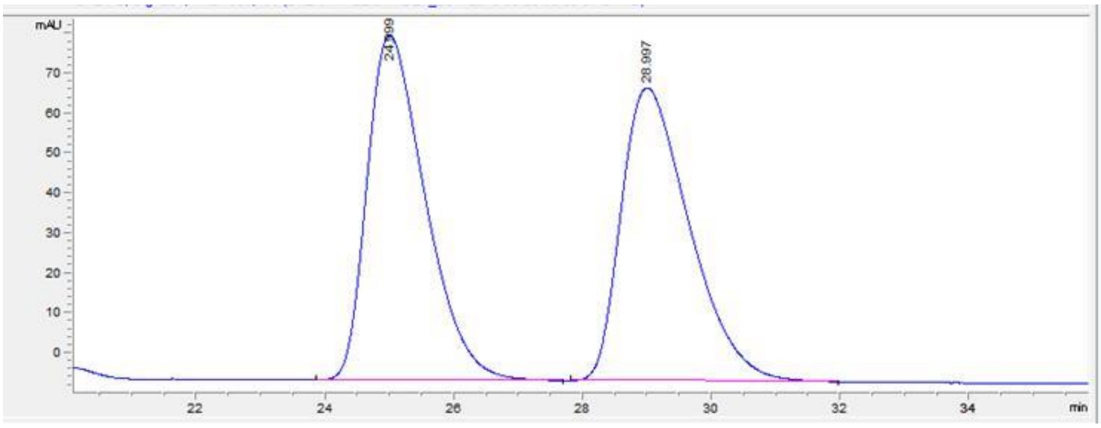

	Time/min	Area	Height	Area%
1	24.999	5433.2	86.4	50.075
2	28.997	5416.9	73.5	49.925

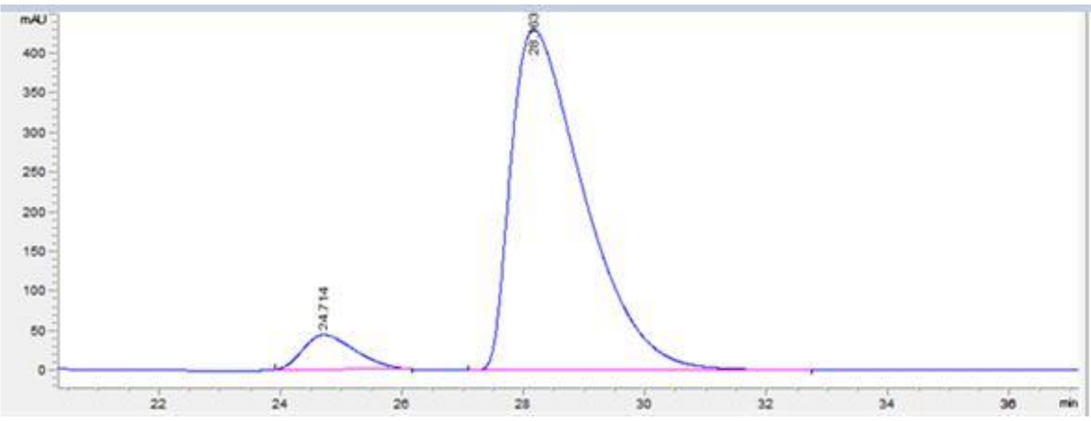

	Time/min	Area	Height	Area%
1	24.714	2782.5	45.1	7.214
2	26.163	35788.4	429.7	92.786

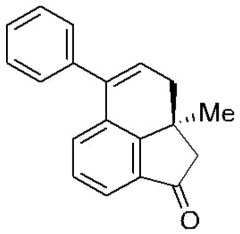

(*S*)-2a-methyl-5-phenyl-2a,3-dihydroacenaphthylen-1(2*H*)-one (3)

A vial was charged with **2b** (52 mg, 0.2 mmol), $Pd(OAc)_2$ (0.5 mg, 1.0 mol%), evacuated under high vacuum and backfilled with N_2. TFA and 1,4-dioxane (2:1, 1.5 mL) was next added. The mixture was stirred at 30 ℃. Upon reaction completion (12 h, TLC, eluent: hexane-EtOAc, 20:1), EtOAc (50 mL) and water (50 mL) were added. The phases were separated and the aqueous layer was extracted with EtOAc (50 mL). The combined organic extracts were washed with aqueous saturated $NaHCO_3$ (50 mL) and brine (50 mL), dried over anhydrous Na_2SO_4, filtered, and concentrated in vacuum. The mobile phase for flash chromatography: hexane/ethyl acetate = 50:1. White solid. (43.2 mg, 83%). mp 103-105 ^{o}C. $[\alpha]^{25}_{D}$ = -252.6 (c = 0.94, $CHCl_3$). ^{1}H NMR (400 MHz, $CDCl_3$) δ 7.52 (d, *J* = 6.4 Hz, 1H), 7.41-7.30 (m, 5H), 7.25-7.19 (m, 2H), 6.06 (dd, *J* = 6.4, 2.4 Hz, 1H), 2.71 (d, *J* = 17.2 Hz, 1H), 2.65 (dd, *J* = 16.8, 6.4 Hz, 1H), 2.59 (d, *J* = 17.2 Hz, 1H), 2.52 (dd, *J* = 16.8, 2.4 Hz, 1H), 1.32 (s, 3H). ^{13}C NMR (100 MHz, $CDCl_3$) δ 205.6, 159.4, 138.9, 137.9, 133.9, 132.4, 129.6, 128.7, 128.5, 128.3, 127.9, 127.6, 122.4, 54.4, 36.95, 36.88, 26.7. IR: υ 1715, 1706, 1571, 1477, 1448, 1369, 1260, 1241, 1096, 1030, 1017, 800, 754, 701 cm^{-1}. HRMS (ESI-TOF) m/z: $[M + H]^{+}$ Calcd for $C_{19}H_{17}O$, 261.1274; found 261.1265. Enantiomeric excess was determined by HPLC with a Chiralpak OD column (hexanes: 2-propanol = 99:1, 1.0 mL/min, 254 nm, 94.5:5.5 *er*); major enantiomer t_r = 8.4 min, minor enantiomer t_r = 9.8 min.

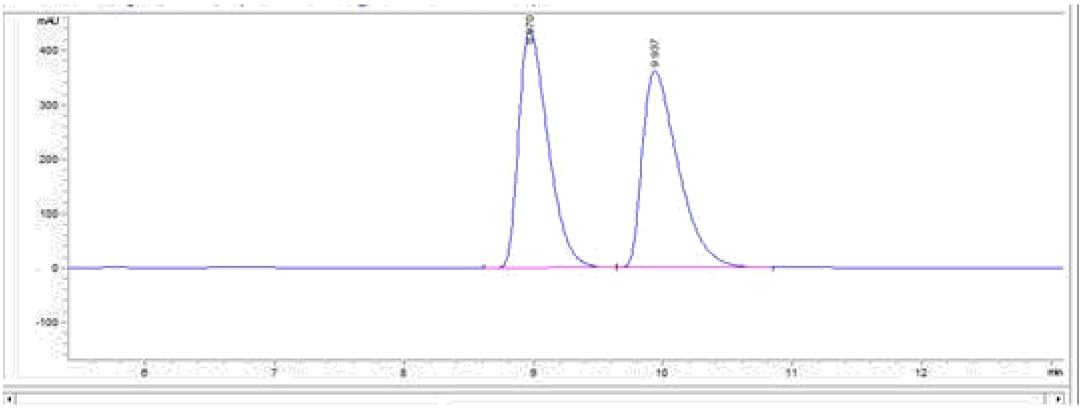

	Time/min	Area	Height	Area%
1	8.97	6917.6	435.3	49.694
2	9.937	7002.9	361.6	50.306

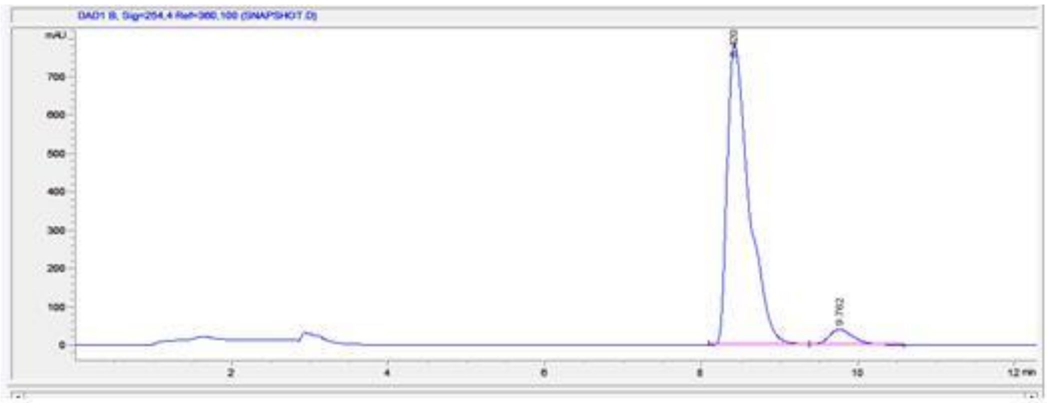

	Time/min	Area	Height	Area%
1	8.42	15246.8	783.9	94.451
2	9.762	895.7	40.9	5.549

References

(1) C. He, M. Hou, Z. Zhu and Z. Gu, *ACS Catal.* 2017, **7**, 5316.

(2) J. Cao, L. Chen, F.-N. Sun, Y.-L. Sun, K.-Z. Jiang, K.-F. Yang, Z. Xu and L.-W. Xu, *Angew. Chem. Int. Ed.* 2019, **58**, 897.

(3) M. R. Albicker and N. Cramer, *Angew. Chem. Int. Ed.* 2009, **48**, 9139.

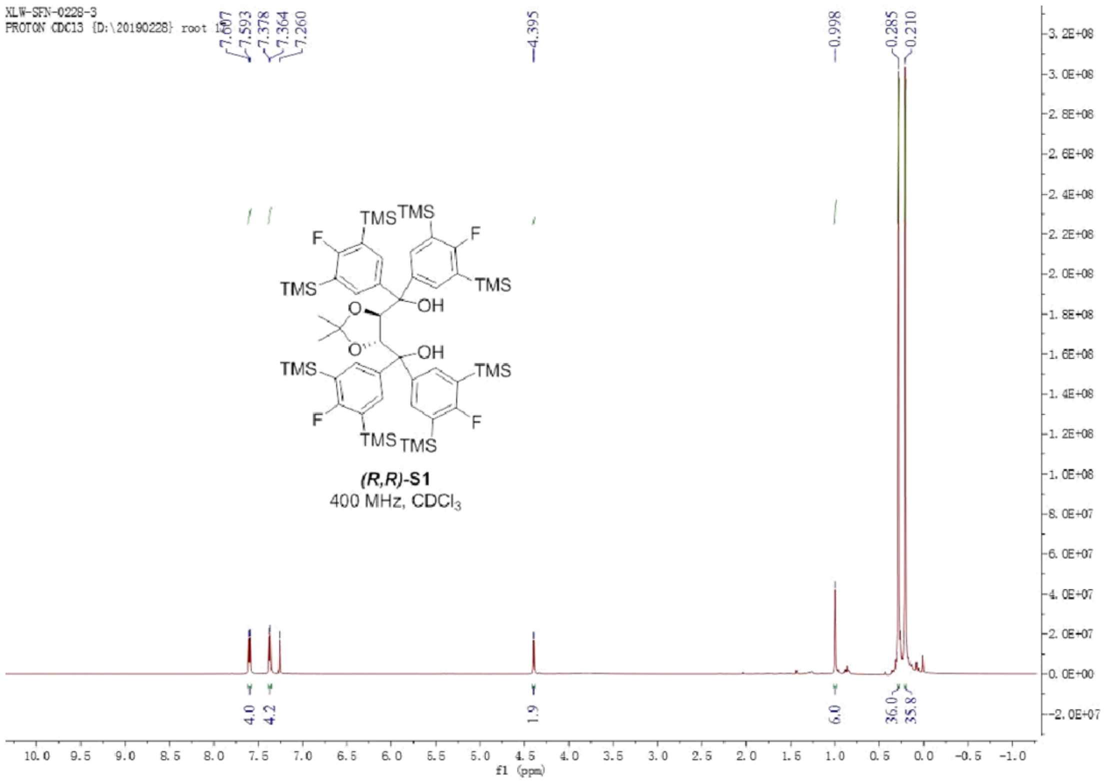
XLW-SFN-0228-3
PROTON CDCl3 {D:\20190228} root
7.607
7.593
7.378
7.364
7.260
4.395
0.998
0.285
0.210
TMS
F
OH
(R,R)-S1
400 MHz, CDCl3
4.0
4.2
1.9
6.0
36.0
35.8
f1 (ppm)

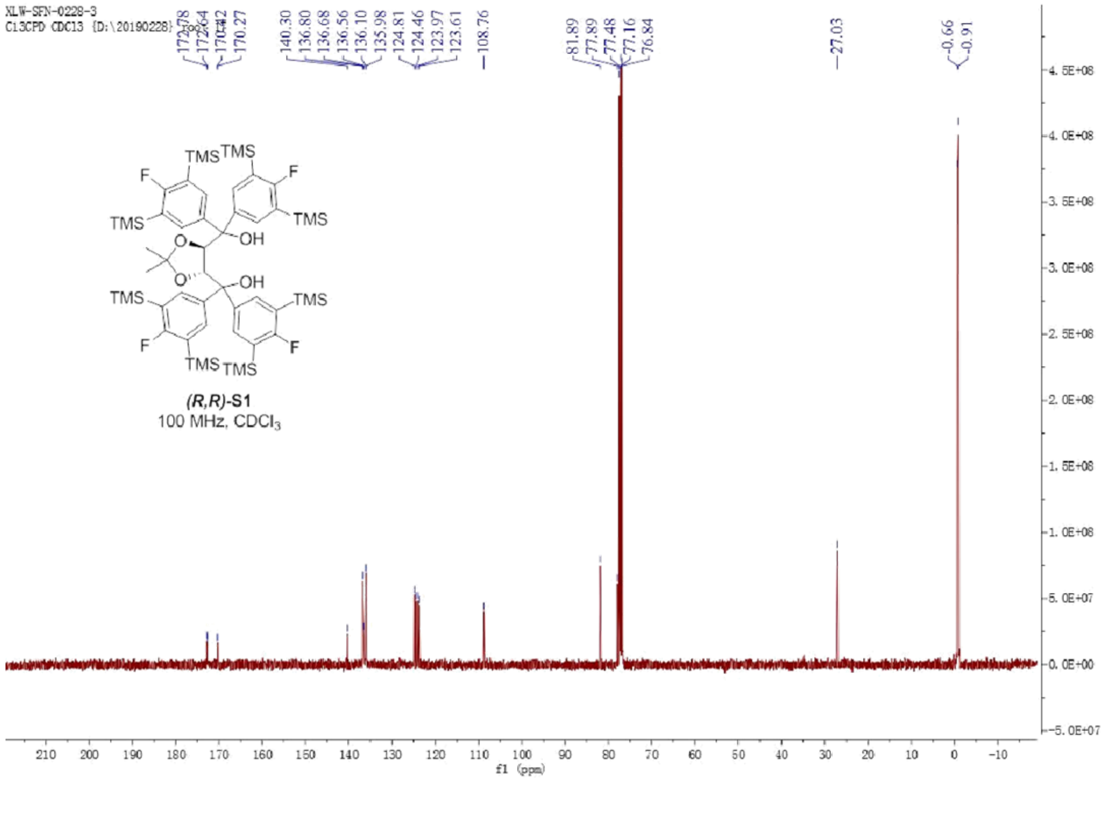
XLW-SFN-0228-3
C13CPD CDCl3 {D:\20190228} root
170.27
140.30
136.80
136.68
136.56
136.10
135.98
124.81
124.46
123.97
123.61
108.76
81.89
77.89
77.48
77.16
76.84
27.03
-0.66
-0.91
TMS
F
OH
(R,R)-S1
100 MHz, CDCl3
f1 (ppm)

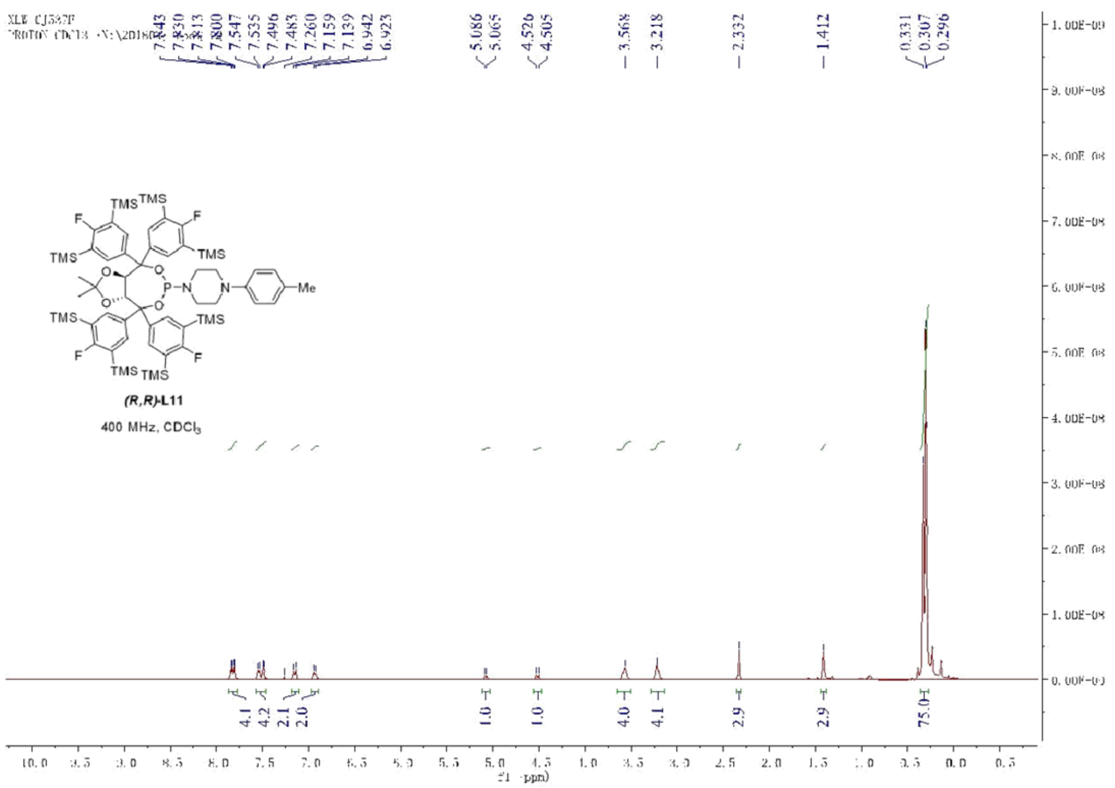
(R,R)-L11
400 MHz, CDCl3

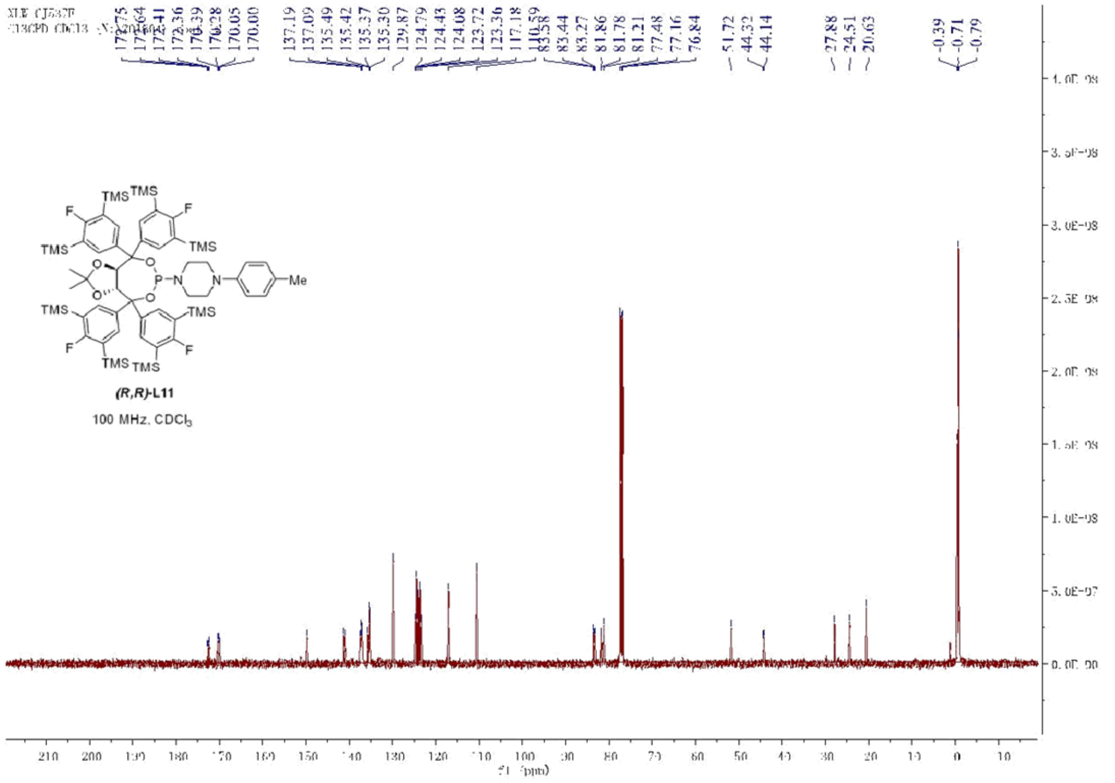
(R,R)-L11
100 MHz, CDCl3

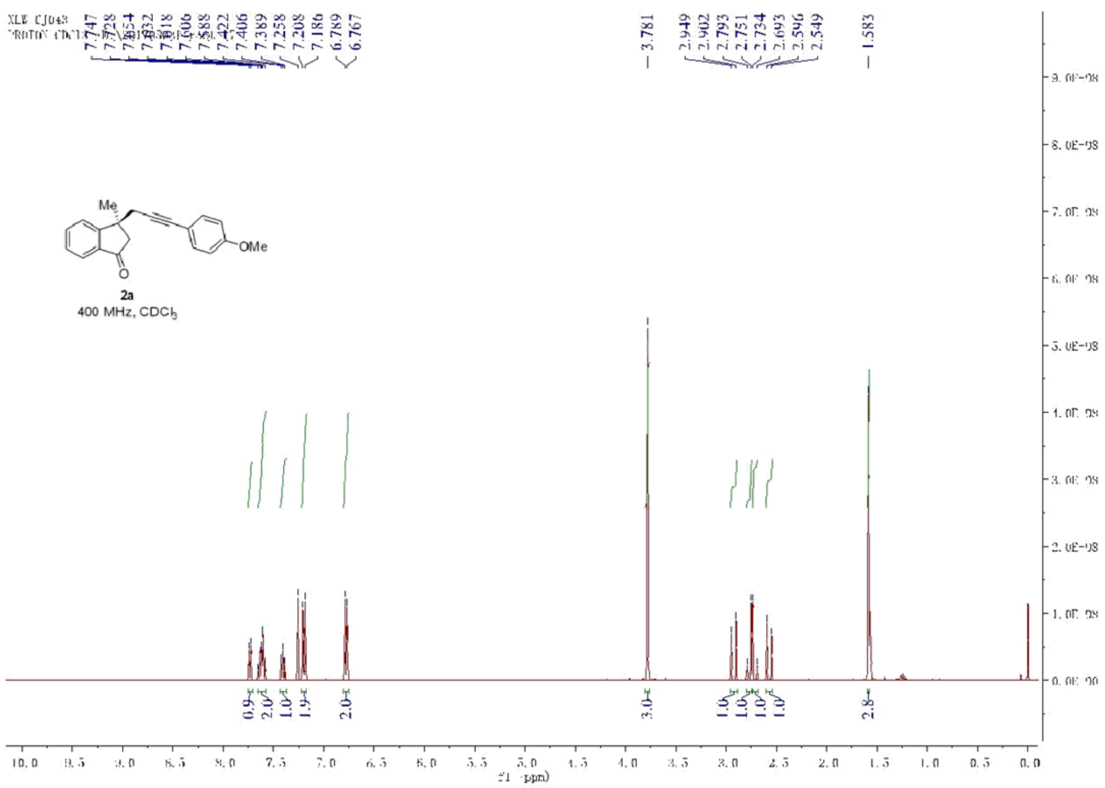
7.747
7.728
7.654
7.632
7.618
7.606
7.588
7.422
7.406
7.389
7.258
7.208
7.186
6.789
6.767
3.781
2.949
2.902
2.793
2.751
2.734
2.693
2.596
2.549
1.583
Me
OMe
O
2a
400 MHz, CDCl3
f1 (ppm)

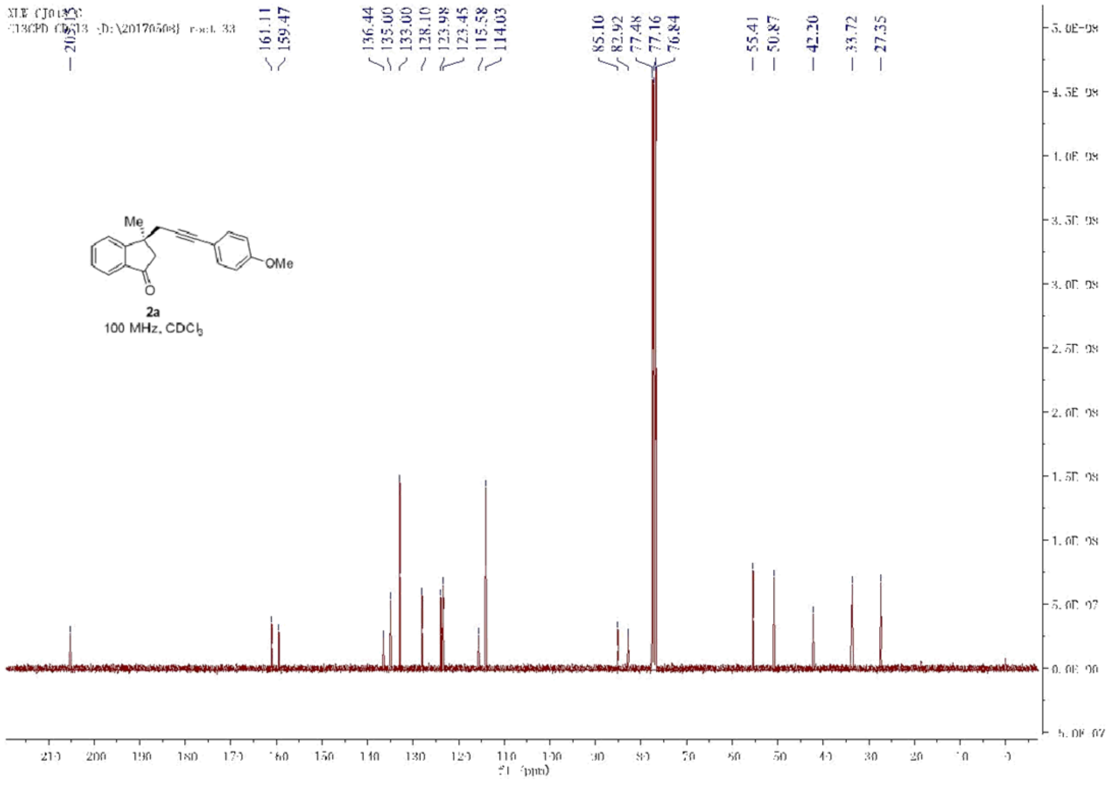
161.11
159.47
136.44
135.00
133.00
128.10
123.98
123.45
115.58
114.03
85.10
82.92
77.48
77.16
76.84
55.41
50.87
42.20
33.72
27.35
Me
OMe
O
2a
100 MHz, CDCl3
f1 (ppm)

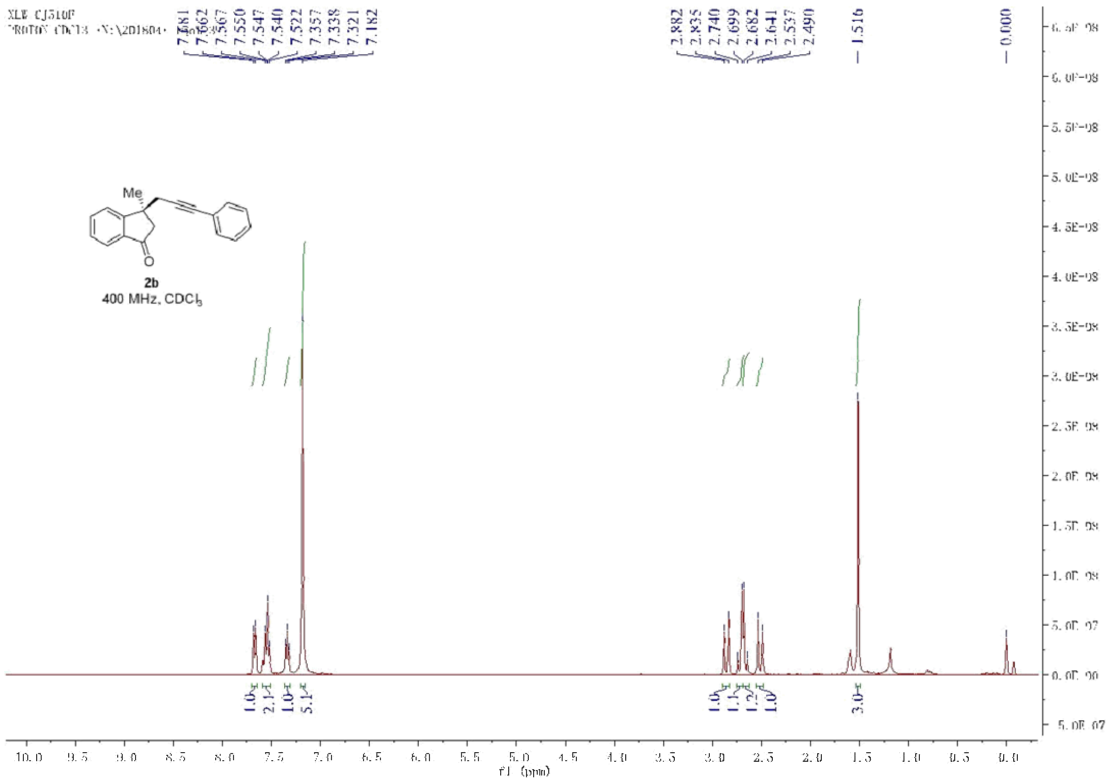
7.681
7.662
7.567
7.550
7.547
7.540
7.522
7.357
7.338
7.321
7.182
2.882
2.835
2.740
2.699
2.682
2.641
2.537
2.490
1.516
0.000
Me
O
2b
400 MHz, CDCl3
1.0
2.1
1.0
5.1
1.0
1.1
1.2
1.0
3.0
f1 (ppm)

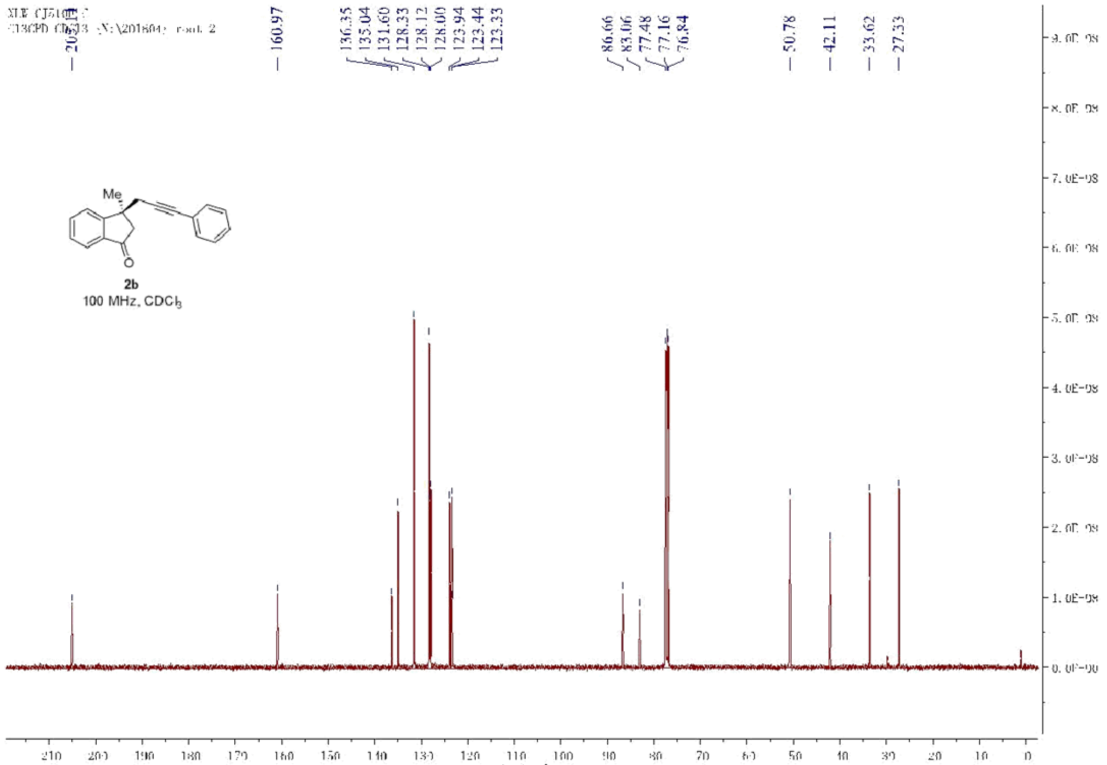
20[illegible]
160.97
136.35
135.04
131.60
128.33
128.12
128.00
123.94
123.44
123.33
86.66
83.06
77.48
77.16
76.84
50.78
42.11
33.62
27.33
Me
O
2b
100 MHz, CDCl3
f1 (ppm)

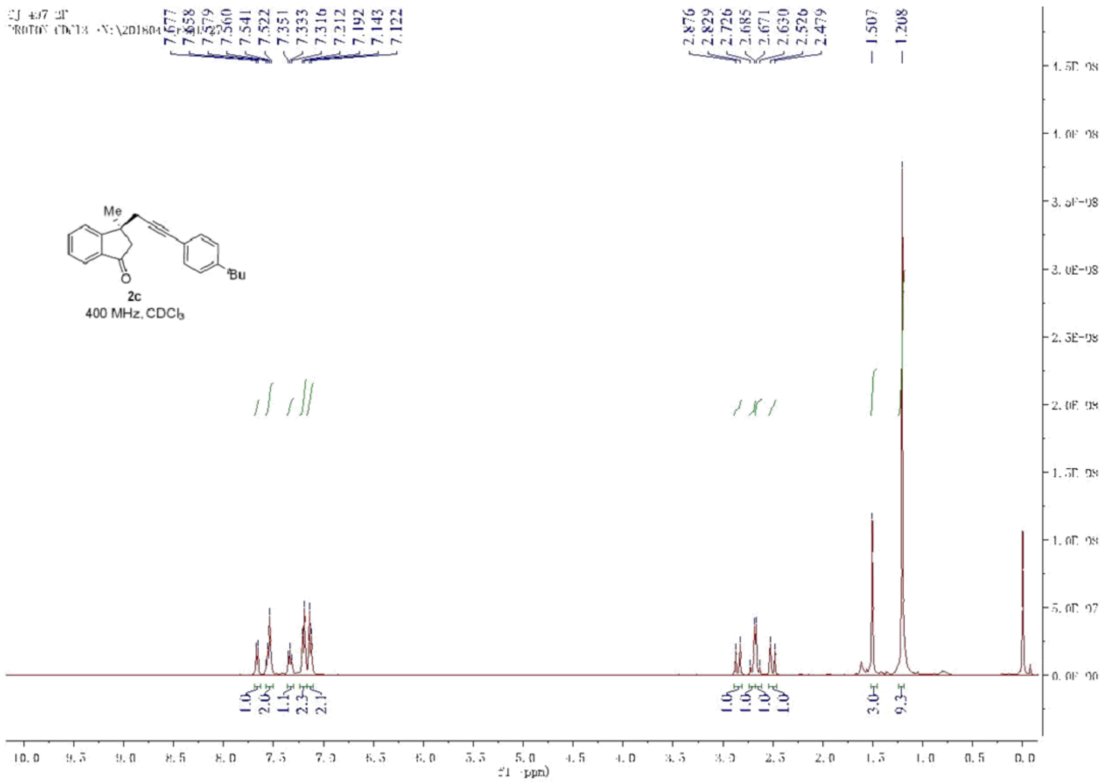

7.677 7.658 7.579 7.560 7.541 7.522 7.351 7.333 7.316 7.212 7.192 7.143 7.122
2.876 2.829 2.726 2.685 2.671 2.630 2.526 2.479
1.507
1.208
Me
tBu
2c
400 MHz, CDCl3
1.0 2.0 1.1 2.3 2.1
1.0 1.0 1.0 1.0
3.0
9.3
f1 (ppm)

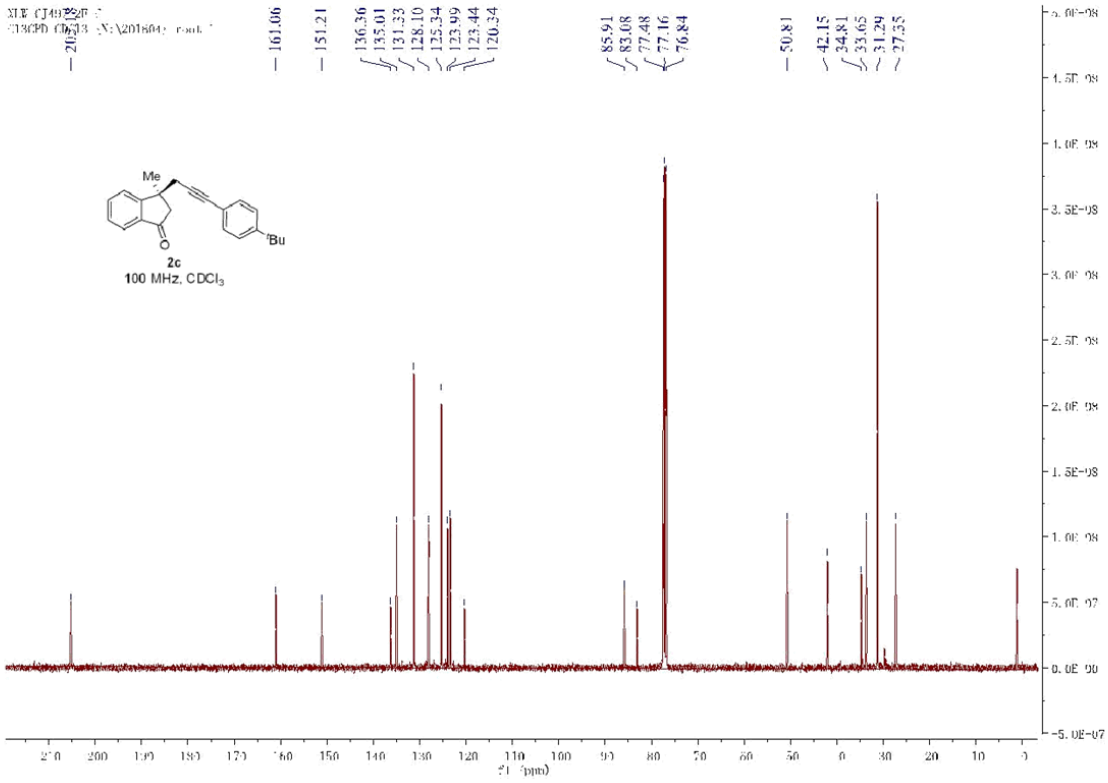

161.06
151.21
136.36 135.01 131.33 128.10 125.34 123.99 123.44 120.34
85.91 83.08 77.48 77.16 76.84
50.81
42.15 34.81 33.65 31.29 27.55
Me
tBu
2c
100 MHz, CDCl3
f1 (ppm)

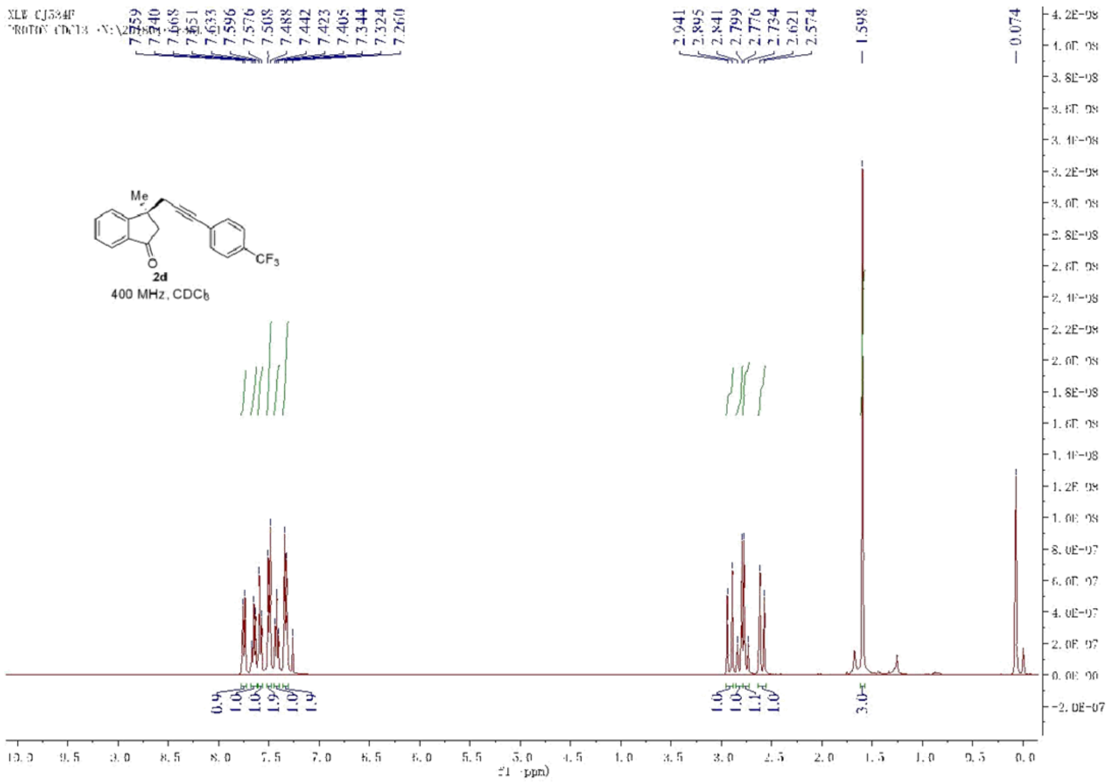
Me
CF3
2d
400 MHz, CDCl3
f1 (ppm)

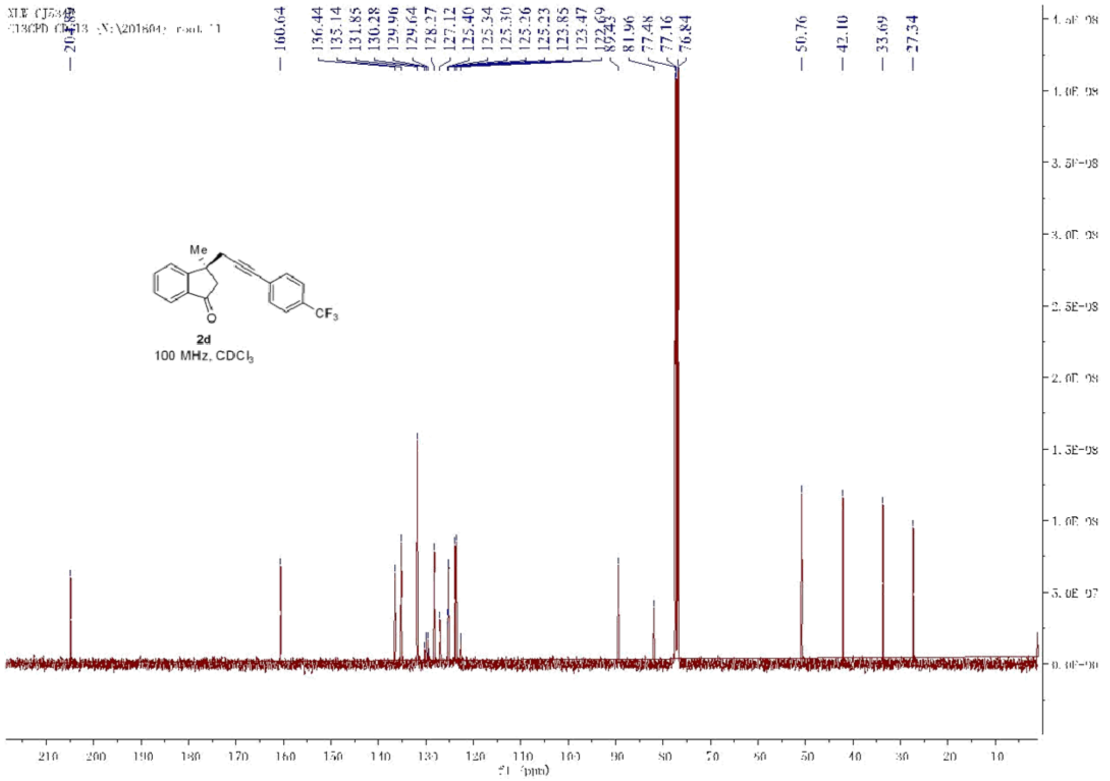
160.64
81.96
77.48
77.16
76.84
50.76
42.10
33.69
27.34
Me
CF3
2d
100 MHz, CDCl3
f1 (ppm)

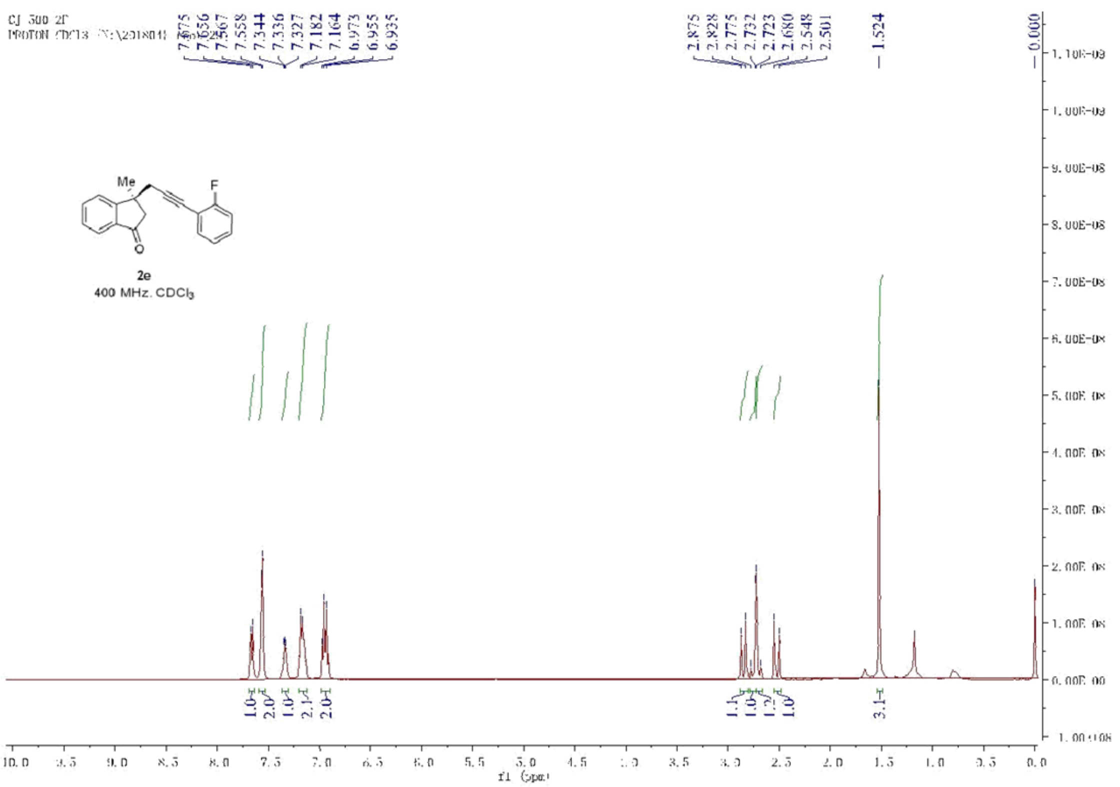
7.675
7.656
7.567
7.558
7.344
7.336
7.327
7.182
7.164
6.973
6.955
6.935
2.875
2.828
2.775
2.732
2.723
2.680
2.548
2.501
1.524
0.000
Me
F
O
2e
400 MHz, CDCl3
1.0
2.0
1.0
2.1
2.0
1.1
1.0
1.2
1.0
3.1
f1 (ppm)

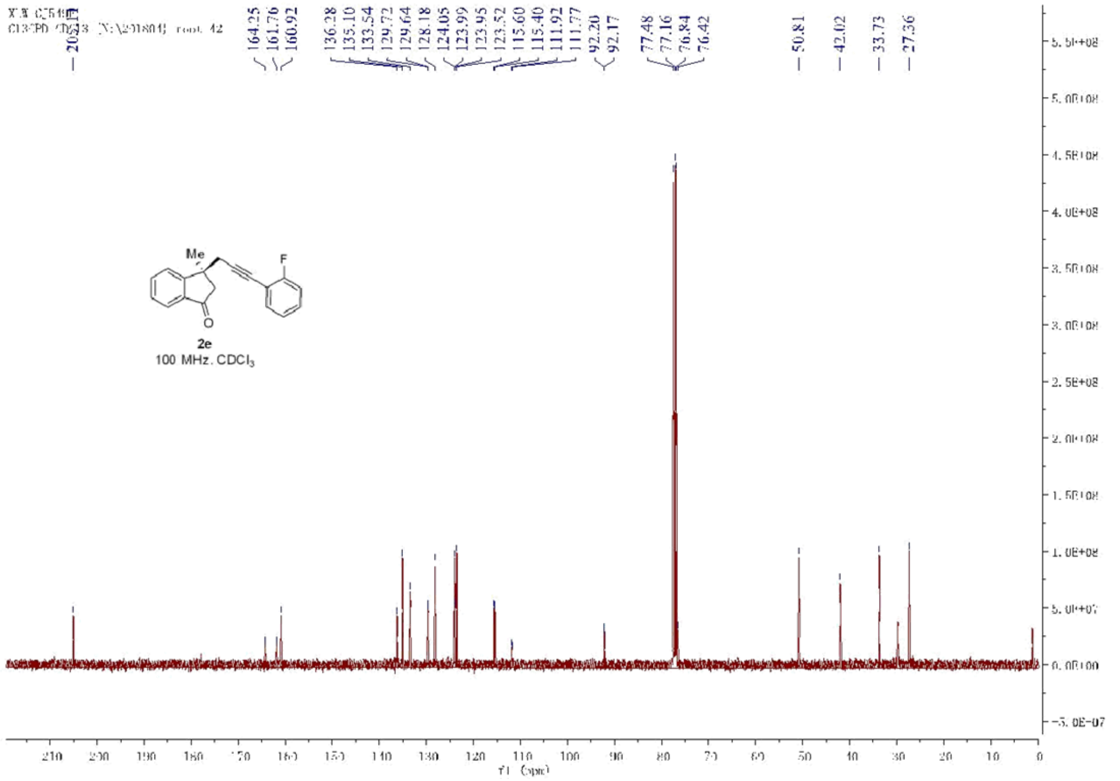
164.25
161.76
160.92
136.28
135.10
133.54
129.72
129.64
128.18
124.05
123.99
123.95
123.52
115.60
115.40
111.92
111.77
92.20
92.17
77.48
77.16
76.84
76.42
50.81
42.02
33.73
27.36
Me
F
O
2e
100 MHz, CDCl3
f1 (ppm)

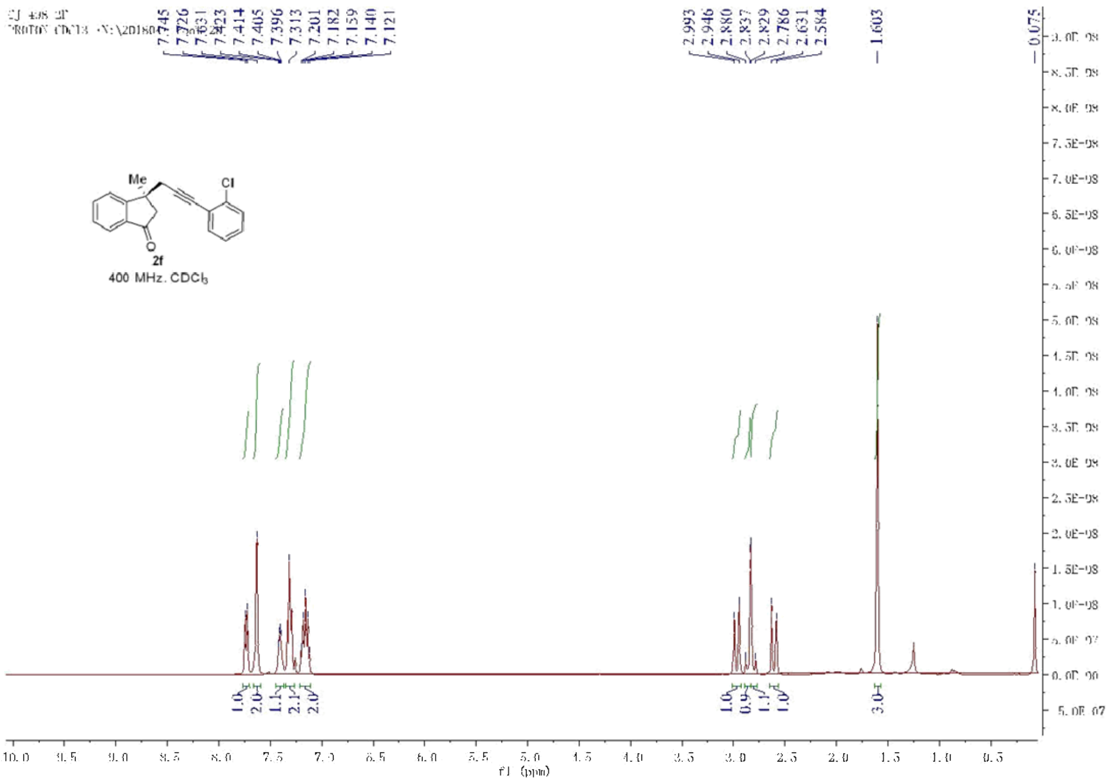
7.745 7.726 7.631 7.423 7.414 7.405 7.396 7.313 7.201 7.182 7.159 7.140 7.121
2.993 2.946 2.880 2.837 2.829 2.786 2.631 2.584
1.603
0.075
Me
Cl
O
2f
400 MHz, CDCl3
1.0 2.0 1.1 2.1 2.0
1.0 0.9 1.1 1.0
3.0
f1 (ppm)

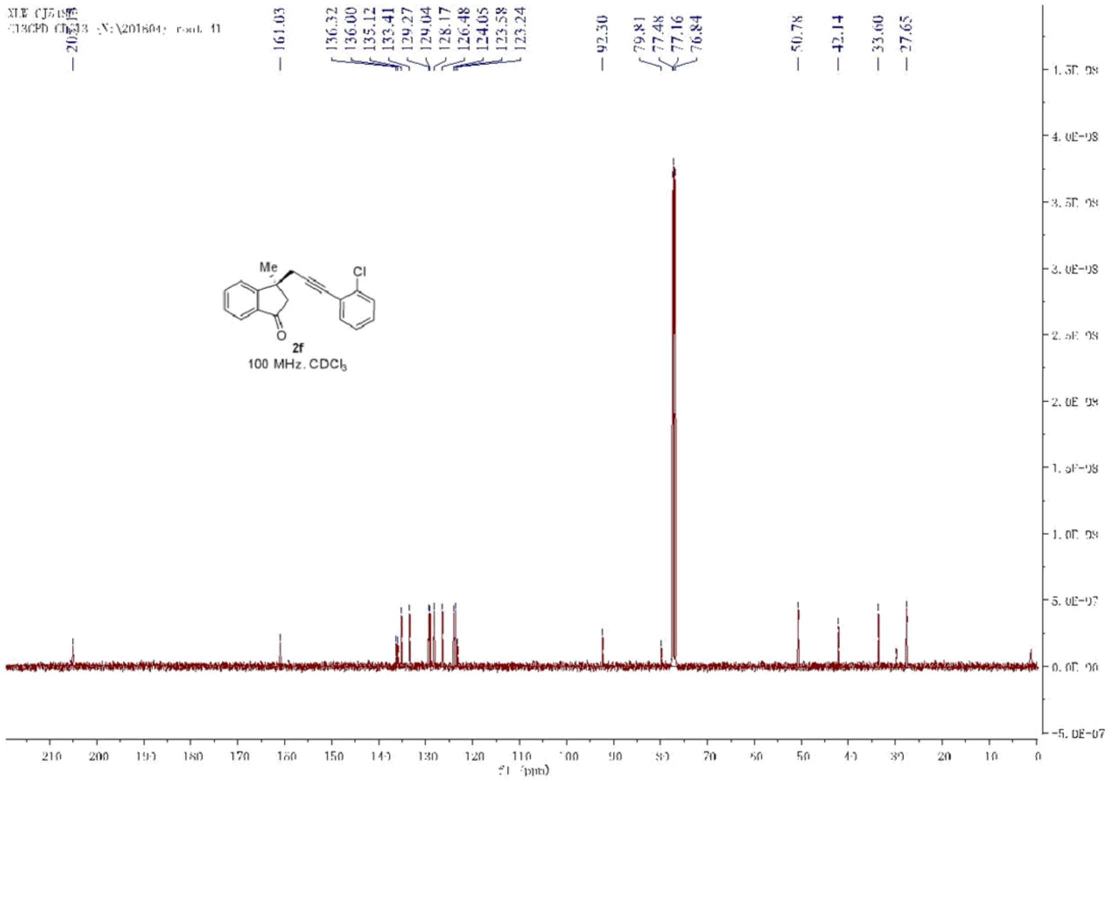
161.03
136.32 136.00 135.12 133.41 129.27 129.04 128.17 126.48 124.05 123.58 123.24
92.30
79.81 77.48 77.16 76.84
50.78
42.14
33.60
27.65
Me
Cl
O
2f
100 MHz, CDCl3
f1 (ppm)

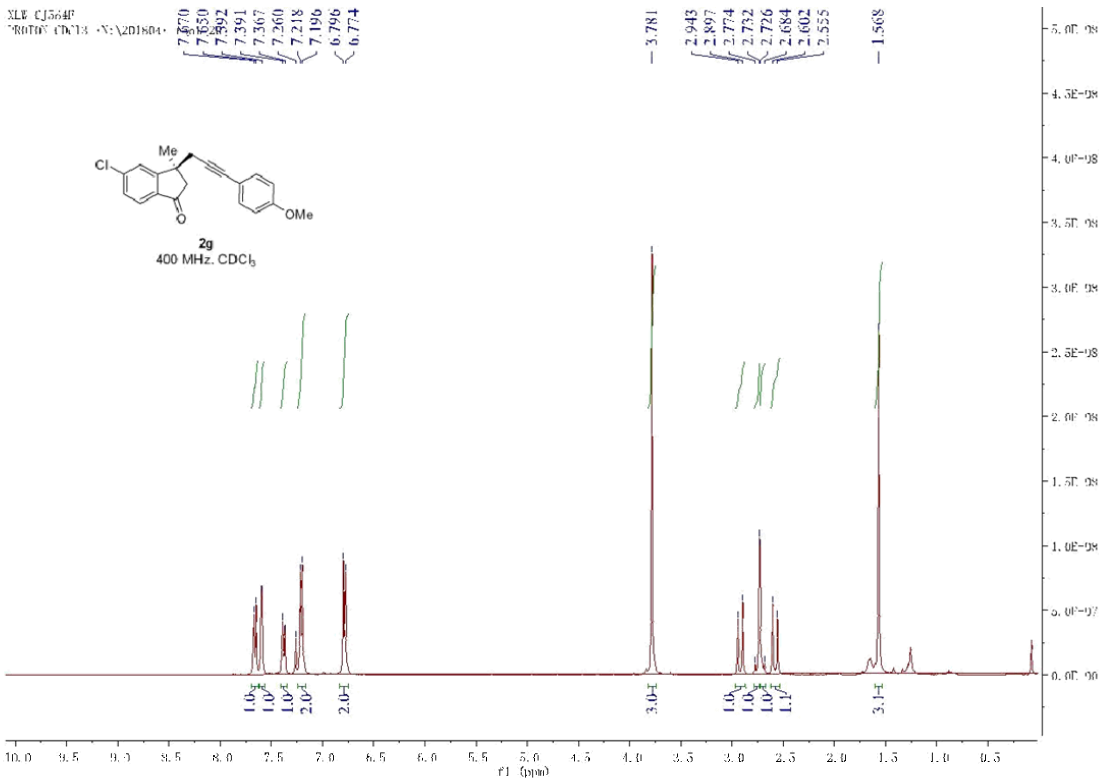
7.670
7.650
7.392
7.391
7.367
7.260
7.218
7.196
6.796
6.774
3.781
2.943
2.897
2.774
2.732
2.726
2.684
2.602
2.555
1.568
Cl
Me
O
OMe
2g
400 MHz. CDCl3
1.0
1.0
1.0
2.0
2.0
3.0
1.0
1.0
1.0
1.1
3.1
f1 (ppm)

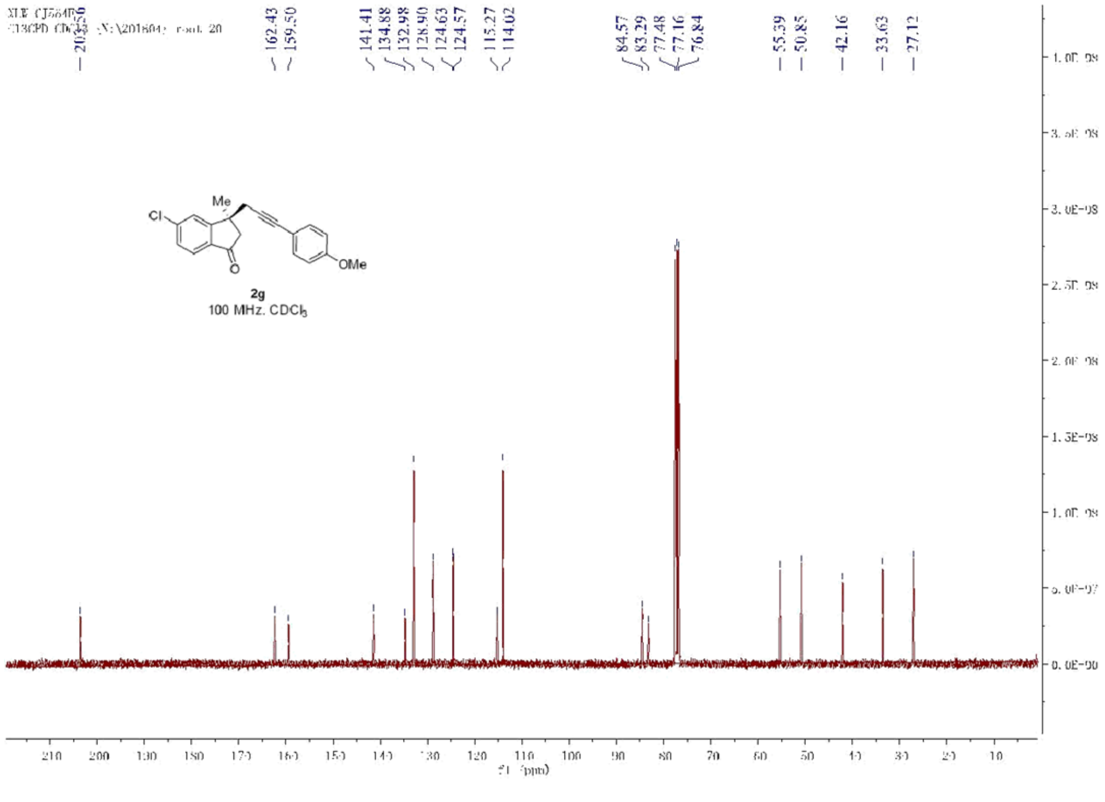
162.43
159.50
141.41
134.88
132.98
128.90
124.63
124.57
115.27
114.02
84.57
83.29
77.48
77.16
76.84
55.39
50.85
42.16
33.63
27.12
Cl
Me
O
OMe
2g
100 MHz. CDCl3
f1 (ppm)

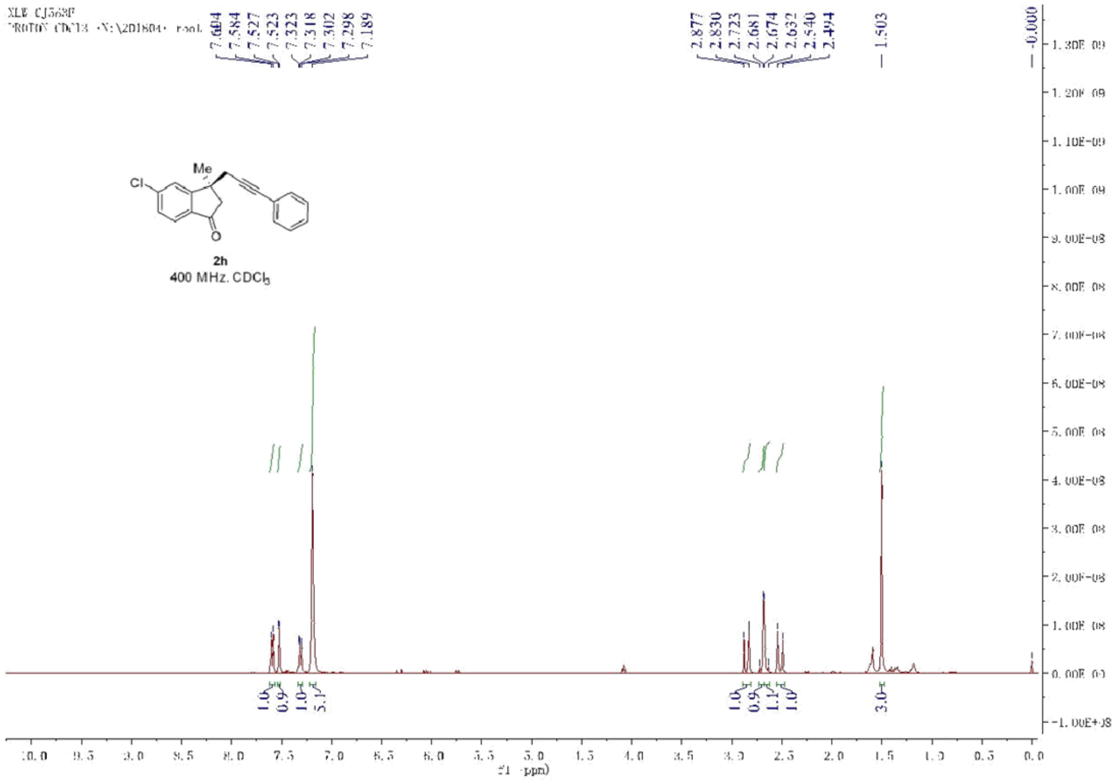
7.604
7.584
7.527
7.523
7.323
7.318
7.302
7.298
7.189
2.877
2.830
2.723
2.681
2.674
2.632
2.540
2.494
1.503
-0.000
Cl
Me
O
2h
400 MHz, $CDCl_3$
1.0
0.9
1.0
5.1
1.0
0.9
1.1
1.0
3.0
f1 (ppm)

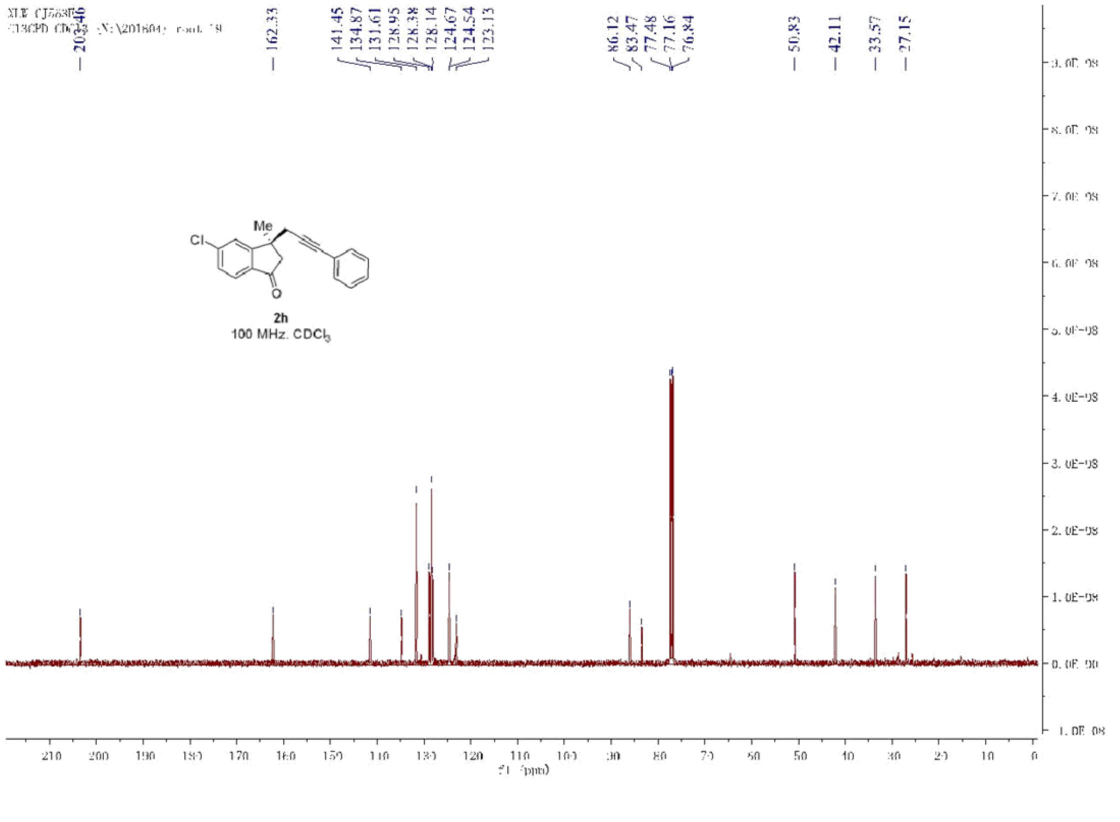
162.33
141.45
134.87
131.61
128.95
128.38
128.14
124.67
124.54
123.13
86.12
83.47
77.48
77.16
76.84
50.83
42.11
33.57
27.15
Cl
Me
O
2h
100 MHz, $CDCl_3$
f1 (ppm)

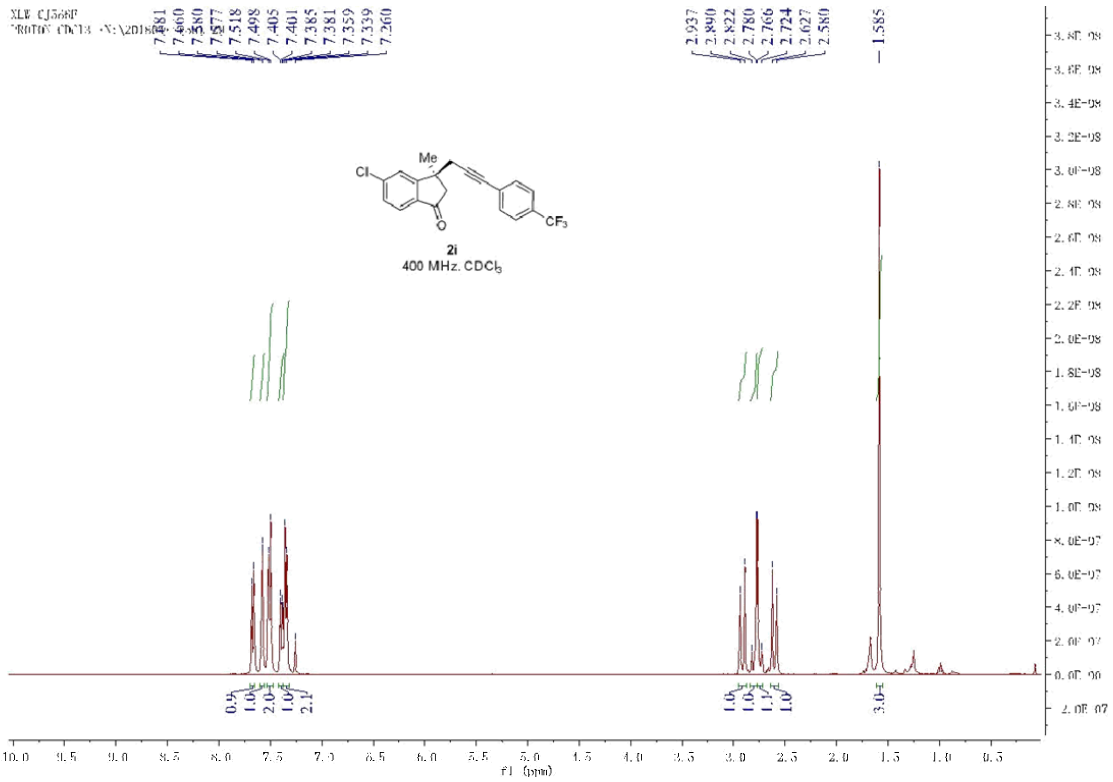
7.781
7.660
7.580
7.577
7.518
7.498
7.405
7.401
7.385
7.381
7.359
7.339
7.260
2.937
2.890
2.822
2.780
2.766
2.724
2.627
2.580
1.585
Me
Cl
O
CF3
2i
400 MHz, CDCl3
0.9
1.0
2.0
1.0
2.1
1.0
1.0
1.1
1.0
3.0
f1 (ppm)

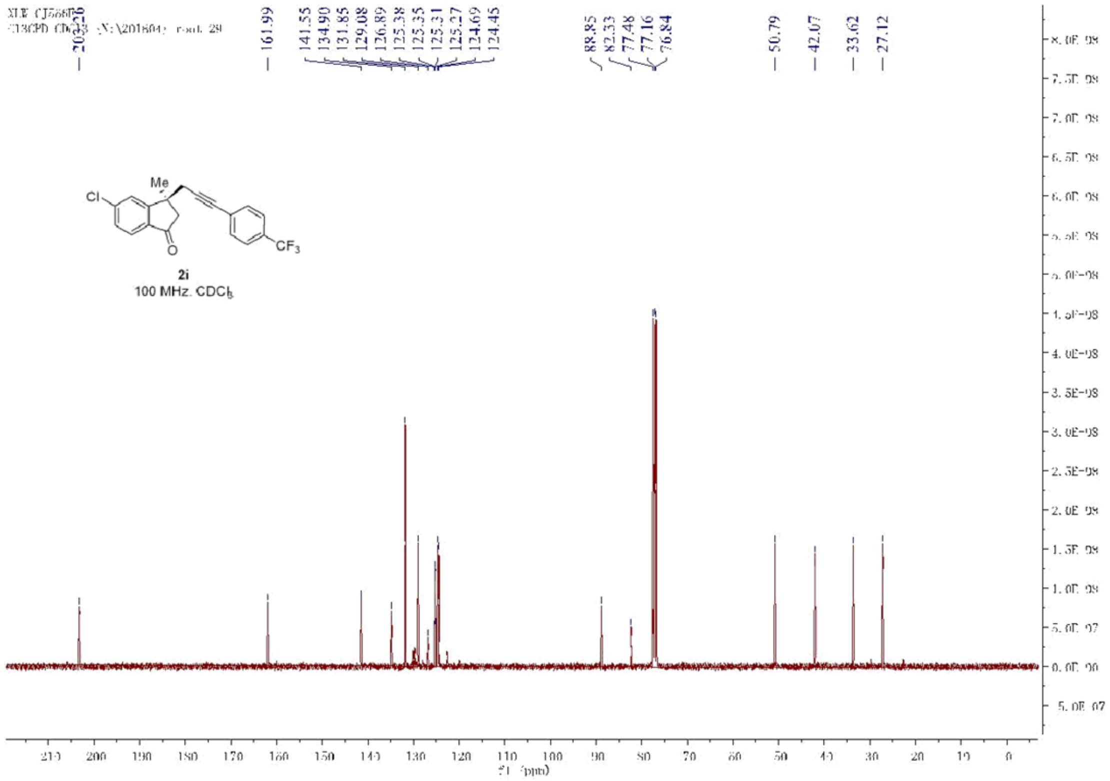
161.99
141.55
134.90
131.85
129.08
126.89
125.38
125.35
125.31
125.27
124.69
124.45
88.85
82.33
77.48
77.16
76.84
50.79
42.07
33.62
27.12
Me
Cl
O
CF3
2i
100 MHz, CDCl3
f1 (ppm)

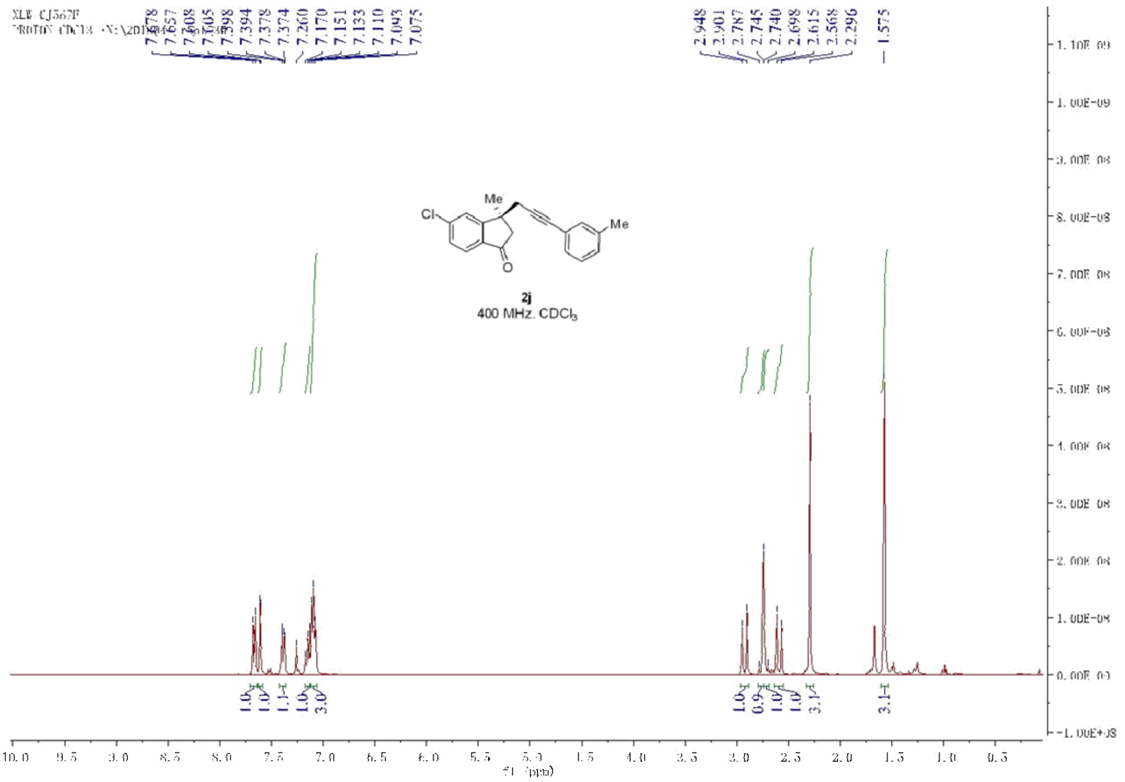
2j
400 MHz, CDCl3

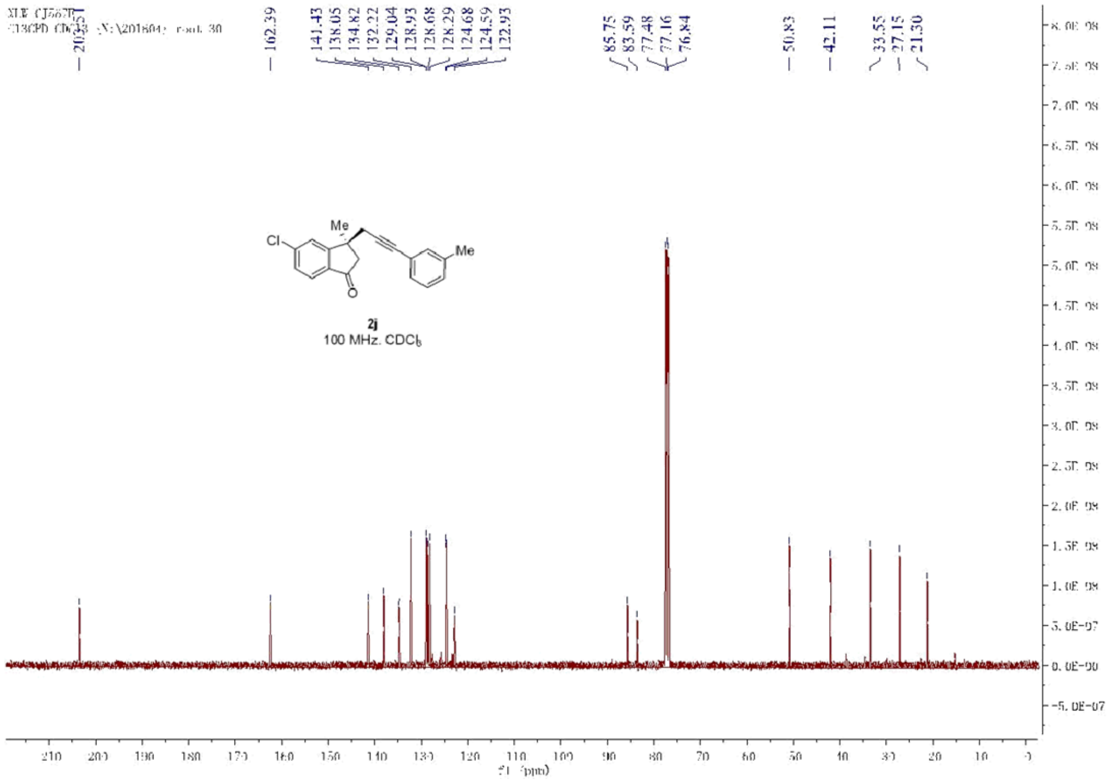
2j
100 MHz, CDCl3

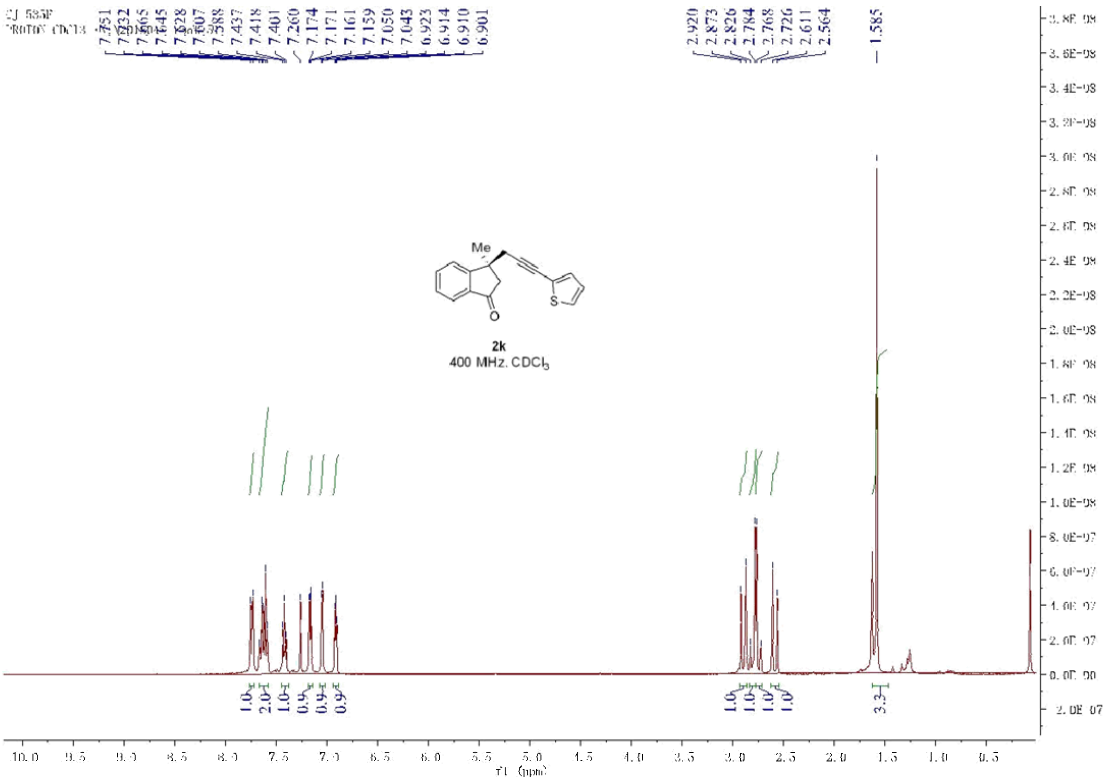

7.751
7.732
7.665
7.645
7.628
7.607
7.588
7.437
7.418
7.401
7.260
7.174
7.171
7.161
7.159
7.050
7.043
6.923
6.914
6.910
6.901
2.920
2.873
2.826
2.784
2.768
2.726
2.611
2.564
1.585
Me
S
O
2k
400 MHz, $CDCl_3$
f1 (ppm)

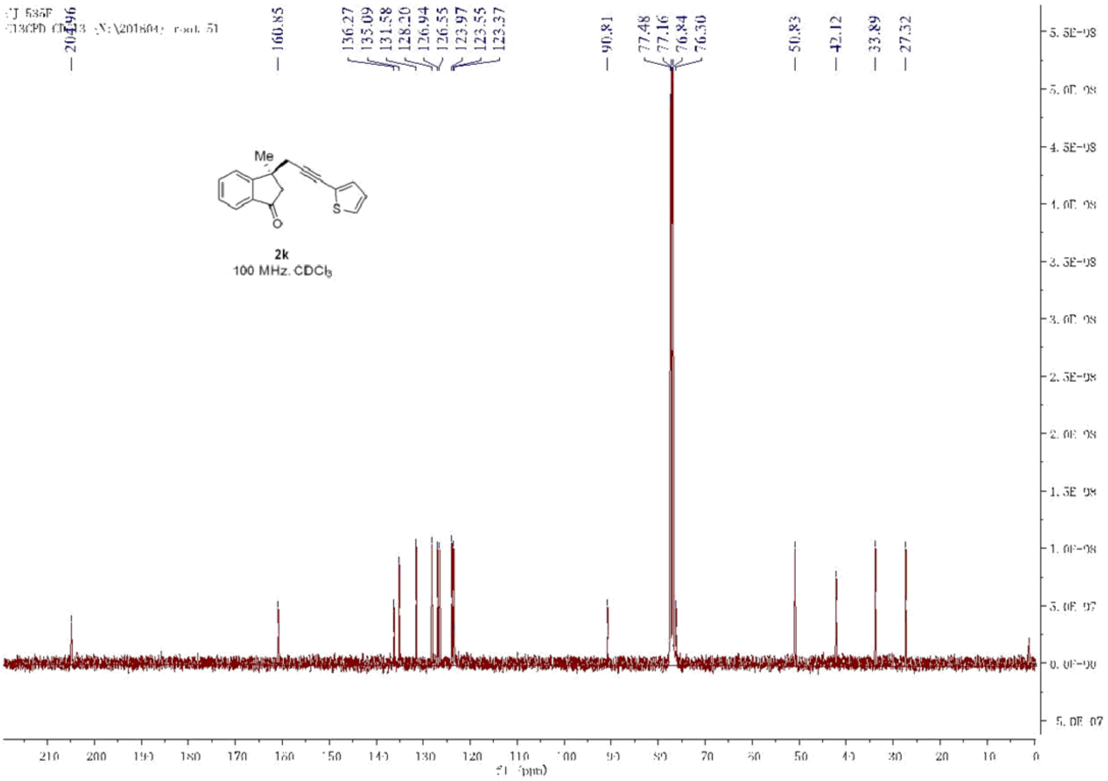

204.96
160.85
136.27
135.09
131.58
128.20
126.94
126.55
123.97
123.55
123.37
90.81
77.48
77.16
76.84
76.30
50.83
42.12
33.89
27.32
Me
S
O
2k
100 MHz, $CDCl_3$
f1 (ppm)

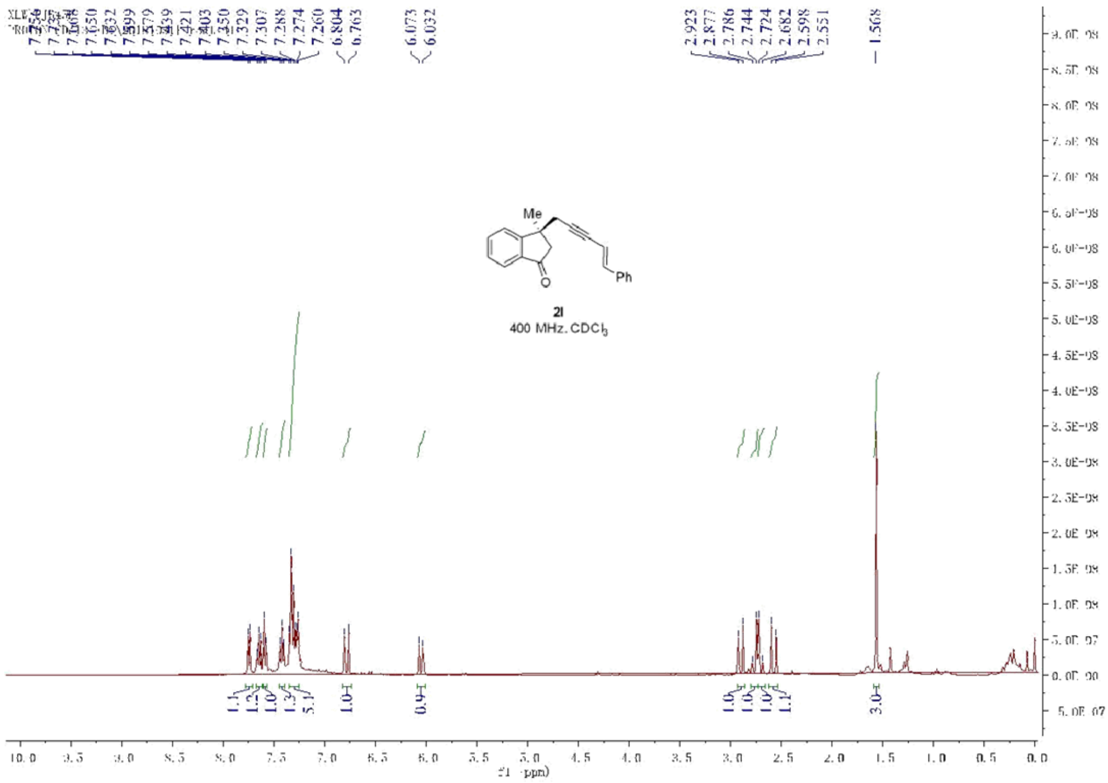
7.350 7.329 7.307 7.288 7.274 7.260 6.804 6.763 6.073 6.032
2.923 2.877 2.786 2.744 2.724 2.682 2.598 2.551 1.568
Me
O
Ph
2l
400 MHz, $CDCl_3$
f1 (ppm)

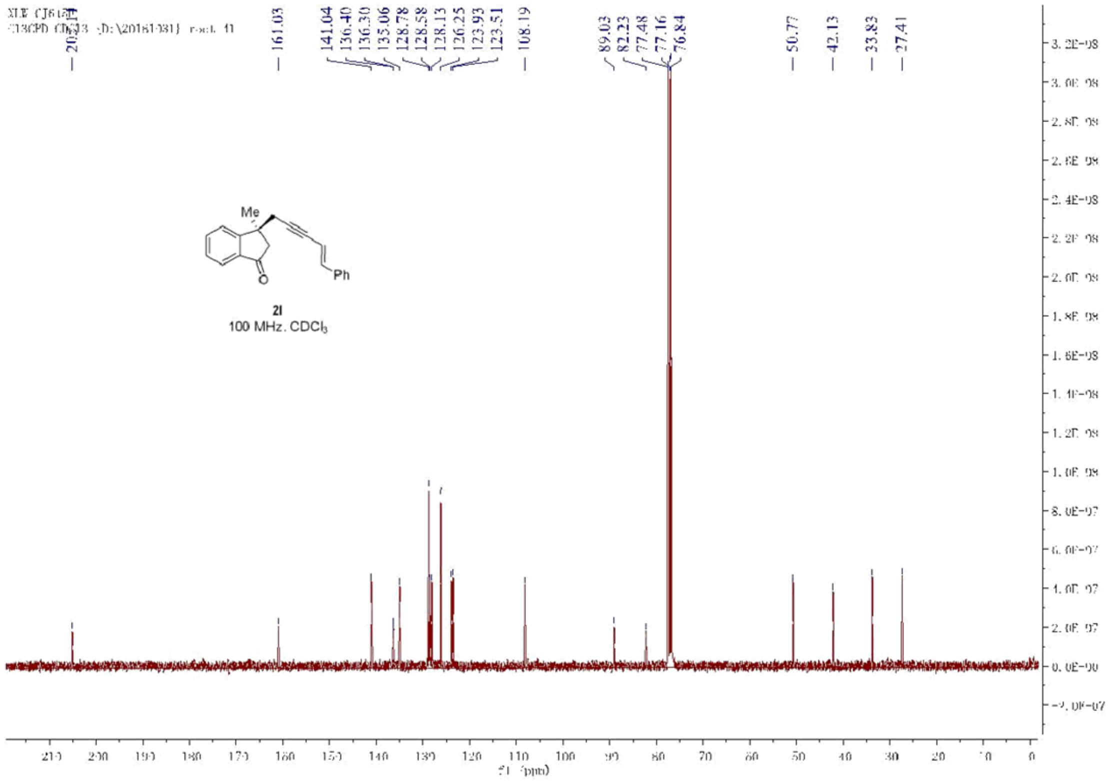
161.03 141.04 136.40 136.30 135.06 128.78 128.58 128.13 126.25 123.93 123.51 108.19
89.03 82.23 77.48 77.16 76.84 50.77 42.13 33.83 27.41
Me
O
Ph
2l
100 MHz, $CDCl_3$
f1 (ppm)

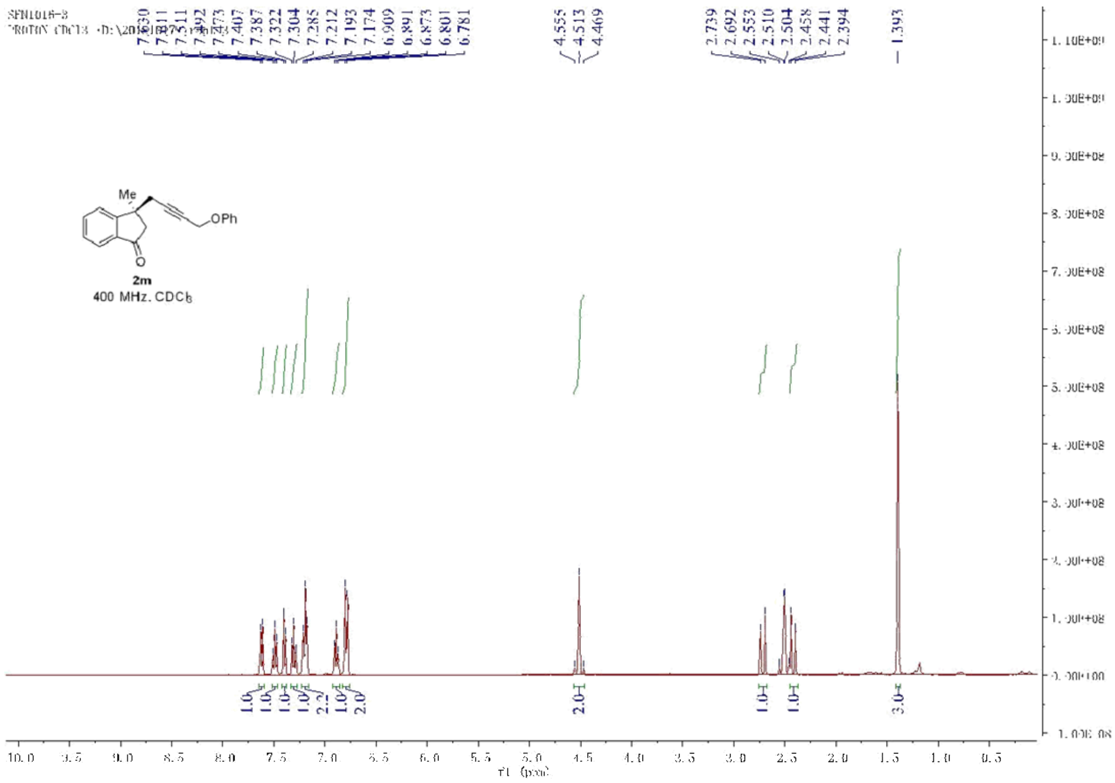
SFN1016-3
2m
400 MHz, CDCl3

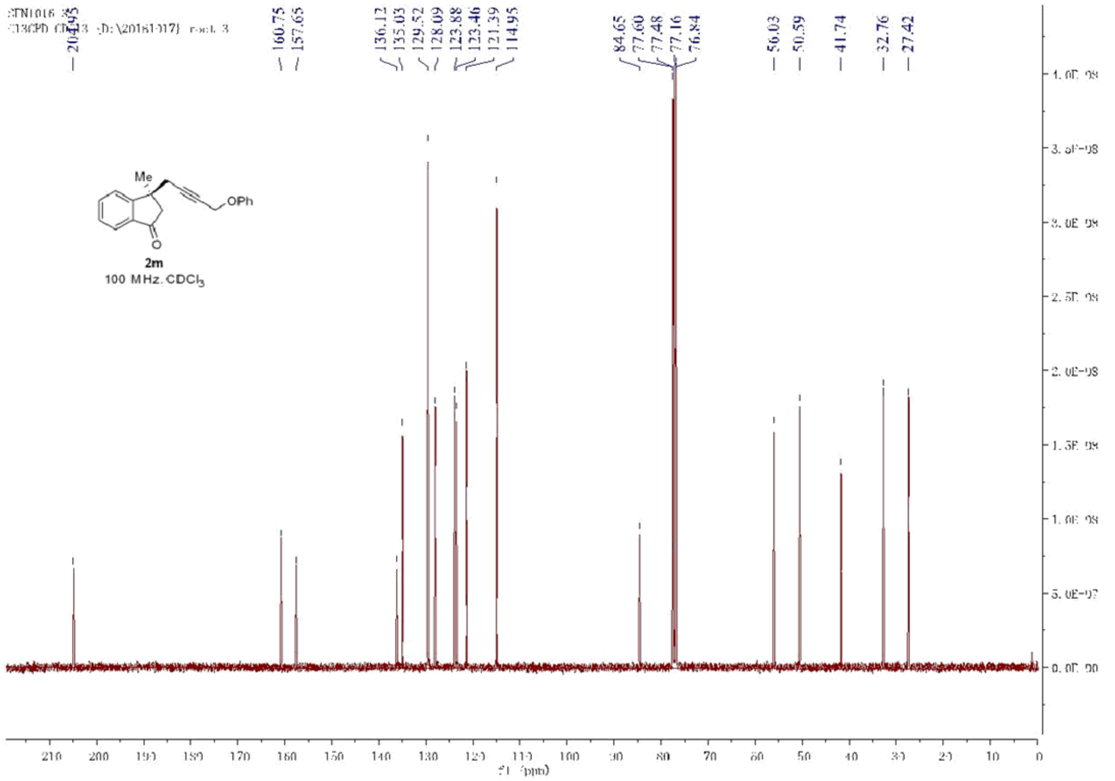
SFN1016-3
2m
100 MHz, CDCl3

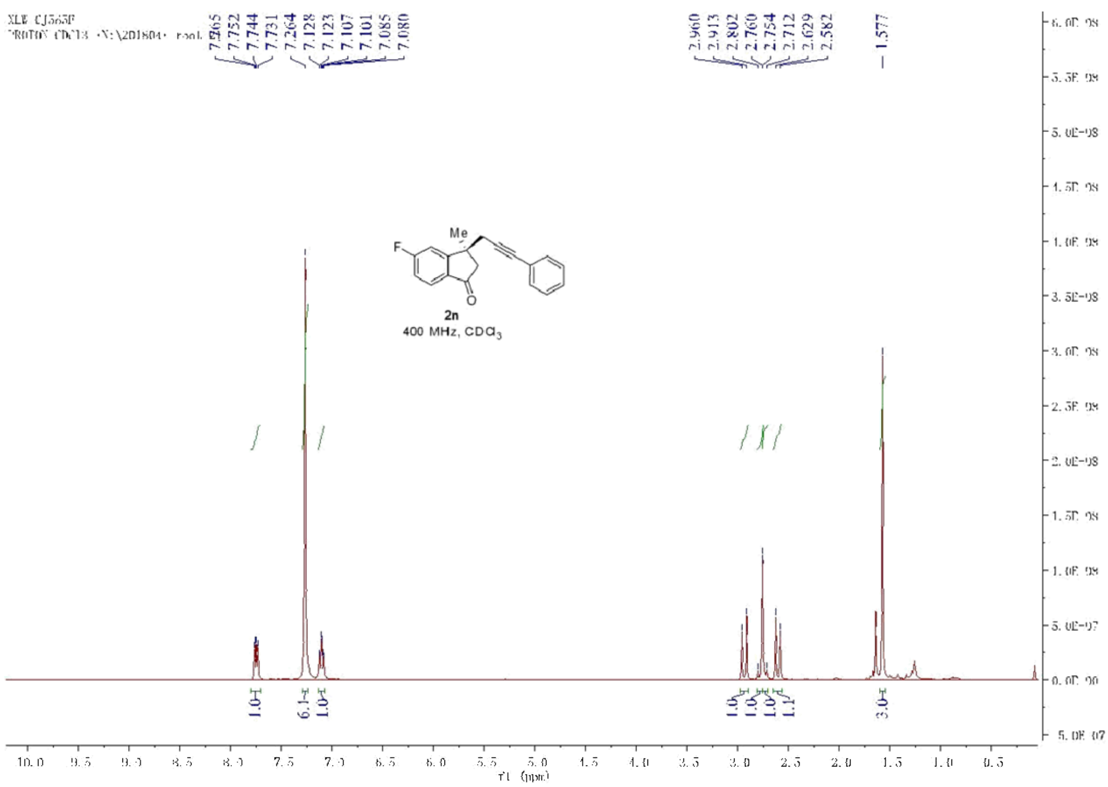
Me
F
O
2n
400 MHz, CDCl3
f1 (ppm)

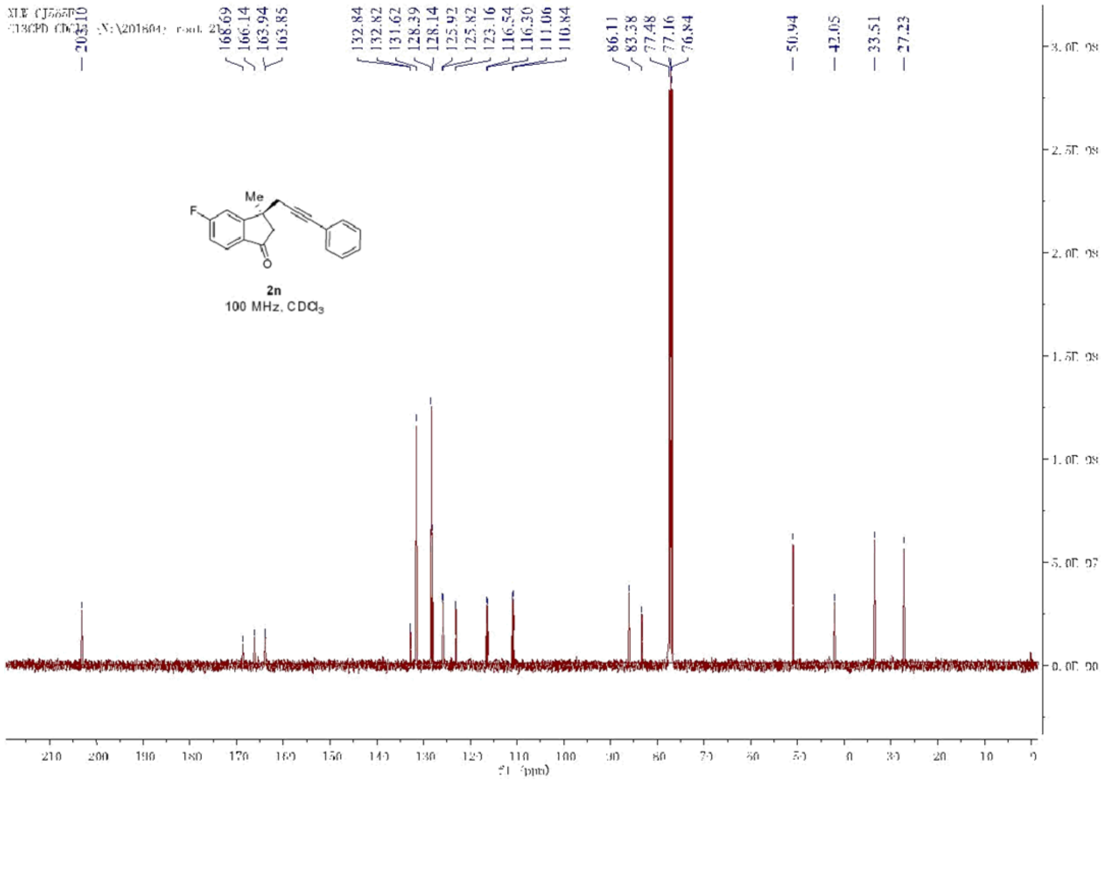
Me
F
O
2n
100 MHz, CDCl3
f1 (ppm)

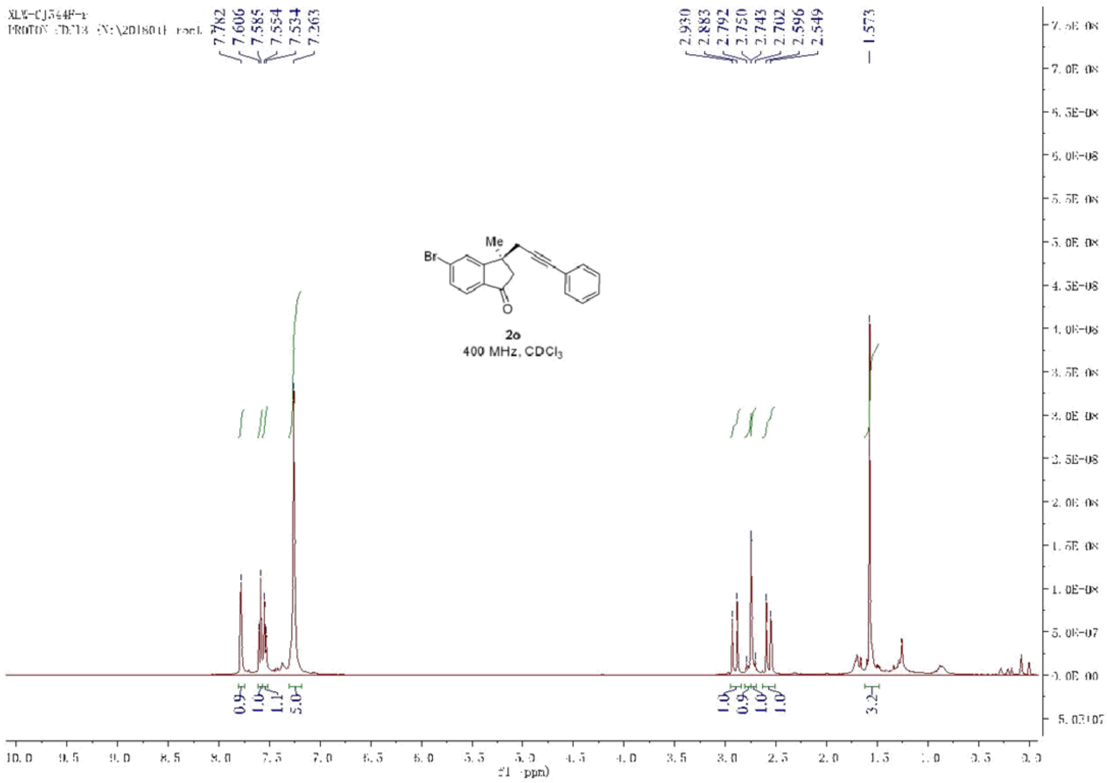
XLW-CJ544P-H
PROTON CDCl3
7.782
7.606
7.585
7.554
7.534
7.263
2.930
2.883
2.792
2.750
2.743
2.702
2.596
2.549
1.573
Br
Me
O
2o
400 MHz, CDCl3
0.9
1.0
1.1
5.0
1.0
0.9
1.0
1.0
3.2
f1 (ppm)

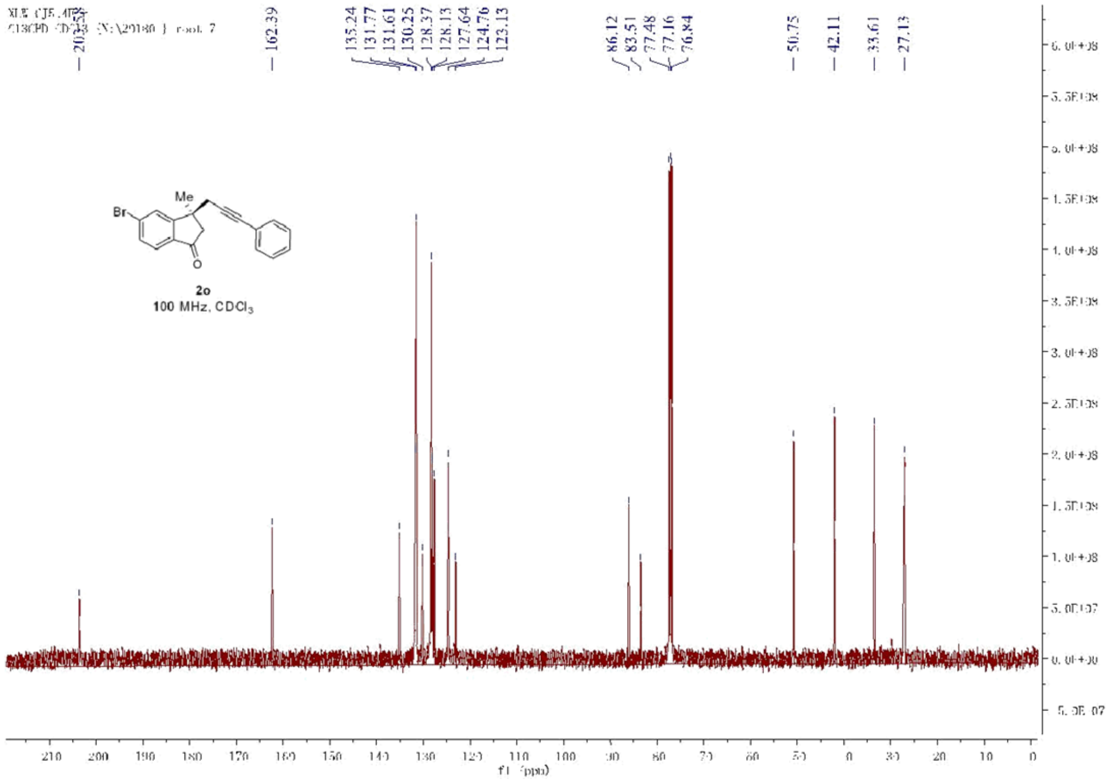
C13CPD CDCl3
162.39
135.24
131.77
131.61
130.25
128.37
128.13
127.64
124.76
123.13
86.12
83.51
77.48
77.16
76.84
50.75
42.11
33.61
27.13
Br
Me
O
2o
100 MHz, CDCl3
f1 (ppm)

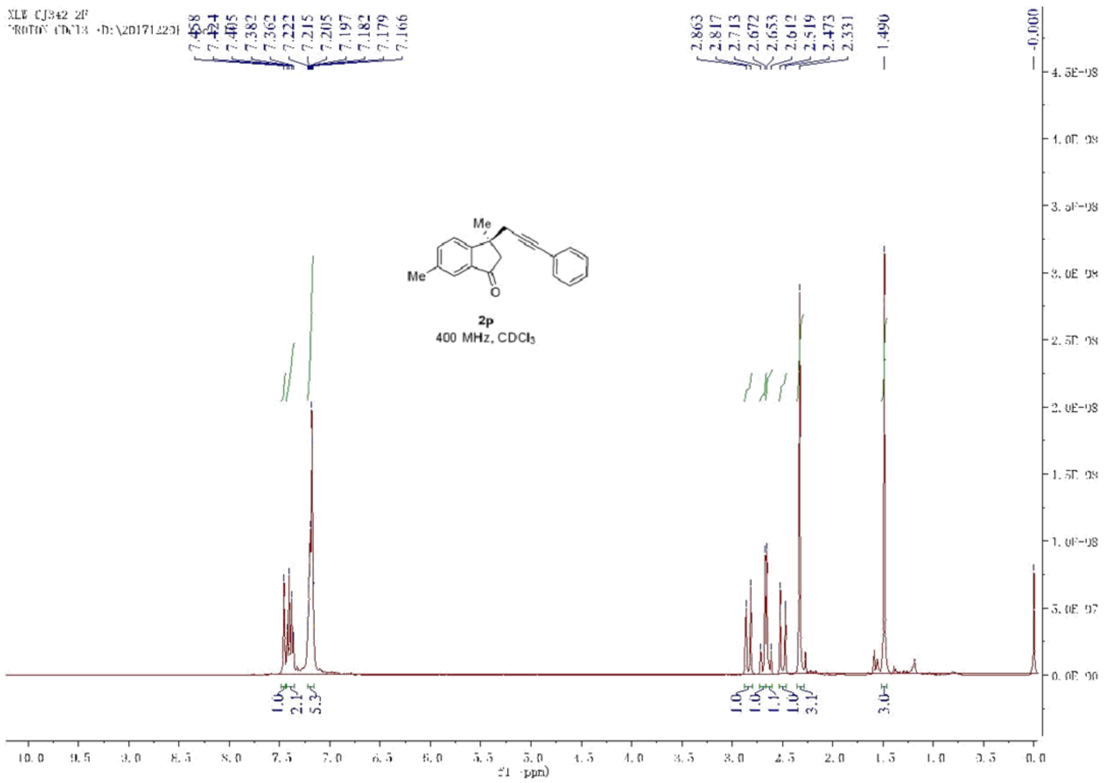
Me
Me
O
2p
400 MHz, CDCl3
f1 (ppm)

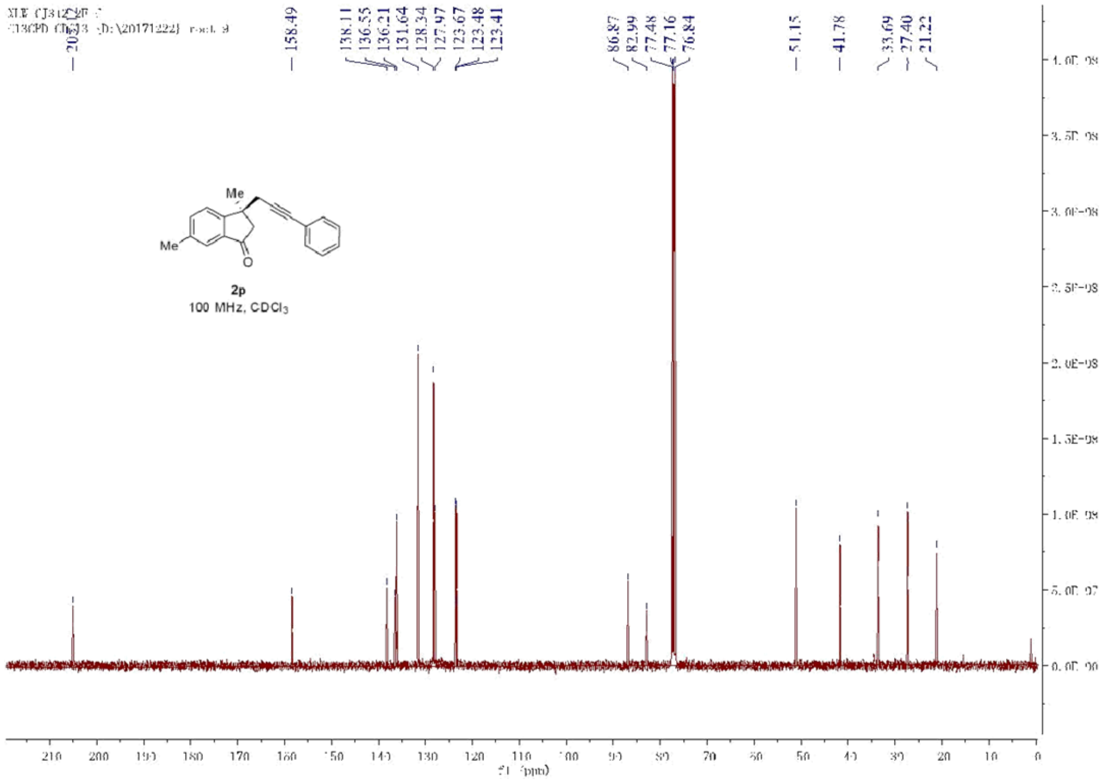
Me
Me
O
2p
100 MHz, CDCl3
f1 (ppm)

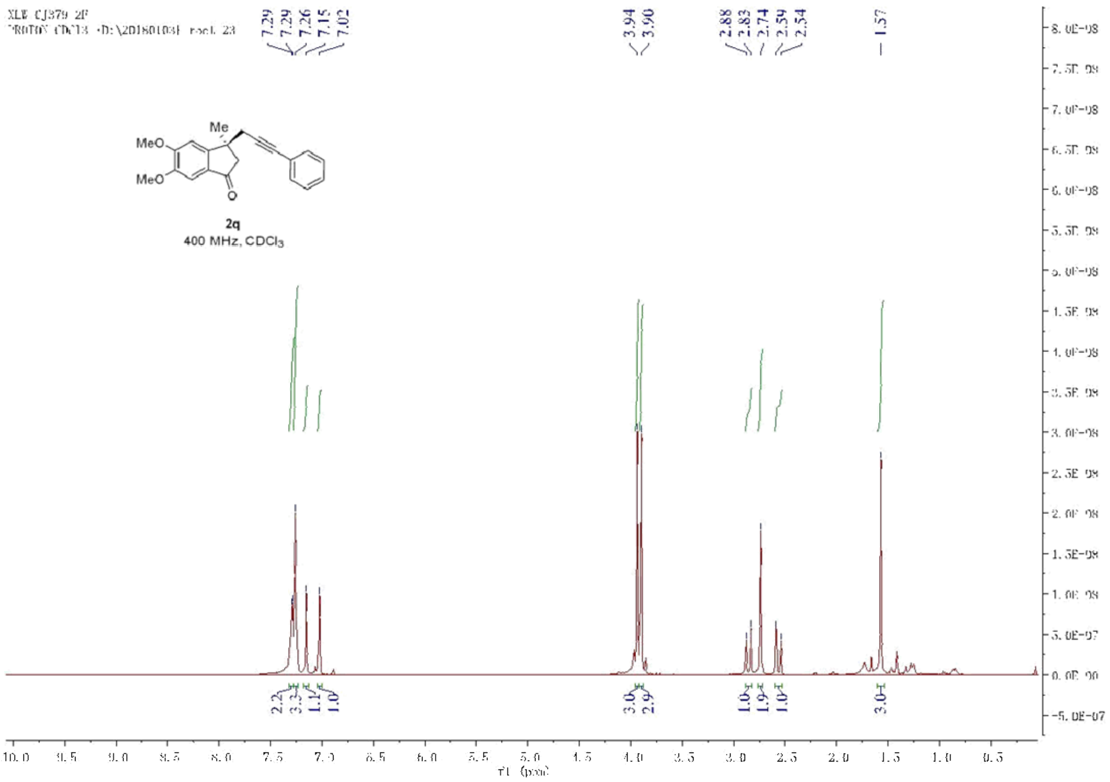

MeO
MeO
Me
O
2q
400 MHz, CDCl3

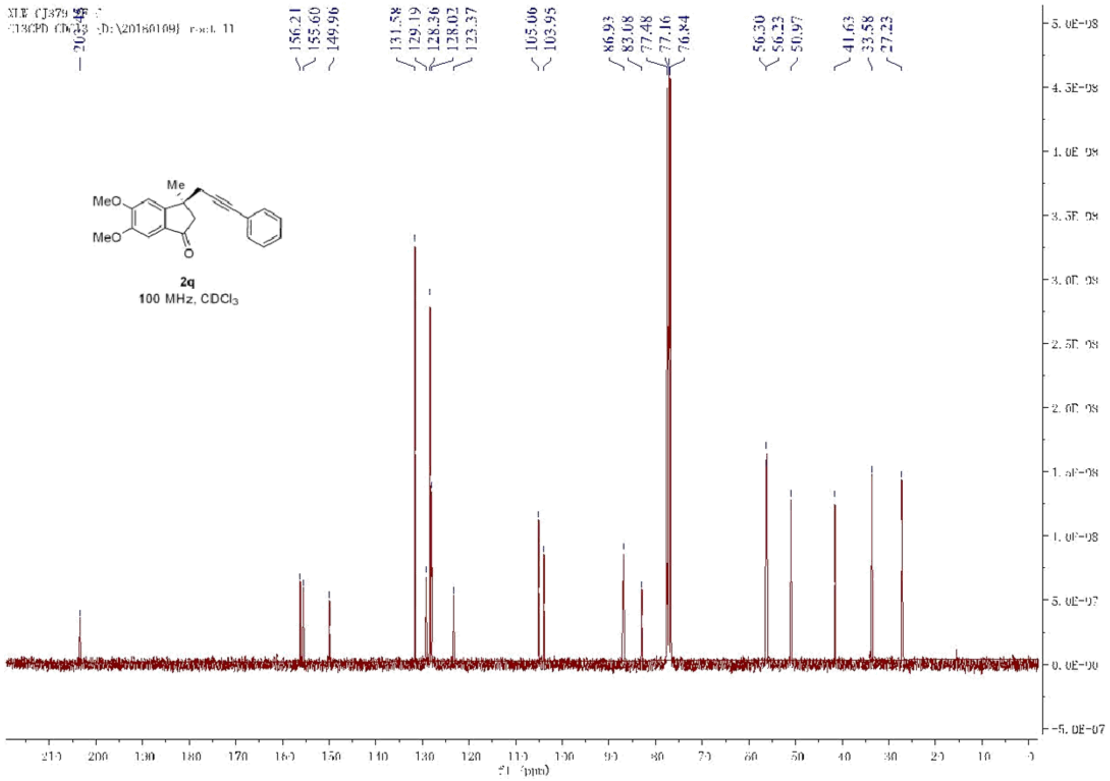

MeO
MeO
Me
O
2q
100 MHz, CDCl3

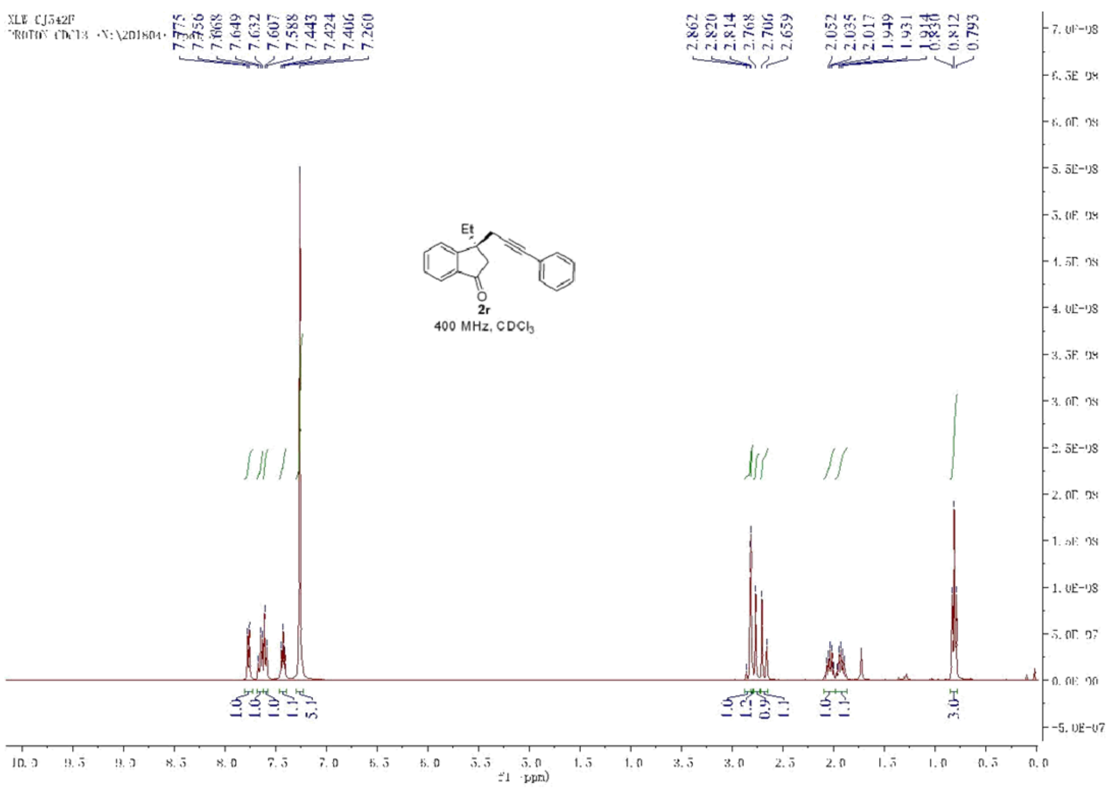
Et
2r
400 MHz, CDCl3
2.862
2.820
2.814
2.768
2.706
2.659
2.052
2.035
2.017
1.949
1.931
0.812
0.793
f1 (ppm)

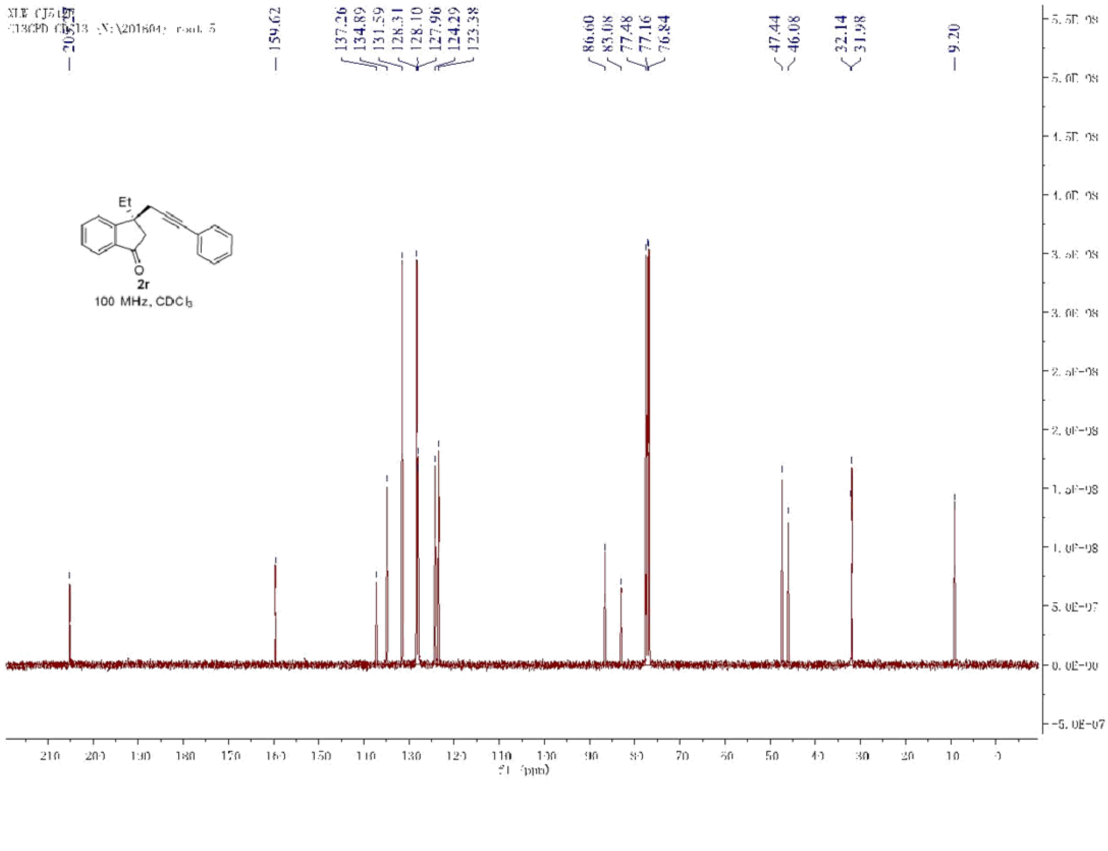
Et
2r
100 MHz, CDCl3
159.62
137.26
134.89
131.59
128.31
128.10
127.96
124.29
123.38
86.60
83.08
77.48
77.16
76.84
47.44
46.08
32.14
31.98
9.20
f1 (ppm)

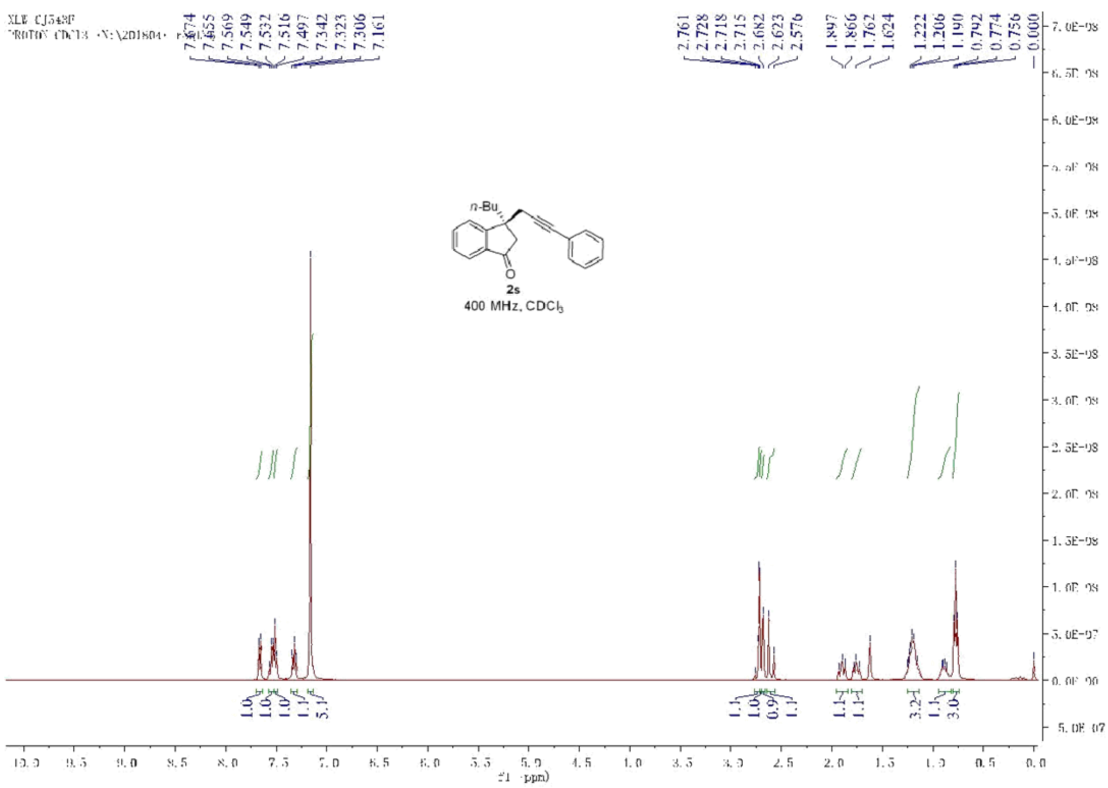
n-Bu
2s
400 MHz, CDCl3
f1 (ppm)

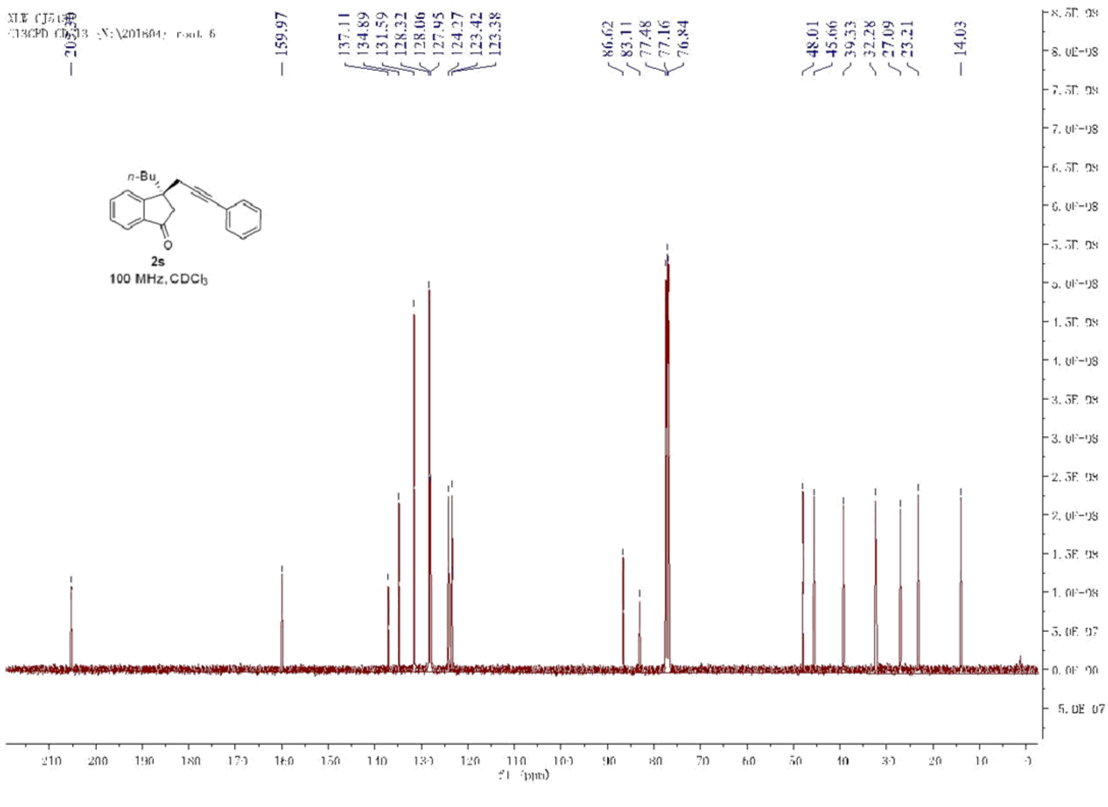
n-Bu
2s
100 MHz, CDCl3
f1 (ppm)

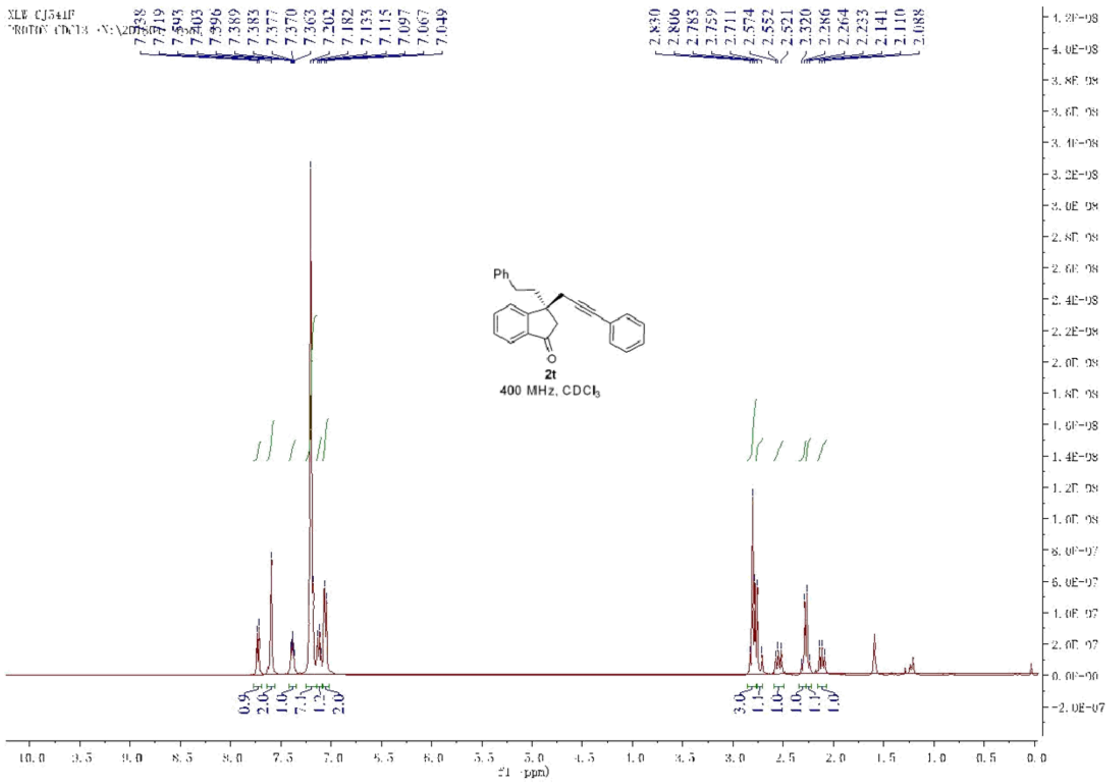

2t
400 MHz, CDCl3
7.738 7.719 7.593 7.403 7.396 7.389 7.383 7.377 7.370 7.363 7.202 7.182 7.133 7.115 7.097 7.067 7.049
2.830 2.806 2.783 2.759 2.711 2.574 2.552 2.521 2.320 2.286 2.264 2.233 2.141 2.110 2.088
0.9 2.0 1.0 7.1 1.2 2.0
3.0 1.1 1.0 1.0 1.1 1.0
f1 (ppm)

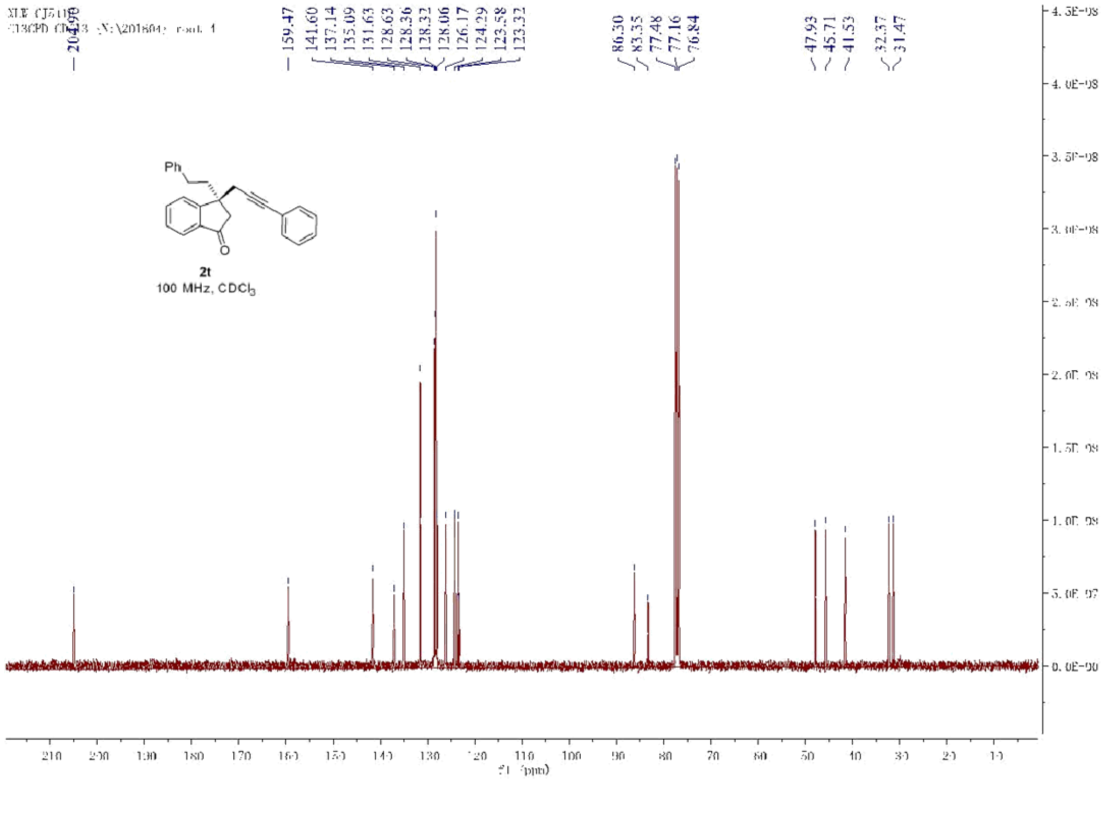

2t
100 MHz, CDCl3
204.90
159.47 141.60 137.14 135.09 131.63 128.63 128.36 128.32 128.06 126.17 124.29 123.58 123.32
86.30 83.35 77.48 77.16 76.84
47.93 45.71 41.53
32.37 31.47
f1 (ppm)

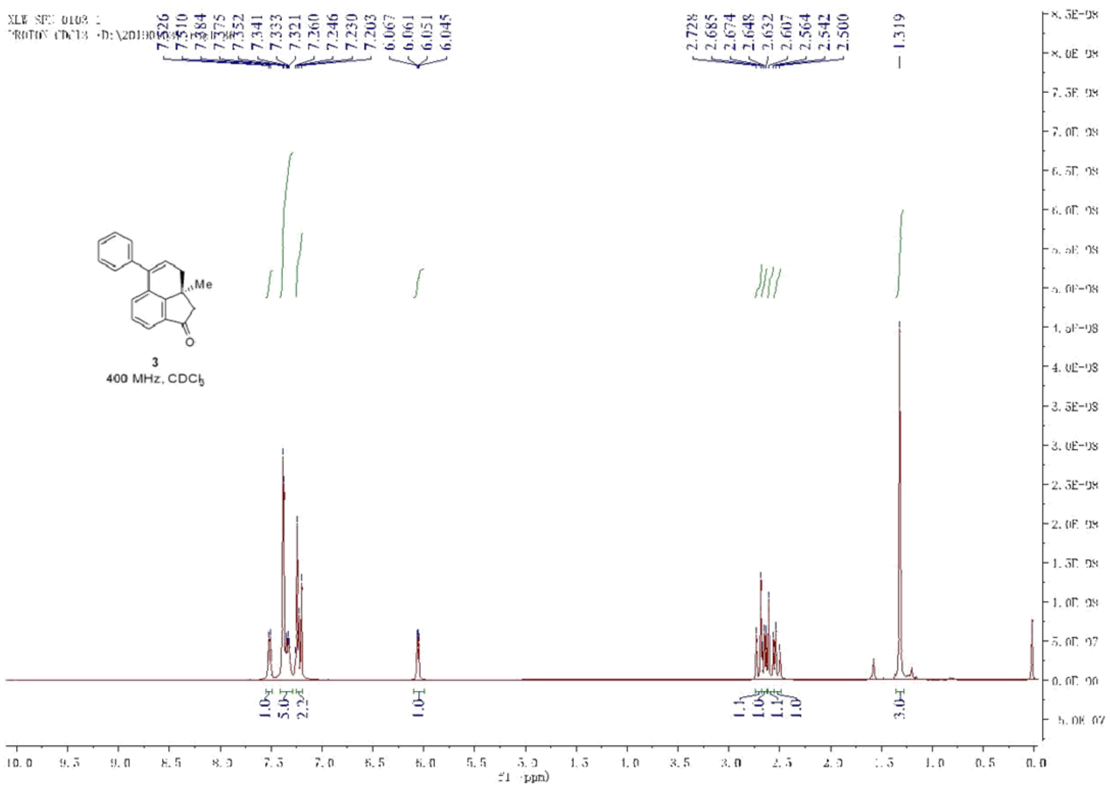
7.526 7.510 7.384 7.375 7.352 7.341 7.333 7.321 7.260 7.246 7.230 7.203 6.067 6.061 6.051 6.045
2.728 2.685 2.674 2.648 2.632 2.607 2.564 2.542 2.500
1.319
Me
3
400 MHz, CDCl3
1.0 5.0 2.2 1.0 1.1 1.0 1.1 1.0 3.0
f1 (ppm)

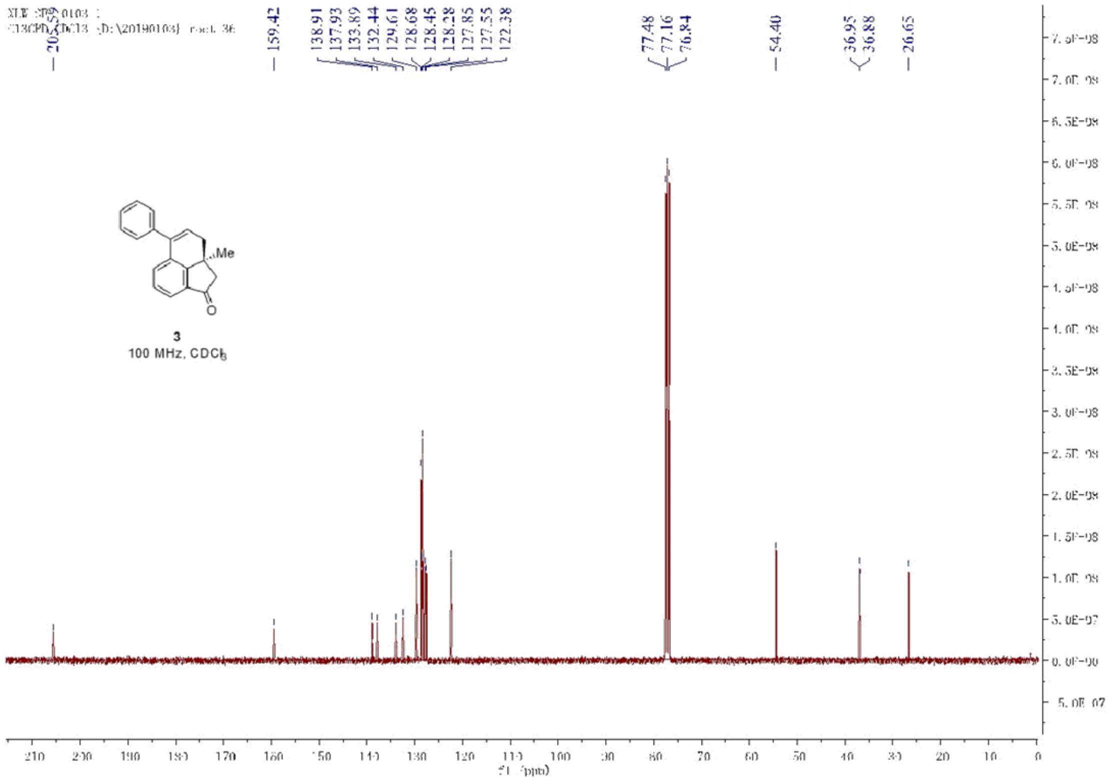
159.42
138.91 137.93 133.89 132.44 129.61 128.68 128.45 128.28 127.85 127.55 122.38
77.48 77.16 76.84
54.40
36.95 36.88
26.65
Me
3
100 MHz, CDCl3
f1 (ppm)
